# Mining differential top-k co-expression patterns from time course comparative gene expression datasets

**DOI:** 10.1186/1471-2105-14-230

**Published:** 2013-07-21

**Authors:** Yu-Cheng Liu, Chun-Pei Cheng, Vincent S Tseng

**Affiliations:** 1 Department of Computer Science and Information Engineering, National Cheng Kung University, No. 1, University Road, Tainan City 701, Taiwan R.O.C; 2 Department of Environmental and Occupational Health, College of Medicine, National Cheng Kung University, No. 1, University Road, Tainan City 701, Taiwan R.O.C; 3 Institute of Medical Informatics, National Cheng Kung University, No. 1, University Road, Tainan City 701, Taiwan R.O.C

## Abstract

**Background:**

Frequent pattern mining analysis applied on microarray dataset appears to be a promising strategy for identifying relationships between gene expression levels. Unfortunately, too many itemsets (co-expressed genes) are identified by this analysis method since it does not consider the importance of each gene within biological processes to a cellular response and does not take into account temporal properties under biological treatment-control matched conditions in a microarray dataset.

**Results:**

We propose a method termed *TIIM* (*Top-k Impactful Itemsets Miner*), which only requires specifying a user-defined number *k* to explore the top *k* itemsets with the most significantly differentially co-expressed genes between 2 conditions in a time course. To give genes different weights, a table with *impact degrees* for each gene was constructed based on the number of neighboring genes that are differently expressed in the dataset within gene regulatory networks. Finally, the resulting *top-k impactful itemsets* were manually evaluated using previous literature and analyzed by a Gene Ontology enrichment method.

**Conclusions:**

In this study, the proposed method was evaluated in 2 publicly available time course microarray datasets with 2 different experimental conditions. Both datasets identified potential itemsets with co-expressed genes evaluated from the literature and showed higher accuracies compared to the 2 corresponding control methods: i) performing *TIIM* without considering the gene expression differentiation between 2 different experimental conditions and *impact degrees*, and ii) performing *TIIM* with a constant *impact degree* for each gene. Our proposed method found that several new gene regulations involved in these itemsets were useful for biologists and provided further insights into the mechanisms underpinning biological processes. The Java source code and other related materials used in this study are available at “http://websystem.csie.ncku.edu.tw/TIIM_Program.rar”.

## Background

Identification of relationships between gene regulatory events is one of the main methods through which the biological effects of stimuli or changes in the environment are revealed. Microarrays are a highly efficient way to simultaneously measure the expression of massive numbers of genes. In these respects, multiple microarrays could be further used to quantify the expression of each gene during time course experiments. However, analysis and proper presentation of biological insights into these large-scale datasets is a big challenge.

Currently, frequent pattern-based mining analysis is widely used to identify groups of genes that are frequently co-expressed in most biological conditions in a microarray dataset. These methods include using the apriori algorithm [1], half-spaces [2], relational-based analysis [3], gene annotation integrated method [4], row enumeration-based method [5], column enumeration-based method [6], temporal-based method [7], rule induction [8], and FP-tree algorithm [9]. A gene itemset {*gene x*↑, *gene y*↓, *gene z*↑} states that upregulation of *gene x*, downregulation of *gene y* and upregulation of *gene z* frequently occur at the same time. *Support* is defined as the proportion of transactions in the data set that contain the itemset. Only gene itemsets with their *support* values no less than a user-set *minimum support threshold* can be defined as *frequent patterns*. Therefore, a gene itemset with a greater *support* value could have a high probability of becoming an interactome within a biological process. Although methods for traditional frequent pattern-based mining have been successfully proposed in previously published studies, these methods give the same weight to each gene during the execution process. In other words, these methods assume all genes have similar importance, which is often not in true in actual applications. Based on these challenges, some preceding studies on utility mining [10-17] have become predominant topics for solving these problems in the field of data mining.

The *utility* value of an itemset is the summation of each item *quantity* multiplied by its matched weight/importance in the co-expression transactions. An itemset is called a *high utility itemset* as long as its *utility* value is not less than a user-specified *minimum utility threshold*. However, traditional methods [10-17] for mining such *high utility itemsets* could not ensure that the items contained in a *high utility itemset* individually possess high *utility* values, since a longer itemset containing more items would have a higher *utility* value than shorter itemsets. To tackle this problem, a newer algorithm for mining average utility itemsets [18,19] was proposed; the discovered *utility itemsets* would be normalized with the number of items within the itemset. The resulting itemsets would be preserved, namely *high average utility itemsets*, as long as theirs *utility* values were not less than a user-specified *minimum average utility*. To the best of our knowledge, all of the above-mentioned methods could not be used to explore significantly differential itemsets between 2 different experimental conditions, e.g., biological treatment versus control, in time course datasets. How to choose those thresholds is also a big challenge; too many unpromising itemsets would be identified due to a lower threshold, whereas a strict threshold would yield few itemsets.

In this study, we propose a method called *TIIM* (*Top-k Impactful Itemsets Miner*) to identify the *top-k impactful itemsets* from time course comparative gene expression datasets. The proposed method only requires specifying a user-desired number *k* to explore the *k* most significantly differential gene itemsets between 2 experimental conditions on a microarray dataset. For each gene, the summation of frequencies at the same time point was defined as the *quantity*, and the number of neighboring genes that were differentially expressed in the dataset on the gene regulatory network (GRN) was defined as the *impact degree*, i.e., the importance of each gene. According to the *quantity* and *impact degree*, the *impactful itemsets* with most significant changes in gene expression can be efficiently explored. An *impactful itemset* considered more than just the node degrees (i.e., number of neighboring genes in the GRN) of each gene contained in the itemset. First, the *quantity* (transformed from the gene expression values) of each gene contained in an itemset was used as an important reference to calculate the *impactful value* of the itemset. Second, only the number (*impact degree*) of significant neighboring genes that were differently expressed between 2 comparative conditions in the microarray dataset on the GRN was calculated. Therefore, well-studied genes may not dominate others in terms of *impact degree*, i.e., well-studied genes may not always have higher *impact degrees* even if they had more neighboring genes in the GRN. Two baseline methods were considered as follows: i) performing *TIIM* without considering the deviations between gene expression levels of 2 experimental conditions and the *impact degree*, and ii) performing *TIIM* with a constant *impact degree* for each gene, and our proposed *TIIM* was performed on 2 real datasets from human and mouse microarrays.

The remainder of this paper is organized as follows: “Methods” provides some problem definitions and presents the proposed method. “Results and discussion” consists of the application of the approach to 2 real datasets in order to study the significance of the discovered *impactful itemsets*. Finally, we present conclusions based on our findings.

## Methods

**Figure 1:**
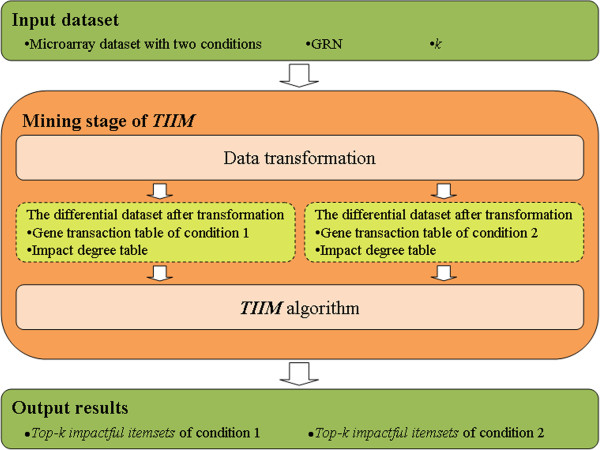
A flowchart of TIIM for discovering differential top-k impactful itemsets.

In this section, we first describe the *TIIM* (*Top-k Impactful Itemsets Miner*) algorithm as shown in Figure 1. Before we utilize the *TIIM* algorithm to discover the *top-k impactful itemsets*, microarray and GRN datasets must undergo a transformation process. Thereafter, research problem is defined. Finally, Section “TIIM” shows the proposed *TIIM* algorithm in detail.

### Gene expression data transformation

#### Conversion of gene expression into a transaction format

**Figure 2:**
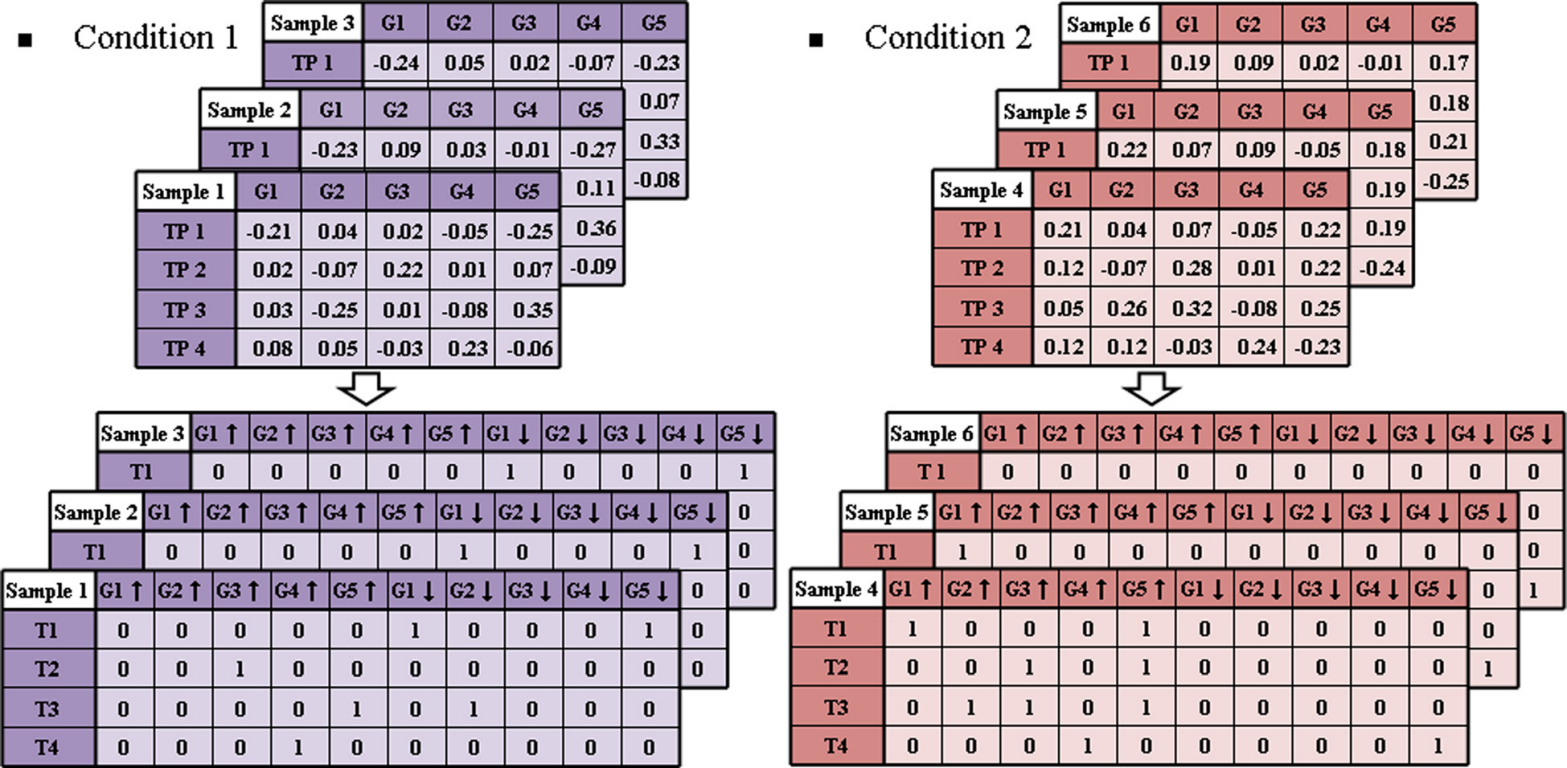
Example of transforming gene expression data into the transaction data format.

As with the frequent pattern-based method of gene expression data analysis proposed by Creighton and Hanash in 2003, each expression value in the dataset was transformed as up (↑; expressed; readings are greater than 0.2 for the log base 10 of fold-change 1.58 as an upper-bound), down (↓; repressed; readings are lower than −0.2 for the log base 10 of fold-change −1.58 as a lower-bound), or normal (neither expressed nor repressed). Based on most previous microarray analysis studies, the threshold value was set at a reasonable range (fold change from 1.5 to 2.0) to identify differentially expressed probes. Only the gene expression values transformed as up or down were preserved into the transaction dataset. Each time point was recognized as a transaction. After the process of transformation, gene x↑ (denoted as *G*_*x*_↑) and gene x ↓ (denoted as *G*_*x*_↓) were defined as 2 different gene items. An example is shown in Figure 2. Suppose that we have a dataset in which a detection of 5 genes (*G*_*1*_ to *G*_*5*_) at 4 time points is performed on 6 samples (triplicate for 2 conditions). For the first condition (*Condition 1*) dataset, *G*_*5*_ in sample 1 and time point 1 (*TP*_*1*_) was transformed into *G5*↓ in transaction 1 (*T*_*1*_) since its expression level was less than −0.2.

#### Transaction value integration step

**Figure 3:**
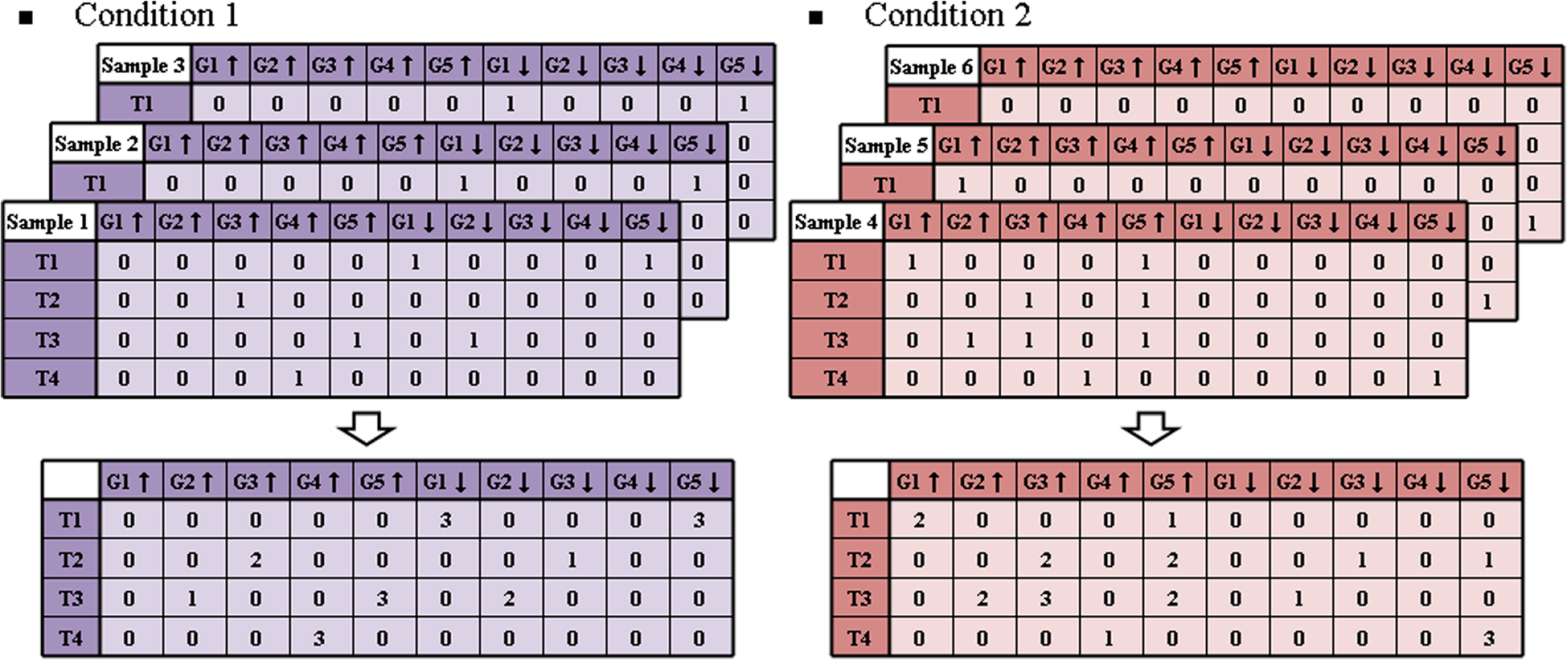
Example of integrating transformed gene item values over repeated samples.

In this step, for each gene item, the transformed values over repeated samples in the same transaction and condition were summed up as the *quantity*. Higher *quantities* represented stronger consistency and greater confidence in the gene items. Figure 3 gives an illustration of the transformed item value integration process. In the Condition 1 dataset of the above example, the *quantity* of *G*_*5*_↓ in *T*_*1*_ was 3.

#### Transaction value differentiation step

**Figure 4:**
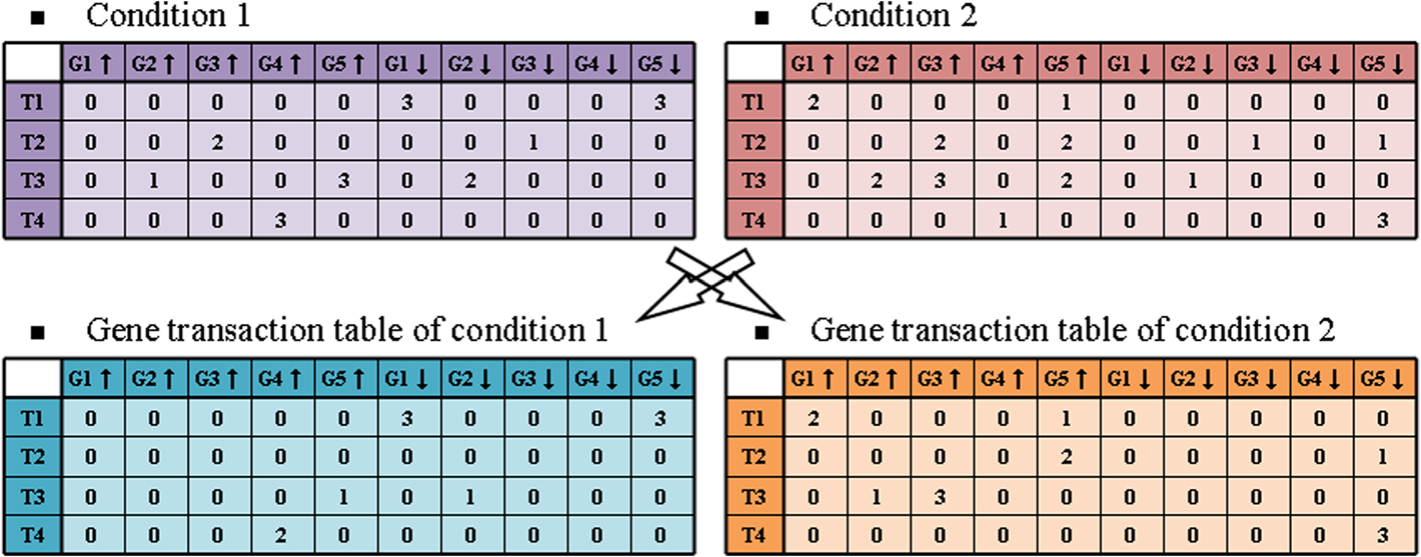
Example of identifying differential gene items.

The purpose of this study was to discover significantly differentially expressed gene itemsets between 2 different conditions. Here, we show how *quantity* differences for each gene item in every transaction were calculated for the 2 conditions. An example is shown in Figure 4. In this example, the *quantities* of *G*_*4*_↑ in *T*_*4*_ for the 2 conditions were 3 and 1, respectively. Since *G*_*4*_↑ had a greater *quantity* in Condition 1, the *quantity* difference 2 was assigned to *G*_*4*_↑ in *T4* of Condition 1, and the *quantity* 0 was assigned to *G*_*4*_↑ in *T*_*4*_ of Condition 2. In contrast, since the gene item *G*_*5*_↓ in *T*_*4*_ shown in Condition 2 had a greater *quantity* (3) than the matched gene in Condition 1 (0), the *quantity* difference 3 was predominant in Condition 2.

### Building the *impact degree* table

**Figure 5:**
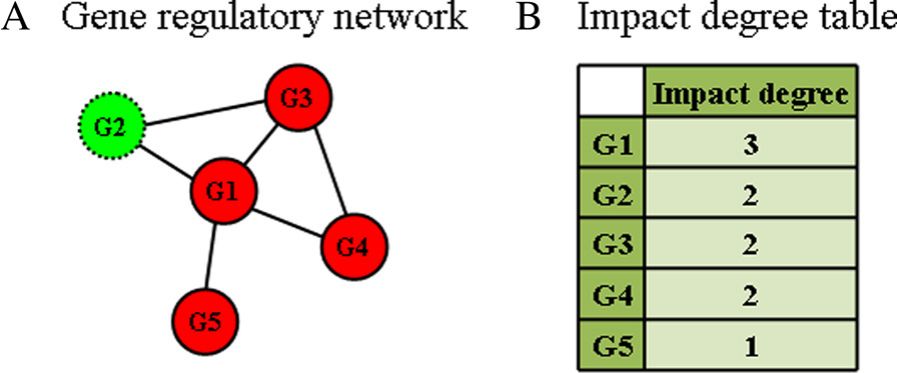
Generation of an impact degree table.

To generate a table with various weights of each gene, the number (*impact degree*) of significant neighboring genes that were differently expressed in the microarray dataset of the GRN was calculated. In Figure 5A, each node within the GRN represents a gene. If there is biological regulation between 2 genes, they are linked together. Student’s t-tests were performed to examine the expression of each gene for each of the 2 conditions at each time point. A gene was defined as a significant gene if it exhibited a significant change in expression (*p-value* < 0.05) between 2 conditions at any time point. In Figure 5A, 4 significant genes, i.e., *G*_*1*_, *G*_*3*_, *G*_*4*_, and *G*_*5*_, are shown; *G*_*2*_ was not a significant gene and is therefore presented as a dotted node. According to the aforementioned definition, *G*_*1*_ had 3 significant neighboring genes (*G*_*3*_, *G*_*4*_ and *G*_*5*_) on the GRN, and therefore the *impact degree* of *G*_*1*_ was defined as 3 in the *impact degree* table of this dataset (Figure 5B). In this study, the *impact degree* of *Gx* represented the *impact degree* of both *G*_*x*_↑ and *G*_*x*_↓ gene items.

### Basic definitions

Give a finite set of gene items *I* = {*i*_*1*_, *i*_*2*_, …, *i*_*m*_}. Each gene item *i*_*x*_ (1 ≤ *x* ≤ *m*) has an unique *impact degree d*(*i*_*x*_). A gene itemset *S* is a set of *l* distinct gene items, namely *l-itemset*; *l* is the length of *S*, denoted as *l*_*s*_. A gene transaction database was defined as *D* = {*T*_*1*_, *T*_*2*_, …, *T*_*n*_}. Each gene item *i*_*x*_ in the transaction *T*_*y*_ (1 ≤ *y* ≤ *n*) is associated with a unique *quantity q*(*i*_*x*_, *T*_*y*_).

#### Definition 1

The *impactful value* of a gene item *i*_*x*_ in *D* is denoted as *i*(*i*_*x*_) and defined as shown in Formula 1. For example, according to the gene transaction table of Condition 2 in Figure 4 and the *impact degree* table in Figure 5B, the *impactful value* of gene item *G*_*5*_↑ is *i*(*G*_*5*_↑) = (*d*(*G*_*5*_↑) × *q*(*G*_*5*_↑,*T*_*1*_)) + (*d*(*G*_*5*_↑) × *q*(*G*_*5*_↑,*T*_*2*_)) = 1 × 1 + 1 × 2 = 3.

(1) $$\begin{array}{ll} \;i\left(i_{x}\right)= & \;\sum_{i_{x}\subseteq T_{y}\wedge \mathrm{Ty}\in D}i\left(i_{x},T_{y}\right)\\ & =\sum_{i_{x}\subseteq T_{y}\wedge T_{y}\in D}d\left(i_{x}\right)\times q\left(i_{x},T_{y}\right) \end{array}$$

#### Definition 2

The *impactful value* of a gene itemset *S* in *D* is denoted as *i*(*S*) and defined as shown in Formula 2. The algorithm computes the *impactful value* of *S* and only considers transactions that contain *S*. For example, according to the gene transaction table of Condition 2 in Figure 4 and the *impact degree* table in Figure 5B, the *impactful value* of gene itemset {*G*_*1*_↑, *G*_*5*_↑} is *i*({*G*_*1*_↑, *G*_*5*_↑}) = (1 / *l*_*S*_) × (*d*(*G*_*1*_↑) × *q*(*G*_*1*_↑,*T*_*1*_) + *d*(*G*_*5*_↑) × *q*(*G*_*5*_↑,*T*_*1*_)) = (1 / 2) × (3 × 2 + 1 × 1) = 3.5.

(2) $$\begin{array}{ll} \;i\left(S\right)= & \;\sum_{S\subseteq T_{y}\wedge T_{y}\in D}i\left(S,T_{y}\right)\\ & =\frac{1}{l_{S}}\times \sum_{i_{x}\subseteq S\wedge S\subseteq T_{y}\wedge T_{y}\in D}d\left(i_{x}\right)\times q\left(i_{x},T_{y}\right) \end{array}$$

#### Definition 3

The *top-k* is the user-defined number of impactful gene itemsets. A gene itemset *S* is a *top-k impactful itemset* if *l*_*s*_ is greater than 1 and there are no more than *k* – 1 gene itemsets whose *impactful values* are greater than *S*.

#### Definition 4

The appearance pattern of a gene item *i*_*x*_ is a vector *A*_*x*_ = {*e*_*1*_, *e*_*2*_, …, *e*_*n*_} to record the presence or absence of each transaction *T*_*y*_ (1 ≤ *y* ≤ *n*) in *D*. The element *e*_*y*_ (1 ≤ *y* ≤ *n*) in *A*_*x*_ is recorded as 1 when *q*(*i*_*x*_, *Ty*) > 0; otherwise, it is recorded as 0.

#### Property 1

If the *impactful value* of a gene *l-itemset S* is greater than the smallest *impactful value* of the *top-k impactful itemsets*, it has at least a gene *l-1-itemset* contained in *S* within the *top-k impactful itemsets*.

##### TIIM

In this study, we propose the *TIIM* algorithm to identify impactful gene co-expression patterns through the TIIM algorithm from gene expression datasets. The main process of the algorithm is described as follows:

After the above processes, the algorithm can generate the *top-k impactful itemsets*. The *TIIM* contains 3 subroutines: ***checking 2-itemset in each cluster***, ***checking 2-itemset between clusters,*** and ***checking l-itemsets*** functions.


In this ***checking 2-itemset in each cluster*** function, all of the generated gene *2-itemsets* can be contained in the same transactions. In other words, in the *impactful value* computation process, the verification of the generated gene *2-itemset* contained in certain transactions is not required. Besides, by sorting gene items within one cluster, each gene item from top to bottom will be examined, whether its *impactful values* are larger than any one of the current *top-k impactful itemsets*. The redundant tests are eliminated by steps 10 to 13 to save a lot of time.


In the above function, according to Property 1, we only verify the *impactful value* of gene *l-itemset S* in which a gene *l-1-itemset S*_*v*_ in the *l-1 in top-k list* combines with a new item *i*_*u*_ from step 4 to 7.

As stated in section “TIIM”, the *TIIM* algorithm is more efficient since the verification of ineligible gene itemsets is not required.

### Results and discussion

To evaluate the performance of our proposed method, we compared it with 2 control methods as baselines. With respect to computational design, the meaning of our *TIIM*-derived patterns was different from that of traditional frequent pattern mining algorithms. Traditional frequent patterns cannot be transformed through any post processing. For each individual experimental condition, the co-expression genes could be discovered by traditional frequent pattern mining algorithms, but *TIIM* was proposed to identify the differential co-expression of genes between 2 comparative conditions (e.g., wild-type and mutant samples) during a time period. Therefore, it may not proper to compare these results in this study. On the other hand, technically, there were 2 ways to enforce performing such comparisons regardless of the meaning of patterns: i) compare patterns identified by different methods by tuning their optimal parameter values or ii) using the same parameter values among the comparative methods. The former is hard to perform due to the characteristics stated in the previous paragraph. The latter is also not feasible since there were no common parameters between our proposed *TIIM* and traditional frequent pattern mining methods. In spite of the limitations, we designed additional control methods, termed “Undifferentiation” and “Constant degree”. The former was similar to traditional frequent pattern algorithms and did not consider the “Transaction value differentiation step” shown in Figure 4 and the *impact degree* shown in Figure 5. The latter was used to assign a constant degree “1” to each gene if they were given non-zero *impact degrees* in the *impact degree table*.

In the first section below (“Dataset”), we provide a brief introduction to the gene regulatory data and 2 gene expression datasets for humans and mice. In the second section, titled “Evaluation with literature”, we present the evaluation results in which the identified *top-50 impactful itemsets* were manually evaluated using a survey of biological literature. Finally, in the section “GO enrichment analysis”, we made an attempt to analyze the biological characteristics of interesting genes derived from the top 50 to 200 gene itemsets to show that the itemset-contained genes correlated very well with the data from the original microarray experimental designs.

#### Datasets

Gene regulatory data from humans and mice were downloaded from the BioGRID [20] and KEGG [21] databases. The GRN of humans comprised 434 genes that interact with one another via 525 transcriptional regulation interactions. The GRN of mice consisted of 297 genes that interact with one another via 372 transcriptional regulation interactions.

We experimented with the proposed *TIIM* on 2 large-scale time course microarray datasets used in past studies. For the first data set, Yoshizuka *et al.* attempted to investigate the key endogenous gene expression profiles of cell cycle arrest in response to a long period of human immunodeficiency virus type 1 (HIV-1) Vpr overexpression [22]. A human gene expression microarray was used to tackle this issue. They compared the expression patterns of 21,794 genes in wild-type Vpr-expressing cells with the expression patterns of the same genes in mutant F72A/R73A-Vpr- or R80A-Vpr-expressing cells over 9 time points, including 0, 1, 2, 4, 6, 8, 12, 16, and 24 hours in 5 duplicate samples. For the second dataset used in this study, Sciuto *et al.* used a mouse model to design a genomic approach to observe genetic alterations involved in the process of reduction-oxidation in murine pulmonary tissues in response to exposure to carbonyl chloride (phosgene) [23]. Forty Crl:CD-1 (ICR) BR mice were exposed (whole-body) to either air or a concentration × time (c × t) amount of 32 mg/m^3^ (8 ppm) phosgene for 20 min (640 mg × min/m^3^). Lung tissue was collected from air- or phosgene-exposed mice at 0.5, 1, 4, 8, 12, 24, 48, and 72 hours post-exposure. Both microarray datasets could be readily retrieved from the Gene Expression Omnibus (GEO) database (http://www.ncbi.nlm.nih.gov/geo/) with accession numbers GSE2296 (human) and GSE2565 (mouse).

#### Evaluation with literature

An increasing number of studies on gene regulatory events have been conducted in response to high genetic associations relevant to most biological outcomes. According to the main focus of our method, since the genes involved in each itemset have a high probability of regulating or interacting with each other, the explored gene itemsets were then disassembled into a length of 2 as relationships in order to verify such regulation or interaction with the literature. In biology, genes involved in these relationships have some biological regulations/interactions that may occur through transcriptional regulation, post-transcriptional RNA processing or post-translational modification. In this regard, a big problem arises as to how many relationships are top priorities to be evaluated. Too many relationships would increase the difficulty of the evaluation process. Therefore, we manually scrutinized and validated numerous relationships between gene regulatory events derived from the *top-50 impactful itemsets* of the 2 individual datasets taken from the literature. For example, in Additional file 1: Table S6, a human dataset-derived gene itemset {BAX (581, 1181007_1) Up, KAT2B (8850, 1188483_1) Down, and TP53 (7157, 1193761_1) Down} in a length of 3 with an *impactful* value of 43.33 could be disassembled into 3 relationships of possible biological regulations/interactions, including {BAX (581) Up and KAT2B (8850) Down}, {BAX (581) Up, and TP53 (7157) Down} and {KAT2B (8850) Down and TP53 (7157) Down}. If a disassembled relationship has been reported in previous literature, the serial number of the corresponding paper is shown in the reference column. In contrast, serial numbers with an asterisk represent an opposite association between the relationship and evidence from the literature. In the current example, Zhao *et al.* demonstrated that depsipeptide caused little or no changes in the binding of human KAT2B protein and human TP53 protein in A549 cells [24]. However, opposite directions of gene expression for BAX and TP53 was not observed in previous studies since such studies have reported the following: human TP53 protein is necessary for activation of human BAX protein, which can be upregulated by human CCND1 protein in MCF7 cells [25]; human TP53 protein increases the expression of human BAX mRNA in Igrov1 cells [26]; and an S121F mutant form of human TP53 protein increases the transcription of the human BAX gene in Saos 2 cells [27]. In addition to these well-identified gene regulatory events, to the best of our knowledge, the regulations/interactions between human BAX and KAT2B have not yet been reported. Statistics for all of the evaluation results for human and mouse datasets are shown in Tables 1 and 2, respectively. Our proposed method, which considered the gene degrees in the GRNs, appeared to have dramatically higher accuracy compared to the 2 corresponding control methods in both datasets. In this regard, the disassembled relationships identified by our proposed method are more likely to be meaningful of the actual biology of the original experimental design. For example, in Table 1, the 70 new gene relationships discovered by our method may play dominant functional roles in the process of cell cycle arrest in response to overexpression of mutant Vpr (F72A/R73A), whereas 53 new genes relationships were significantly expressed under baseline conditions (i.e., wild-type Vpr). For the mouse dataset shown in Table 2, 39 gene relationships were intimately associated with certain redox pathways induced by exposure to the phosgene. However, well-studied genes may not dominantly contribute to high-utility itemsets due to their high neighbor count. We take the well-documented EP300 as an example; although the gene has the second highest degree number (18) in the raw GRN, it was not found in any discovered top-50 itemsets of the human dataset via our proposed *TIIM* algorithm. Taken together, our data demonstrated that using the proposed method to discover *impactful itemsets* allowed a great many new potential gene relationships to be efficiently identified. Through a literature evaluation process, these rules also showed higher accuracies compared to the 2 matched control methods. Therefore, the newly identified gene relationships may be valuable for biologists in terms of providing further insights into the mechanisms of time-dependent changes in gene expression.

**Table 1:** Evaluation of disassembled relations in human datasets from the literature

**Methods**	**Comparison**		**TP**	**TN**	**FP**	**FN**	**New**	**Sensitivity**	**Specificity**	**Accuracy**
Undifferentiation	Comparison 1	Wild-type	0(0)	0(0)	0(0)	0(0)	43(0)	0.00%	N/A	0.00%
		F72A/R73A	0(0)	0(0)	0(0)	0(0)	74(0)			
	Comparison 2	Wild-type	0(0)	0(0)	0(0)	0(0)	43(0)			
		R80A	0(0)	0(0)	0(0)	0(0)	32(0)			
Constant degree	Comparison 1	Wild-type	0(0)	0(0)	0(0)	0(0)	9(3)	37.50%	N/A	37.50%
		F72A/R73A	2(2)	0(0)	0(0)	0(0)	66(18)			
	Comparison 2	Wild-type	1(1)	0(0)	0(0)	5(3)	70(42)			
		R80A	0(0)	0(0)	0(0)	0(0)	65(25)			
Various degrees	Comparison 1	Wild-type	5	0	0	3	53	63.04%	N/A	63.04%
		F72A/R73A	13	0	0	5	70			
	Comparison 2	Wild-type	3	0	0	5	66			
		R80A	8	0	0	4	53			

Abbreviation: *TP* True positive, consistent with positive gene regulation in the literature, *TN* True negative, consistent with negative gene regulation in the literature, *FP* False positive, inconsistent with negative gene regulation in the literature, *FN* False negative, inconsistent with positive gene regulation in the literature; Sensitivity: # of TP / (# of TP + # of FN); Specificity: # of TN / (# of TN + # of FP); Accuracy: (# of TP + # of TN) / (# of TP + # of TN + # of FP + # of FN).

**Table 2:** Evaluation of disassembled relations in mouse datasets from the literature

**Methods**	**Comparison**	**TP**	**TN**	**FP**	**FN**	**New**	**Sensitivity**	**Specificity**	**Accuracy**
Undifferentiation	Air	0(0)	0(0)	0(0)	0(0)	24(0)	0.00%	N/A	0.00%
	Phosgene	0(0)	0(0)	0(0)	0(0)	30(0)			
Constant degree	Air	1(0)	0(0)	0(0)	1(0)	63(0)	50.00%	N/A	50.00%
	Phosgene	0(0)	0(0)	0(0)	0(0)	12(0)			
Various degrees	Air	5(0)	0(0)	0(0)	0(0)	59(0)	71.43%	N/A	71.43%
	Phosgene	5(0)	0(0)	0(0)	4(0)	39(0)			

Abbreviation: *TP* True positive, consistent with positive gene regulation in the literature, *TN* True negative, consistent with negative gene regulation in the literature, *FP* False positive, inconsistent with negative gene regulation in the literature, *FN* False negative, inconsistent with positive gene regulation in the literature; Sensitivity: # of TP / (# of TP + # of FN); Specificity: # of TN / (# of TN + # of FP); Accuracy: (# of TP + # of TN) / (# of TP + # of TN + # of FP + # of FN).

#### GO enrichment analysis

In the previous section, although the identified gene relationships were manually evaluated with the literature, proving that the unreported/unpublished relationships derived from different *top-k itemsets* are reliable remained a challenge. In this section, we attempted to analyze all of the gene relationships shown in Tables 3 and 4 derived from the top 50 to 200 itemsets of each comparison for both species.

**Table 3:** Statistics for different impactful itemsets in human datasets

	**Comparisons**	**Datasets**	**Top-50**	**Top-100**	**Top-150**	**Top-200**
Average length of itemsets	Comparison 1^†^	Wild-type	3.54	4.38	4.93	5.18
		F72A/R73A	3.30	3.40	3.63	3.54
	Comparison 1^††^	Wild-type	3.38	3.76	4.36	4.80
		F72A/R73A	2.86	2.88	2.99	3.00
	Comparison 1^†††^	Wild-type	2.76	2.87	2.99	3.09
		F72A/R73A	2.90	2.88	2.94	2.96
	Comparison 2^†^	Wild-type	3.54	4.38	4.93	5.18
		R80A	3.24	3.93	3.96	3.96
	Comparison 2^††^	Wild-type	3.16	3.34	3.36	3.39
		R80A	2.98	3.00	3.27	3.24
	Comparison 2^†††^	Wild-type	2.92	2.99	3.00	3.00
		R80A	2.84	2.92	2.95	2.96
Number of disassembled relations	Comparison 1^†^	Wild-type	43	73	85	108
		F72A/R73A	74	132	178	251
	Comparison 1^††^	Wild-type	9	11	11	11
		F72A/R73A	68	125	178	229
	Comparison 1^†††^	Wild-type	61	154	207	226
		F72A/R73A	88	168	211	264
	Comparison 2^†^	Wild-type	43	73	85	108
		R80A	32	58	85	111
	Comparison 2^††^	Wild-type	76	136	183	232
		R80A	65	118	169	203
	Comparison 2^†††^	Wild-type	74	156	213	255
		R80A	65	138	204	259

†: Undifferentiation; ††: A constant degree; †††: Various degrees.

**Table 4:** Statistics for different impactful itemsets in mouse datasets

	**Comparisons**	**Datasets**	**Top-50**	**Top-100**	**Top-150**	**Top-200**
Average length of itemsets	Comparison 3^†^	Air	3.80	3.84	4.24	4.07
		Phosgene	3.68	3.93	4.21	4.35
	Comparison 3^††^	Air	2.68	2.77	2.77	2.83
		Phosgene	3.72	4.08	4.45	4.53
	Comparison 3^†††^	Air	2.82	2.94	2.99	3.03
		Phosgene	2.62	2.81	2.90	2.96
Number of disassembled relations	Comparison 3^†^	Air	24	40	74	74
		Phosgene	30	44	52	55
	Comparison 3^††^	Air	65	128	179	239
		Phosgene	13	39	52	65
	Comparison 3^†††^	Air	64	112	158	190
		Phosgene	48	93	140	196

†: Undifferentiation; ††: A constant degree; †††: Various degrees.

**Figure 6:**
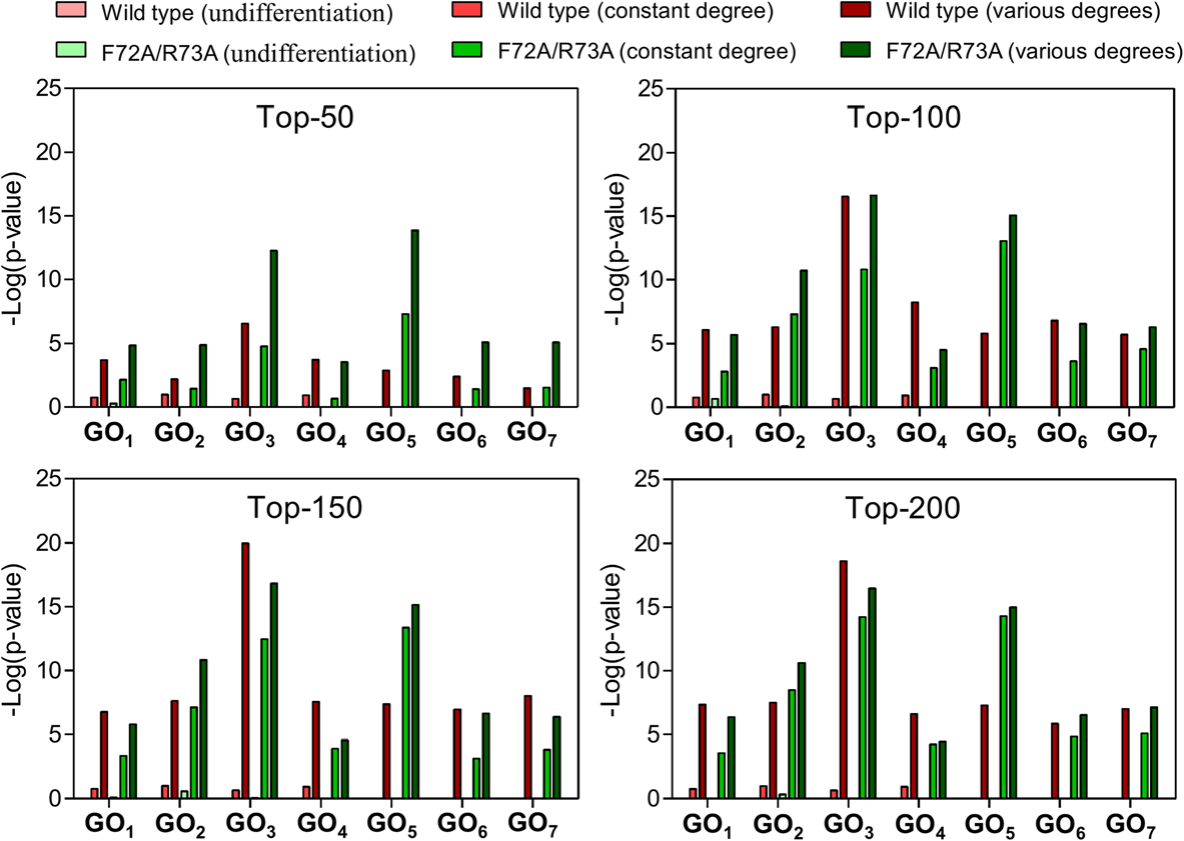
**GO enrichment analysis of wild-type and F72A/R73A mutant Vpr protein for the human dataset.** GO_1_: GO:0006915 ~ apoptosis; GO_2_: GO:0043066 ~ negative regulation of apoptosis; GO_3_: GO:0042127 ~ regulation of cell proliferation; GO_4_: GO:0008284 ~ positive regulation of cell proliferation; GO_5_: GO:0007050 ~ cell cycle arrest; GO_6_: GO:0007346 ~ regulation of mitotic cell cycle; GO_7_: GO:0051726 ~ regulation of cell cycle.

**Figure 7:**
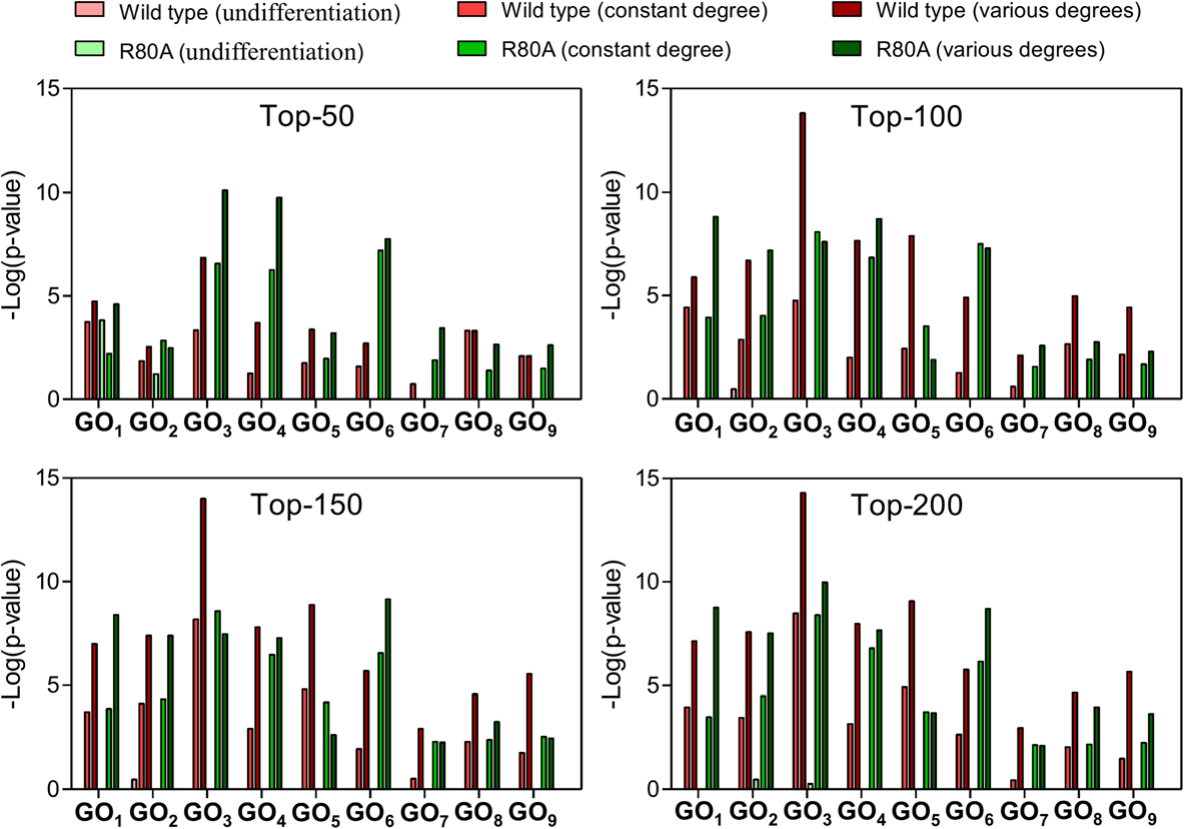
**GO enrichment analysis of wild-type and R80A mutant Vpr protein for the human dataset.** GO_1_: GO:0043066 ~ negative regulation of apoptosis; GO_2_: GO:0006915 ~ apoptosis; GO_3_: GO:0042127 ~ regulation of cell proliferation; GO_4_: GO:0008285 ~ negative regulation of cell proliferation; GO_5_: GO:0008284 ~ positive regulation of cell proliferation; GO_6_: GO:0007050 ~ cell cycle arrest; GO_7_: GO:0045786 ~ negative regulation of cell cycle; GO_8_: GO:0007346 ~ regulation of mitotic cell cycle; GO_9_: GO:0051726 ~ regulation of cell cycle.

Gene Ontology (GO) is useful for analyzing the biological characteristics of a set of genes, including biological processes, cellular components, and molecular functions [28]. To test the enrichment of cell cycle-related terms for the genes identified in the human dataset and the redox-related terms for the genes identified in the mouse dataset, all of the lists of gene relationships were separately uploaded to the DAVID bioinformatics analysis tool [29] and analyzed with a web-based functional annotation tool. For each uploaded gene list, we selected and examined the *p-value* of the terms associated with the original experimental results for all items categorized as “GOTERM_BP_4”. To make a statistically significant comparison for each GO term in the different experimental conditions, we only focused on the GO terms that had at least one *p-value* less than 0.05. The original paper had already demonstrated that the human cell cycle could be altered by the HIV-1 Vpr protein. Hence, we tested whether our identified results were associated with the cell cycle. Genes involved in *top-k* gene itemsets that were identified by considering various gene degrees had overall dramatically high cell cycle enrichments compared to the matched control experiments with 2 baseline methods in both comparisons including wild-type versus F72A/R73A and wild-type versus R80A (Figures 6 and 7). For the mouse dataset, the experiment results of the original paper indicated that redox pathways played functional roles in response to exposure to the phosgene. However, we could not obtain any of the eligible GO terms. This may have been due to the following factors: (i) insufficient gene regulation in the GRN: additional gene regulatory events have now been identified and further deposited in the online databases; (ii) probe reading noise is error-prone and inherent to the microarray-based measurement of gene expression [30]; and (iii) GO term enrichment analysis is more suitable for analyzing a set of distinct genes instead of the relationships between certain genes. In spite of these limitations, we still proved that our identified gene relationships exhibited good performance in both datasets via the literature survey illustrated in the previous section. The proposed method also could be applied to other topics of interest.

### Conclusions

In this study, we proposed the *TIIM* algorithm to discover *top-k impactful itemsets* with stronger meanings in biology from 2 gene expression datasets to address the flaws of previous frequent pattern analysis methods. Our method adopted a *top-k* strategy by introducing a user-selected *k* to avoid producing redundant insignificant itemsets (below the top-*k* itemset). During the evaluation process, the gene relationships derived from the *top-50* gene itemsets were manually verified with previous literature. This yielded higher accuracies in both microarray datasets compared to the 2 baseline methods. Moreover, GO term enrichment analysis also showed that our identified genes correlated very well with the original literature identifications. These good performance results may be attributed to the consideration of the number of differential neighboring genes in GRNs that could be easily retrieved from the inherent biological involvement of each array-involved gene. Our proposed method is therefore an effective means to provide biologists with further insights into the relationships of gene regulatory events and interactions in certain biological processes.

### Competing interests

The authors declare that they have no competing interests.

### Authors’ contributions

YCL and CPC wrote the paper. YCL developed the software and conducted the original experiments. YCL and CPC conceived and designed the experiments. YCL and CPC analyzed the experimental results. VST supervised the whole study. YCL, CPC, and VST read and approved the final manuscript.

## Supplementary Material

Additional file 1 Detailed top-50 impactful itemsets and literature evaluation results. Click here for file

## References

[B1] Creighton C Hanash S Mining gene expression databases for association rules Bioinformatics 2003 19 1 79 86 10.1093/bioinformatics/19.1.79 12499296

[B2] Georgii E Richter L Ruckert U Kramer S Analyzing microarray data using quantitative association rules Bioinformatics 2005 21 Suppl 2 ii123 ii129 10.1093/bioinformatics/bti1121 16204090

[B3] Liu YC Cheng CP Tseng VS Discovering relational-based association rules with multiple minimum supports on microarray datasets Bioinformatics 2011 27 22 3142 3148 10.1093/bioinformatics/btr526 21926125

[B4] Martinez R Pasquier N Pasquier C GenMiner: mining non-redundant association rules from integrated gene expression data and annotations Bioinformatics 2008 24 22 2643 2644 10.1093/bioinformatics/btn490 18799482

[B5] McIntosh T Chawla S High confidence rule mining for microarray analysis IEEE/ACM transactions on computational biology and bioinformatics / IEEE, ACM 2007 4 4 611 623 17975272 10.1109/tcbb.2007.1050

[B6] Carmona-Saez P Chagoyen M Rodriguez A Trelles O Carazo JM Pascual-Montano A Integrated analysis of gene expression by Association Rules Discovery BMC Bioinforma 2006 7 54 10.1186/1471-2105-7-54 PMC1386712 16464256

[B7] Nam H Lee K Lee D Identification of temporal association rules from time-series microarray data sets BMC Bioinforma 2009 10 3 S6 10.1186/1471-2105-10-S3-S6 PMC2665054 19344482

[B8] Tran DH Satou K Ho TB Finding microRNA regulatory modules in human genome using rule induction BMC Bioinforma 2008 9 12 S5 10.1186/1471-2105-9-S12-S5 PMC2638145 19091028

[B9] Chen Q Chen YP Mining frequent patterns for AMP-activated protein kinase regulation on skeletal muscle BMC Bioinforma 2006 7 394 10.1186/1471-2105-7-394 PMC1574354 16939655

[B10] Ahmed CF Tanbeer SK Jeong B-S Lee Y-K Efficient tree structures for high utility pattern mining in incremental databases IEEE Trans on Knowl and Data Eng 2009 21 12 1708 1721

[B11] Chan R Yang Q Shen Y-D Mining high utility itemsets Proceedings of the Third IEEE International Conference on Data Mining. 952150 2003 Melbourne, Florida, USA: IEEE Computer Society 19 26

[B12] Erwin A Gopalan RP Achuthan NR Efficient mining of high utility itemsets from large datasets Proceedings of the 12th Pacific-Asia conference on Advances in knowledge discovery and data mining 2008 1786628 Osaka, Japan: Springer-Verlag 554 561

[B13] Li Y-C Yeh J-S Chang C-C Isolated items discarding strategy for discovering high utility itemsets Data Knowl Eng 2008 64 1 198 217 10.1016/j.datak.2007.06.009

[B14] Liu Y Liao W-k Choudhary A A fast high utility itemsets mining algorithm Proceedings of the 1st international workshop on Utility-based data mining 2005 1089839 Chicago, Illinois: ACM 90 99

[B15] Tseng VS Wu C-W Shie B-E Yu PS UP-Growth: an efficient algorithm for high utility itemset mining Proceedings of the 16th ACM SIGKDD international conference on Knowledge discovery and data mining 2010 1835839 Washington, DC, USA: ACM 253 262

[B16] Yao H Hamilton HJ Geng L A unified framework for utility-based measures for mining itemsets Second International Workshop on Utility-Based Data Mining 2006 Philadelphia, PA, USA: ACM 28 37

[B17] Yen S-J Lee Y-S Song IY, Eder J, Nguyen TM Mining high utility quantitative association rules DaWaK 2007 Springer 283 292 conf/dawak/YenL07

[B18] Hong T-P Lee C-H Wang S-L Mining high average-utility itemsets Proceedings of the 2009 IEEE international conference on Systems, Man and Cybernetics 2009 1732136 San Antonio, TX, USA: IEEE Press 2526 2530

[B19] Lin C-W Hong T-P Lu W-H Efficiently mining high average utility itemsets with a tree structure Proceedings of the Second international conference on Intelligent information and database systems: Part I 2010 1894770 Hue, Vietnam: Springer-Verlag 131 139

[B20] Stark C Breitkreutz BJ Reguly T Boucher L Breitkreutz A Tyers M BioGRID: a general repository for interaction datasets Nucleic Acids Res 2006 34 D535 D539 Database issue 10.1093/nar/gkj109 16381927 PMC1347471

[B21] Ogata H Goto S Sato K Fujibuchi W Bono H Kanehisa M KEGG: Kyoto Encyclopedia of Genes and Genomes Nucleic Acids Res 1999 27 1 29 34 10.1093/nar/27.1.29 9847135 PMC148090

[B22] Yoshizuka N Yoshizuka-Chadani Y Krishnan V Zeichner SL Human immunodeficiency virus type 1 Vpr-dependent cell cycle arrest through a mitogen-activated protein kinase signal transduction pathway J Virol 2005 79 17 11366 11381 10.1128/JVI.79.17.11366-11381.2005 16103188 PMC1193619

[B23] Sciuto AM Phillips CS Orzolek LD Hege AI Moran TS Dillman JF 3rd Genomic analysis of murine pulmonary tissue following carbonyl chloride inhalation Chem Res Toxicol 2005 18 11 1654 1660 10.1021/tx050126f 16300373

[B24] Zhao Y Lu S Wu L Chai G Wang H Chen Y Sun J Yu Y Zhou W Zheng Q Acetylation of p53 at lysine 373/382 by the histone deacetylase inhibitor depsipeptide induces expression of p21(Waf1/Cip1) Mol Cell Biol 2006 26 7 2782 2790 10.1128/MCB.26.7.2782-2790.2006 16537920 PMC1430330

[B25] Pratt MA Niu MY Bcl-2 controls caspase activation following a p53-dependent cyclin D1-induced death signal J Biol Chem 2003 278 16 14219 14229 10.1074/jbc.M209650200 12480939

[B26] Perego P Giarola M Righetti SC Supino R Caserini C Delia D Pierotti MA Miyashita T Reed JC Zunino F Association between cisplatin resistance and mutation of p53 gene and reduced bax expression in ovarian carcinoma cell systems Cancer Res 1996 56 3 556 562 8564971

[B27] Menendez D Inga A Resnick MA The biological impact of the human master regulator p53 can be altered by mutations that change the spectrum and expression of its target genes Mol Cell Biol 2006 26 6 2297 2308 10.1128/MCB.26.6.2297-2308.2006 16508005 PMC1430278

[B28] Ashburner M Ball CA Blake JA Botstein D Butler H Cherry JM Davis AP Dolinski K Dwight SS Eppig JT Gene ontology: tool for the unification of biology The Gene Ontology Consortium. Nature genetics 2000 25 1 25 29 10.1038/75556 PMC3037419 10802651

[B29] Dennis G Jr Sherman BT Hosack DA Yang J Gao W Lane HC Lempicki RA DAVID: Database for Annotation, Visualization, and Integrated Discovery Genome Biol 2003 4 5 P3 10.1186/gb-2003-4-5-p3 12734009 PMC193660

[B30] Febbo PG Kantoff PW Noise and bias in microarray analysis of tumor specimens Journal of clinical oncology: official journal of the American Society of Clinical Oncology 2006 24 23 3719 3721 10.1200/JCO.2006.06.7942 16822841

