# Supplementary material for: Mining differential top-k co-expression patterns from time course comparative gene expression datasets
Source: BMC Bioinformatics. 2013 Jul 21;14:230. doi: 10.1186/1471-2105-14-230 (PMC3751367; doi:10.1186/1471-2105-14-230)
Supplement: Additional file 1 — Detailed top-50 impactful itemsets and literature evaluation results. [file 1471-2105-14-230-S1.pdf]

List of Supplementary Tables

Supplementary Table 1. Top-50 impactful itemsets of Wild type dataset in comparison 1 of undifferentiation control method

Supplementary Table 2. Top-50 impactful itemsets of Wild type dataset in comparison 1 with a constant degree

Supplementary Table 3. Top-50 impactful itemsets of Wild type dataset in comparison 1 with various degrees

Supplementary Table 4. Top-50 impactful itemsets of F72A/R73A dataset in comparison 1 of undifferentiation control method

Supplementary Table 5. Top-50 impactful itemsets of F72A/R73A dataset in comparison 1 with a constant degree

Supplementary Table 6. Top-50 impactful itemsets of F72A/R73A dataset in comparison 1with various degrees

Supplementary Table 7. Top-50 impactful itemsets of Wild type dataset in comparison 2 of undifferentiation control method

Supplementary Table 8. Top-50 impactful itemsets of Wild type dataset in comparison 2 with a constant degree

Supplementary Table 9. Top-50 impactful itemsets of Wild type dataset in comparison 2 with various degrees

Supplementary Table 10. Top-50 impactful itemsets of R80A dataset in comparison 2 of undifferentiation control method

Supplementary Table 11. Top-50 impactful itemsets of R80A dataset in comparison 2 with a constant degree

Supplementary Table 12. Top-50 impactful itemsets of R80A dataset in comparison 2 with various degrees

Supplementary Table 13. Top-50 impactful itemsets of Air dataset in comparison 3 of undifferentiation control method

Supplementary Table 14. Top-50 impactful itemsets of Air dataset in comparison 3 with a constant degree

Supplementary Table 15. Top-50 impactful itemsets of Air dataset in comparison 3 with various degrees

Supplementary Table 16. Top-50 impactful itemsets of Phosgene dataset in comparison 3 of undifferentiation control method

Supplementary Table 17. Top-50 impactful itemsets of Phosgene dataset in comparison 3 with a constant degree

Supplementary Table 18. Top-50 impactful itemsets of Phosgene dataset in comparison 3 with various degrees

Supplementary Table 19. Disassembled relations derived from Top-50 impactful itemsets of Wild type dataset in comparison 1 of undifferentiation control method

Supplementary Table 20. Disassembled relations derived from Top-50 impactful itemsets of Wild type dataset in comparison 1 with a constant degree

Supplementary Table 21. Disassembled relations derived from Top-50 impactful itemsets of Wild type dataset in comparison 1 with various degrees

Supplementary Table 22. Disassembled relations derived from Top-50 impactful itemsets of F72A/R73A dataset in comparison 1 of undifferentiation control method

Supplementary Table 23. Disassembled relations derived from Top-50 impactful itemsets of F72A/R73A dataset in comparison 1 with a constant degree

Supplementary Table 24. Disassembled relations derived from Top-50 impactful itemsets of F72A/R73A dataset in comparison 1 with various degrees

Supplementary Table 25. Disassembled relations derived from Top-50 impactful itemsets of Wild type dataset in comparison 2 of undifferentiation control method

Supplementary Table 26. Disassembled relations derived from Top-50 impactful itemsets of Wild type dataset in comparison 2 with a constant degree

Supplementary Table 27. Disassembled relations derived from Top-50 impactful itemsets of Wild type dataset in comparison 2 with various degrees

Supplementary Table 28. Disassembled relations derived from Top-50 impactful itemsets of R80A dataset in comparison 2 of undifferentiation control method

Supplementary Table 29. Disassembled relations derived from Top-50 impactful itemsets of R80A dataset in comparison 2 with a constant degree

Supplementary Table 30. Disassembled relations derived from Top-50 impactful itemsets of R80A dataset in comparison 2 with various degrees

Supplementary Table 31. Disassembled relations derived from Top-50 impactful itemsets of Air dataset in comparison 3 of undifferentiation control method

Supplementary Table 32. Disassembled relations derived from Top-50 impactful itemsets of Air dataset in comparison 3 with a constant degree

Supplementary Table 33. Disassembled relations derived from Top-50 impactful itemsets of Air dataset in comparison 3 with various degrees

Supplementary Table 34. Disassembled relations derived from Top-50 impactful itemsets of Phosgene dataset in comparison 3 of undifferentiation control method

Supplementary Table 35. Disassembled relations derived from Top-50 impactful itemsets of Phosgene dataset in comparison 3 with a constant degree

Supplementary Table 36. Disassembled relations derived from Top-50 impactful itemsets of Phosgene dataset in comparison 3 with various degrees

Supplementary Table 1. Top-50 impactful itemsets of Wild type dataset in comparison 1 of undifferentiation control method

Ranking	Impactful itemsets
1	i({UBR1(197131,1177372_1)_Up,ELMO1(9844,1177818_1)_Up})=27.00
2	i({UBR1(197131,1177372_1)_Up,AJAP1(55966,1185257_1)_Up})=27.00
3	i({ELMO1(9844,1177818_1)_Up,AJAP1(55966,1185257_1)_Up})=27.00
4	i({UBR1(197131,1177372_1)_Up,CNOT4(4850,1189014_1)_Up})=27.00
5	i({ELMO1(9844,1177818_1)_Up,CNOT4(4850,1189014_1)_Up})=27.00
6	i({AJAP1(55966,1185257_1)_Up,CNOT4(4850,1189014_1)_Up})=27.00
7	i({UBR1(197131,1177372_1)_Up,ELMO1(9844,1177818_1)_Up,AJAP1(55966,1185257_1)_Up})=27.00
8	i({UBR1(197131,1177372_1)_Up,ELMO1(9844,1177818_1)_Up,CNOT4(4850,1189014_1)_Up})=27.00
9	i({UBR1(197131,1177372_1)_Up,AJAP1(55966,1185257_1)_Up,CNOT4(4850,1189014_1)_Up})=27.00
10	i({UBR1(197131,1177372_1)_Up,ELMO1(9844,1177818_1)_Up,AJAP1(55966,1185257_1)_Up,CNOT4(4850,1189014_1)_Up})=27.00
11	i({UBR1(197131,1177372_1)_Up,ELMO1(9844,1177818_1)_Up,AJAP1(55966,1185257_1)_Up,CNOT4(4850,1189014_1)_Up,MLF2(8079,1173707_1)_Down})=26.80
12	i({UBR1(197131,1177372_1)_Up,ELMO1(9844,1177818_1)_Up,AJAP1(55966,1185257_1)_Up,CNOT4(4850,1189014_1)_Up,ITGA8(8516,1174438_1)_Down})=26.80
13	i({UBR1(197131,1177372_1)_Up,ELMO1(9844,1177818_1)_Up,AJAP1(55966,1185257_1)_Up,MLF2(8079,1173707_1)_Down})=26.75
14	i({UBR1(197131,1177372_1)_Up,ELMO1(9844,1177818_1)_Up,CNOT4(4850,1189014_1)_Up,MLF2(8079,1173707_1)_Down})=26.75
15	i({UBR1(197131,1177372_1)_Up,ELMO1(9844,1177818_1)_Up,AJAP1(55966,1185257_1)_Up,ITGA8(8516,1174438_1)_Down})=26.75
16	i({UBR1(197131,1177372_1)_Up,ELMO1(9844,1177818_1)_Up,CNOT4(4850,1189014_1)_Up,ITGA8(8516,1174438_1)_Down})=26.75
17	i({UBR1(197131,1177372_1)_Up,AJAP1(55966,1185257_1)_Up,CNOT4(4850,1189014_1)_Up,MLF2(8079,1173707_1)_Down})=26.75
18	i({UBR1(197131,1177372_1)_Up,AJAP1(55966,1185257_1)_Up,CNOT4(4850,1189014_1)_Up,ITGA8(8516,1174438_1)_Down})=26.75
19	i({UBR1(197131,1177372_1)_Up,ELMO1(9844,1177818_1)_Up,AJAP1(55966,1185257_1)_Up,CNOT4(4850,1189014_1)_Up,MLF2(8079,1173707_1)_Down,ITGA8(8516,1174438_1)_Down})=26.67
20	i({UBR1(197131,1177372_1)_Up,ELMO1(9844,1177818_1)_Up,MLF2(8079,1173707_1)_Down})=26.67
21	i({UBR1(197131,1177372_1)_Up,AJAP1(55966,1185257_1)_Up,MLF2(8079,1173707_1)_Down})=26.67
22	i({UBR1(197131,1177372_1)_Up,CNOT4(4850,1189014_1)_Up,MLF2(8079,1173707_1)_Down})=26.67
23	i({UBR1(197131,1177372_1)_Up,ELMO1(9844,1177818_1)_Up,ITGA8(8516,1174438_1)_Down})=26.67
24	i({UBR1(197131,1177372_1)_Up,AJAP1(55966,1185257_1)_Up,ITGA8(8516,1174438_1)_Down})=26.67
25	i({UBR1(197131,1177372_1)_Up,CNOT4(4850,1189014_1)_Up,ITGA8(8516,1174438_1)_Down})=26.67
26	i({UBR1(197131,1177372_1)_Up,ELMO1(9844,1177818_1)_Up,AJAP1(55966,1185257_1)_Up,MLF2(8079,1173707_1)_Down,ITGA8(8516,1174438_1)_Down})=26.60
27	i({UBR1(197131,1177372_1)_Up,ELMO1(9844,1177818_1)_Up,CCDC111(201973,1179751_1)_Up,AJAP1(55966,1185257_1)_Up,CNOT4(4850,1189014_1)_Up})=26.60
28	i({UBR1(197131,1177372_1)_Up,ELMO1(9844,1177818_1)_Up,AJAP1(55966,1185257_1)_Up,CNOT4(4850,1189014_1)_Up,KLHL8(57563,1173854_1)_Down})=26.60
29	i({UBR1(197131,1177372_1)_Up,ELMO1(9844,1177818_1)_Up,AJAP1(55966,1185257_1)_Up,CNOT4(4850,1189014_1)_Up,SCRN2(90507,1173975_1)_Down})=26.60
30	i({UBR1(197131,1177372_1)_Up,ELMO1(9844,1177818_1)_Up,AJAP1(55966,1185257_1)_Up,CNOT4(4850,1189014_1)_Up,CALML4(91860,1173977_1)_Down})=26.60
31	i({UBR1(197131,1177372_1)_Up,ELMO1(9844,1177818_1)_Up,AJAP1(55966,1185257_1)_Up,CNOT4(4850,1189014_1)_Up,PDXDC1(23042

	,1174090_1)_Down})=26.60
32	i({UBR1(197131,1177372_1)_Up,ELMO1(9844,1177818_1)_Up,PCIF1(63935,1185032_1)_Up,AJAP1(55966,1185257_1)_Up,CNOT4(4850,1189014_1)_Up})=26.60
33	i({UBR1(197131,1177372_1)_Up,ELMO1(9844,1177818_1)_Up,AJAP1(55966,1185257_1)_Up,CNOT4(4850,1189014_1)_Up,TARP(445347,1177766_1)_Down})=26.60
34	i({UBR1(197131,1177372_1)_Up,ELMO1(9844,1177818_1)_Up,CNOT4(4850,1189014_1)_Up,MLF2(8079,1173707_1)_Down,ITGA8(8516,1174438_1)_Down})=26.60
35	i({UBR1(197131,1177372_1)_Up,AJAP1(55966,1185257_1)_Up,CNOT4(4850,1189014_1)_Up,MLF2(8079,1173707_1)_Down,ITGA8(8516,1174438_1)_Down})=26.60
36	i({UBR1(197131,1177372_1)_Up,MLF2(8079,1173707_1)_Down})=26.50
37	i({ELMO1(9844,1177818_1)_Up,MLF2(8079,1173707_1)_Down})=26.50
38	i({AJAP1(55966,1185257_1)_Up,MLF2(8079,1173707_1)_Down})=26.50
39	i({CNOT4(4850,1189014_1)_Up,MLF2(8079,1173707_1)_Down})=26.50
40	i({UBR1(197131,1177372_1)_Up,ITGA8(8516,1174438_1)_Down})=26.50
41	i({ELMO1(9844,1177818_1)_Up,ITGA8(8516,1174438_1)_Down})=26.50
42	i({AJAP1(55966,1185257_1)_Up,ITGA8(8516,1174438_1)_Down})=26.50
43	i({CNOT4(4850,1189014_1)_Up,ITGA8(8516,1174438_1)_Down})=26.50
44	i({UBR1(197131,1177372_1)_Up,ELMO1(9844,1177818_1)_Up,MLF2(8079,1173707_1)_Down,ITGA8(8516,1174438_1)_Down})=26.50
45	i({UBR1(197131,1177372_1)_Up,ELMO1(9844,1177818_1)_Up,CCDC111(201973,1179751_1)_Up,AJAP1(55966,1185257_1)_Up})=26.50
46	i({UBR1(197131,1177372_1)_Up,ELMO1(9844,1177818_1)_Up,CCDC111(201973,1179751_1)_Up,CNOT4(4850,1189014_1)_Up})=26.50
47	i({UBR1(197131,1177372_1)_Up,ELMO1(9844,1177818_1)_Up,AJAP1(55966,1185257_1)_Up,KLHL8(57563,1173854_1)_Down})=26.50
48	i({UBR1(197131,1177372_1)_Up,ELMO1(9844,1177818_1)_Up,CNOT4(4850,1189014_1)_Up,KLHL8(57563,1173854_1)_Down})=26.50
49	i({UBR1(197131,1177372_1)_Up,ELMO1(9844,1177818_1)_Up,AJAP1(55966,1185257_1)_Up,SCRN2(90507,1173975_1)_Down})=26.50
50	i({UBR1(197131,1177372_1)_Up,ELMO1(9844,1177818_1)_Up,CNOT4(4850,1189014_1)_Up,SCRN2(90507,1173975_1)_Down})=26.50

Supplementary Table 2. Top-50 impactful itemsets of Wild type dataset in comparison 1 with a constant degree

Ranking	Impactful itemsets
1	i({STRAP(11171,1174318_1)_Up,PTK2B(2185,1176416_1)_Up})=17.00
2	i({STRAP(11171,1174318_1)_Up,PTK2B(2185,1176416_1)_Up,HSPA1A(3303,1195261_1)_Up})=16.67
3	i({STRAP(11171,1174318_1)_Up,PTK2B(2185,1176416_1)_Up,HSPA1A(3303,1195285_1)_Up})=16.67
4	i({STRAP(11171,1174318_1)_Up,PTK2B(2185,1176416_1)_Up,HSPA1A(3303,1195309_1)_Up})=16.67
5	i({STRAP(11171,1174318_1)_Up,PTK2B(2185,1176416_1)_Up,HSPA1A(3303,1195237_1)_Up})=16.67
6	i({STRAP(11171,1174318_1)_Up,PTK2B(2185,1176416_1)_Up,MYC(4609,1180176_1)_Up})=16.67
7	i({STRAP(11171,1174318_1)_Up,MYC(4609,1180176_1)_Up})=16.50
8	i({PTK2B(2185,1176416_1)_Up,MYC(4609,1180176_1)_Up})=16.50
9	i({STRAP(11171,1174318_1)_Up,HSPA1A(3303,1195261_1)_Up})=16.50
10	i({PTK2B(2185,1176416_1)_Up,HSPA1A(3303,1195261_1)_Up})=16.50
11	i({STRAP(11171,1174318_1)_Up,HSPA1A(3303,1195285_1)_Up})=16.50
12	i({PTK2B(2185,1176416_1)_Up,HSPA1A(3303,1195285_1)_Up})=16.50
13	i({STRAP(11171,1174318_1)_Up,HSPA1A(3303,1195309_1)_Up})=16.50
14	i({PTK2B(2185,1176416_1)_Up,HSPA1A(3303,1195309_1)_Up})=16.50
15	i({STRAP(11171,1174318_1)_Up,PTK2B(2185,1176416_1)_Up,MYC(4609,1180176_1)_Up,HSPA1A(3303,1195261_1)_Up})=16.50
16	i({STRAP(11171,1174318_1)_Up,PTK2B(2185,1176416_1)_Up,MYC(4609,1180176_1)_Up,HSPA1A(3303,1195285_1)_Up})=16.50
17	i({STRAP(11171,1174318_1)_Up,PTK2B(2185,1176416_1)_Up,MYC(4609,1180176_1)_Up,HSPA1A(3303,1195309_1)_Up})=16.50
18	i({STRAP(11171,1174318_1)_Up,PTK2B(2185,1176416_1)_Up,HSPA1A(3303,1195261_1)_Up,HSPA1A(3303,1195285_1)_Up})=16.50
19	i({STRAP(11171,1174318_1)_Up,PTK2B(2185,1176416_1)_Up,HSPA1A(3303,1195261_1)_Up,HSPA1A(3303,1195309_1)_Up})=16.50
20	i({STRAP(11171,1174318_1)_Up,PTK2B(2185,1176416_1)_Up,HSPA1A(3303,1195285_1)_Up,HSPA1A(3303,1195309_1)_Up})=16.50
21	i({STRAP(11171,1174318_1)_Up,PTK2B(2185,1176416_1)_Up,HSPA1A(3303,1195237_1)_Up,HSPA1A(3303,1195261_1)_Up})=16.50
22	i({STRAP(11171,1174318_1)_Up,PTK2B(2185,1176416_1)_Up,HSPA1A(3303,1195237_1)_Up,HSPA1A(3303,1195285_1)_Up})=16.50
23	i({STRAP(11171,1174318_1)_Up,PTK2B(2185,1176416_1)_Up,HSPA1A(3303,1195237_1)_Up,HSPA1A(3303,1195309_1)_Up})=16.50
24	i({STRAP(11171,1174318_1)_Up,PTK2B(2185,1176416_1)_Up,MYC(4609,1180176_1)_Up,HSPA1A(3303,1195237_1)_Up})=16.50
25	i({STRAP(11171,1174318_1)_Up,PTK2B(2185,1176416_1)_Up,HSPA1A(3303,1195237_1)_Up,HSPA1A(3303,1195261_1)_Up,HSPA1A(3303, 1195285_1)_Up})=16.40
26	i({STRAP(11171,1174318_1)_Up,PTK2B(2185,1176416_1)_Up,HSPA1A(3303,1195237_1)_Up,HSPA1A(3303,1195261_1)_Up,HSPA1A(3303, 1195309_1)_Up})=16.40
27	i({STRAP(11171,1174318_1)_Up,PTK2B(2185,1176416_1)_Up,HSPA1A(3303,1195237_1)_Up,HSPA1A(3303,1195285_1)_Up,HSPA1A(3303, 1195309_1)_Up})=16.40
28	i({STRAP(11171,1174318_1)_Up,PTK2B(2185,1176416_1)_Up,MYC(4609,1180176_1)_Up,HSPA1A(3303,1195261_1)_Up,HSPA1A(3303,119 5285_1)_Up})=16.40
29	i({STRAP(11171,1174318_1)_Up,PTK2B(2185,1176416_1)_Up,MYC(4609,1180176_1)_Up,HSPA1A(3303,1195261_1)_Up,HSPA1A(3303,119 5309_1)_Up})=16.40
30	i({STRAP(11171,1174318_1)_Up,PTK2B(2185,1176416_1)_Up,MYC(4609,1180176_1)_Up,HSPA1A(3303,1195285_1)_Up,HSPA1A(3303,119 5309_1)_Up})=16.40
31	i({STRAP(11171,1174318_1)_Up,PTK2B(2185,1176416_1)_Up,MYC(4609,1180176_1)_Up,HSPA1A(3303,1195237_1)_Up,HSPA1A(3303,119 5261_1)_Up})=16.40
32	i({STRAP(11171,1174318_1)_Up,PTK2B(2185,1176416_1)_Up,MYC(4609,1180176_1)_Up,HSPA1A(3303,1195237_1)_Up,HSPA1A(3303,119

	5285_1)_Up})=16.40
33	i({STRAP(11171,1174318_1)_Up,PTK2B(2185,1176416_1)_Up,MYC(4609,1180176_1)_Up,HSPA1A(3303,1195237_1)_Up,HSPA1A(3303,1195309_1)_Up})=16.40
34	i({PTK2B(2185,1176416_1)_Up,MYC(4609,1180176_1)_Up,HSPA1A(3303,1195261_1)_Up})=16.33
35	i({PTK2B(2185,1176416_1)_Up,MYC(4609,1180176_1)_Up,HSPA1A(3303,1195285_1)_Up})=16.33
36	i({PTK2B(2185,1176416_1)_Up,MYC(4609,1180176_1)_Up,HSPA1A(3303,1195309_1)_Up})=16.33
37	i({PTK2B(2185,1176416_1)_Up,MYC(4609,1180176_1)_Up,HSPA1A(3303,1195237_1)_Up})=16.33
38	i({STRAP(11171,1174318_1)_Up,MYC(4609,1180176_1)_Up,HSPA1A(3303,1195261_1)_Up})=16.33
39	i({STRAP(11171,1174318_1)_Up,MYC(4609,1180176_1)_Up,HSPA1A(3303,1195285_1)_Up})=16.33
40	i({STRAP(11171,1174318_1)_Up,HSPA1A(3303,1195261_1)_Up,HSPA1A(3303,1195285_1)_Up})=16.33
41	i({STRAP(11171,1174318_1)_Up,MYC(4609,1180176_1)_Up,HSPA1A(3303,1195309_1)_Up})=16.33
42	i({STRAP(11171,1174318_1)_Up,HSPA1A(3303,1195261_1)_Up,HSPA1A(3303,1195309_1)_Up})=16.33
43	i({STRAP(11171,1174318_1)_Up,HSPA1A(3303,1195285_1)_Up,HSPA1A(3303,1195309_1)_Up})=16.33
44	i({STRAP(11171,1174318_1)_Up,PTK2B(2185,1176416_1)_Up,HSPA1A(3303,1195213_1)_Up})=16.33
45	i({STRAP(11171,1174318_1)_Up,PTK2B(2185,1176416_1)_Up,HSPA1A(3303,1195045_1)_Up})=16.33
46	i({STRAP(11171,1174318_1)_Up,PTK2B(2185,1176416_1)_Up,HSPA1A(3303,1195333_1)_Up})=16.33
47	i({PTK2B(2185,1176416_1)_Up,HSPA1A(3303,1195261_1)_Up,HSPA1A(3303,1195285_1)_Up})=16.33
48	i({PTK2B(2185,1176416_1)_Up,HSPA1A(3303,1195261_1)_Up,HSPA1A(3303,1195309_1)_Up})=16.33
49	i({PTK2B(2185,1176416_1)_Up,HSPA1A(3303,1195285_1)_Up,HSPA1A(3303,1195309_1)_Up})=16.33
50	i({STRAP(11171,1174318_1)_Up,PTK2B(2185,1176416_1)_Up,SPI1(6688,1180830_1)_Down})=16.33

Supplementary Table 3. Top-50 impactful itemsets of Wild type dataset in comparison 1 with various degrees

Ranking	Impactful itemsets
1	i({MYC(4609,1180176_1)_Up,CREBBP(1387,1176823_1)_Down})=79.50
2	i({CREBBP(1387,1176823_1)_Down,SPI1(6688,1180830_1)_Down})=61.50
3	i({MYC(4609,1180176_1)_Up,CREBBP(1387,1176823_1)_Down,SPI1(6688,1180830_1)_Down})=61.00
4	i({STRAP(11171,1174318_1)_Up,CREBBP(1387,1176823_1)_Down})=60.50
5	i({STRAP(11171,1174318_1)_Up,MYC(4609,1180176_1)_Up,CREBBP(1387,1176823_1)_Down})=60.33
6	i({MYC(4609,1180176_1)_Up,CREBBP(1387,1176823_1)_Down,TP53(7157,1193761_1)_Down})=59.67
7	i({TP53(7157,1173966_1)_Up,MYC(4609,1180176_1)_Up,CREBBP(1387,1176823_1)_Down})=59.67
8	i({MYC(4609,1180176_1)_Up,GLI1(2735,1174154_1)_Down,CREBBP(1387,1176823_1)_Down})=59.00
9	i({MYC(4609,1180176_1)_Up,CREBBP(1387,1176823_1)_Down,PDX1(3651,1177720_1)_Down})=58.33
10	i({MYC(4609,1180176_1)_Up,GCK(2645,1173818_1)_Down,CREBBP(1387,1176823_1)_Down})=57.67
11	i({MYC(4609,1180176_1)_Up,CREBBP(1387,1176823_1)_Down,NEUROD1(4760,1179370_1)_Down})=57.67
12	i({MYC(4609,1180176_1)_Up,SP4(6671,1174213_1)_Down,CREBBP(1387,1176823_1)_Down})=57.67
13	i({PTK2B(2185,1176416_1)_Up,MYC(4609,1180176_1)_Up,CREBBP(1387,1176823_1)_Down})=57.33
14	i({MYC(4609,1180176_1)_Up,HSPA1A(3303,1195309_1)_Up,CREBBP(1387,1176823_1)_Down})=57.33
15	i({MYC(4609,1180176_1)_Up,HSPA1A(3303,1195261_1)_Up,CREBBP(1387,1176823_1)_Down})=57.33
16	i({MYC(4609,1180176_1)_Up,HSPA1A(3303,1195285_1)_Up,CREBBP(1387,1176823_1)_Down})=57.33
17	i({STRAP(11171,1174318_1)_Up,MYC(4609,1180176_1)_Up})=57.00
18	i({MYC(4609,1180176_1)_Up,HSPA1A(3303,1195213_1)_Up,CREBBP(1387,1176823_1)_Down})=57.00
19	i({MYC(4609,1180176_1)_Up,HSPA1A(3303,1195045_1)_Up,CREBBP(1387,1176823_1)_Down})=57.00
20	i({MYC(4609,1180176_1)_Up,HSPA1A(3303,1195333_1)_Up,CREBBP(1387,1176823_1)_Down})=57.00
21	i({MYC(4609,1180176_1)_Up,HSPA1A(3303,1195381_1)_Up,CREBBP(1387,1176823_1)_Down})=57.00
22	i({MYC(4609,1180176_1)_Up,EGR1(1958,1192846_1)_Up,CREBBP(1387,1176823_1)_Down})=57.00
23	i({MYC(4609,1180176_1)_Up,CREBBP(1387,1176823_1)_Down,MAPK1(5594,1192793_1)_Down})=57.00
24	i({MYC(4609,1180176_1)_Up,STAT3(6774,1192670_1)_Up,CREBBP(1387,1176823_1)_Down})=57.00
25	i({HSPA1A(3303,1175226_1)_Up,MYC(4609,1180176_1)_Up,CREBBP(1387,1176823_1)_Down})=56.67
26	i({MYC(4609,1180176_1)_Up,CREBBP(1387,1176823_1)_Down,SPRY3(10251,1183715_1)_Down})=56.67
27	i({MYC(4609,1180176_1)_Up,SCNN1A(6337,1174175_1)_Down,CREBBP(1387,1176823_1)_Down})=56.33
28	i({MYC(4609,1180176_1)_Up,CREBBP(1387,1176823_1)_Down,HNF4A(3172,1178626_1)_Down})=56.33
29	i({MYC(4609,1180176_1)_Up,CREBBP(1387,1176823_1)_Down,TNF(7124,1189430_1)_Down})=56.33
30	i({PTK2B(2185,1176416_1)_Up,CREBBP(1387,1176823_1)_Down})=56.00
31	i({HSPA1A(3303,1195261_1)_Up,CREBBP(1387,1176823_1)_Down})=56.00
32	i({HSPA1A(3303,1195285_1)_Up,CREBBP(1387,1176823_1)_Down})=56.00
33	i({HSPA1A(3303,1195309_1)_Up,CREBBP(1387,1176823_1)_Down})=56.00
34	i({MYC(4609,1180176_1)_Up,CREBBP(1387,1176823_1)_Down,NCOA2(10499,1177421_1)_Down})=56.00
35	i({MYC(4609,1180176_1)_Up,CREBBP(1387,1176823_1)_Down,NEUROD1(4760,1194241_1)_Down})=55.67
36	i({MYC(4609,1180176_1)_Up,IFNG(3458,1173720_1)_Down,CREBBP(1387,1176823_1)_Down})=55.67
37	i({MYC(4609,1180176_1)_Up,CREBBP(1387,1176823_1)_Down,CSF2RA(1438,1182161_1)_Down})=55.67
38	i({MYC(4609,1180176_1)_Up,IL8(3576,1173646_1)_Down,CREBBP(1387,1176823_1)_Down})=55.67
39	i({MYC(4609,1180176_1)_Up,PDX1(3651,1189704_1)_Up,CREBBP(1387,1176823_1)_Down})=55.67

40	i({HSPA1A(3303,1195213_1)_Up,CREBBP(1387,1176823_1)_Down})=55.50
41	i({HSPA1A(3303,1195045_1)_Up,CREBBP(1387,1176823_1)_Down})=55.50
42	i({HSPA1A(3303,1195333_1)_Up,CREBBP(1387,1176823_1)_Down})=55.50
43	i({HSPA1A(3303,1195381_1)_Up,CREBBP(1387,1176823_1)_Down})=55.50
44	i({MYC(4609,1180176_1)_Up,CREBBP(1387,1176823_1)_Down,IFNB1(3456,1184164_1)_Down})=55.33
45	i({MYC(4609,1180176_1)_Up,CREBBP(1387,1176823_1)_Down,CXCL11(6373,1184884_1)_Down})=55.33
46	i({MYC(4609,1180176_1)_Up,CREBBP(1387,1176823_1)_Down,SOCS4(122809,1194141_1)_Down})=55.33
47	i({SHISA5(51246,1176904_1)_Up,MYC(4609,1180176_1)_Up,CREBBP(1387,1176823_1)_Down})=55.33
48	i({MYC(4609,1180176_1)_Up,LTBR(4055,1173781_1)_Down,CREBBP(1387,1176823_1)_Down})=55.33
49	i({MYC(4609,1180176_1)_Up,CREBBP(1387,1176823_1)_Down,PEX11A(8800,1177592_1)_Down})=55.33
50	i({MYC(4609,1180176_1)_Up,BAI1(575,1188280_1)_Up,CREBBP(1387,1176823_1)_Down})=55.00

Supplementary Table 4. Top-50 impactful itemsets of F72A/R73A dataset in comparison 1 of undifferentiation control method

Ranking	Impactful itemsets
1	i({RUSC2(9853,1193203_1)_Up,ITPK1(3705,1182315_1)_Down})=20.50
2	i({PPIF(10105,1190780_1)_Up,RUSC2(9853,1193203_1)_Up,ITPK1(3705,1182315_1)_Down})=18.67
3	i({RUSC2(9853,1193203_1)_Up,MINA(84864,1179265_1)_Down,ITPK1(3705,1182315_1)_Down})=18.67
4	i({LMCD1(29995,1188362_1)_Up,RUSC2(9853,1193203_1)_Up,ITPK1(3705,1182315_1)_Down})=18.67
5	i({RUSC2(9853,1193203_1)_Up,ITPK1(3705,1182315_1)_Down,KSR1(8844,1189580_1)_Down})=18.67
6	i({RMND5B(64777,1183974_1)_Up,RUSC2(9853,1193203_1)_Up,ITPK1(3705,1182315_1)_Down})=18.67
7	i({RUSC2(9853,1193203_1)_Up,RBMXL2(27288,1175128_1)_Down,ITPK1(3705,1182315_1)_Down})=18.67
8	i({PLXNB1(5364,1179050_1)_Up,RUSC2(9853,1193203_1)_Up,ITPK1(3705,1182315_1)_Down})=18.33
9	i({RUSC2(9853,1193203_1)_Up,ELOVL1(64834,1173605_1)_Down,ITPK1(3705,1182315_1)_Down})=18.33
10	i({RUSC2(9853,1193203_1)_Up,ITPK1(3705,1182315_1)_Down,DSG1(1828,1184540_1)_Down})=18.33
11	i({C11orf54(28970,1189207_1)_Up,RUSC2(9853,1193203_1)_Up,ITPK1(3705,1182315_1)_Down})=18.00
12	i({RUSC2(9853,1193203_1)_Up,MINA(84864,1179265_1)_Down})=18.00
13	i({LMCD1(29995,1188362_1)_Up,RUSC2(9853,1193203_1)_Up})=18.00
14	i({RUSC2(9853,1193203_1)_Up,SH3PXD2A(9644,1180753_1)_Down,ITPK1(3705,1182315_1)_Down})=18.00
15	i({RUSC2(9853,1193203_1)_Up,ITPK1(3705,1182315_1)_Down,PML(5371,1185459_1)_Down})=18.00
16	i({RUSC2(9853,1193203_1)_Up,C3orf18(51161,1177952_1)_Down,ITPK1(3705,1182315_1)_Down})=18.00
17	i({RUSC2(9853,1193203_1)_Up,ITPK1(3705,1182315_1)_Down,ACTB(60,1190443_1)_Down})=18.00
18	i({LMCD1(29995,1188362_1)_Up,RUSC2(9853,1193203_1)_Up,MINA(84864,1179265_1)_Down,ITPK1(3705,1182315_1)_Down})=17.75
19	i({RUSC2(9853,1193203_1)_Up,MINA(84864,1179265_1)_Down,SH3PXD2A(9644,1180753_1)_Down,ITPK1(3705,1182315_1)_Down})=17.75
20	i({LMCD1(29995,1188362_1)_Up,RUSC2(9853,1193203_1)_Up,SH3PXD2A(9644,1180753_1)_Down,ITPK1(3705,1182315_1)_Down})=17.75
21	i({RUSC2(9853,1193203_1)_Up,MINA(84864,1179265_1)_Down,ITPK1(3705,1182315_1)_Down,KSR1(8844,1189580_1)_Down})=17.75
22	i({LMCD1(29995,1188362_1)_Up,RUSC2(9853,1193203_1)_Up,ITPK1(3705,1182315_1)_Down,KSR1(8844,1189580_1)_Down})=17.75
23	i({RMND5B(64777,1183974_1)_Up,RUSC2(9853,1193203_1)_Up,MINA(84864,1179265_1)_Down,ITPK1(3705,1182315_1)_Down})=17.75
24	i({RMND5B(64777,1183974_1)_Up,LMCD1(29995,1188362_1)_Up,RUSC2(9853,1193203_1)_Up,ITPK1(3705,1182315_1)_Down})=17.75
25	i({RUSC2(9853,1193203_1)_Up,RBMXL2(27288,1175128_1)_Down,MINA(84864,1179265_1)_Down,ITPK1(3705,1182315_1)_Down})=17.75
26	i({LMCD1(29995,1188362_1)_Up,RUSC2(9853,1193203_1)_Up,RBMXL2(27288,1175128_1)_Down,ITPK1(3705,1182315_1)_Down})=17.75
27	i({DNASE1L3(1776,1192687_1)_Up,RUSC2(9853,1193203_1)_Up,MINA(84864,1179265_1)_Down,ITPK1(3705,1182315_1)_Down})=17.75
28	i({LMCD1(29995,1188362_1)_Up,DNASE1L3(1776,1192687_1)_Up,RUSC2(9853,1193203_1)_Up,ITPK1(3705,1182315_1)_Down})=17.75
29	i({PPIF(10105,1190780_1)_Up,RUSC2(9853,1193203_1)_Up,MINA(84864,1179265_1)_Down,ITPK1(3705,1182315_1)_Down})=17.75
30	i({LMCD1(29995,1188362_1)_Up,PPIF(10105,1190780_1)_Up,RUSC2(9853,1193203_1)_Up,ITPK1(3705,1182315_1)_Down})=17.75
31	i({RUSC2(9853,1193203_1)_Up,ITPK1(3705,1182315_1)_Down,CRHR1(1394,1184093_1)_Down})=17.67
32	i({RUSC2(9853,1193203_1)_Up,ITPK1(3705,1182315_1)_Down,HELLS(3070,1189615_1)_Down})=17.67
33	i({RUSC2(9853,1193203_1)_Up,KCTD17(79734,1181328_1)_Down,ITPK1(3705,1182315_1)_Down})=17.67
34	i({RUSC2(9853,1193203_1)_Up,ITPK1(3705,1182315_1)_Down,LOC644656(644656,1192533_1)_Down})=17.67
35	i({RUSC2(9853,1193203_1)_Up,KIF20A(10112,1178693_1)_Down,ITPK1(3705,1182315_1)_Down})=17.67
36	i({RUSC2(9853,1193203_1)_Up,ITPK1(3705,1182315_1)_Down,C1QBP(708,1192571_1)_Down})=17.67
37	i({RUSC2(9853,1193203_1)_Up,ITPK1(3705,1182315_1)_Down,PLCG2(5336,1186648_1)_Down})=17.67
38	i({RUSC2(9853,1193203_1)_Up,TCEB3B(51224,1177124_1)_Down,ITPK1(3705,1182315_1)_Down})=17.67
39	i({RUSC2(9853,1193203_1)_Up,ITPK1(3705,1182315_1)_Down,GPR157(80045,1184801_1)_Down})=17.67

40	i({RUSC2(9853,1193203_1)_Up,SH3PXD2A(9644,1180753_1)_Down})=17.50
41	i({MINA(84864,1179265_1)_Down,ITPK1(3705,1182315_1)_Down})=17.50
42	i({LMCD1(29995,1188362_1)_Up,ITPK1(3705,1182315_1)_Down})=17.50
43	i({RUSC2(9853,1193203_1)_Up,MAGEA1(4100,1178686_1)_Down,MINA(84864,1179265_1)_Down,ITPK1(3705,1182315_1)_Down})=17.50
44	i({LMCD1(29995,1188362_1)_Up,RUSC2(9853,1193203_1)_Up,MAGEA1(4100,1178686_1)_Down,ITPK1(3705,1182315_1)_Down})=17.50
45	i({RUSC2(9853,1193203_1)_Up,MINA(84864,1179265_1)_Down,ITPK1(3705,1182315_1)_Down,MFSD5(84975,1183963_1)_Down})=17.50
46	i({LMCD1(29995,1188362_1)_Up,RUSC2(9853,1193203_1)_Up,ITPK1(3705,1182315_1)_Down,MFSD5(84975,1183963_1)_Down})=17.50
47	i({PLXNB1(5364,1179050_1)_Up,RUSC2(9853,1193203_1)_Up,MINA(84864,1179265_1)_Down,ITPK1(3705,1182315_1)_Down})=17.50
48	i({PLXNB1(5364,1179050_1)_Up,LMCD1(29995,1188362_1)_Up,RUSC2(9853,1193203_1)_Up,ITPK1(3705,1182315_1)_Down})=17.50
49	i({RUSC2(9853,1193203_1)_Up,ELOVL1(64834,1173605_1)_Down,MINA(84864,1179265_1)_Down,ITPK1(3705,1182315_1)_Down})=17.50
50	i({LMCD1(29995,1188362_1)_Up,RUSC2(9853,1193203_1)_Up,ELOVL1(64834,1173605_1)_Down,ITPK1(3705,1182315_1)_Down})=17.50

Supplementary Table 5. Top-50 impactful itemsets of F72A/R73A dataset in comparison 1 with a constant degree

Ranking	Impactful itemsets
1	i({CRMP1(1400,1187700_1)_Up,KAT2B(8850,1188483_1)_Down})=9.50
2	i({KAT2B(8850,1188483_1)_Down,CTBP1(1487,1193697_1)_Down})=9.00
3	i({CRMP1(1400,1187700_1)_Up,KAT2B(8850,1188483_1)_Down,CTBP1(1487,1193697_1)_Down})=8.67
4	i({CRMP1(1400,1187700_1)_Up,CTBP1(1487,1193697_1)_Down})=8.50
5	i({KAT2B(8850,1188483_1)_Down,CCL2(6347,1191856_1)_Down})=8.50
6	i({KAT2B(8850,1188483_1)_Down,CCL2(6347,1191856_1)_Down,CTBP1(1487,1193697_1)_Down})=8.33
7	i({CRMP1(1400,1187700_1)_Up,NCOA1(8648,1179457_1)_Down,KAT2B(8850,1188483_1)_Down})=8.33
8	i({EPS15(2060,1191958_1)_Up,CTBP1(1487,1193697_1)_Down})=8.00
9	i({CTBP1(1487,1193697_1)_Down,SESN3(143686,1194275_1)_Down})=8.00
10	i({CRMP1(1400,1187700_1)_Up,EPS15(2060,1191958_1)_Up,KAT2B(8850,1188483_1)_Down})=8.00
11	i({CRMP1(1400,1187700_1)_Up,FIGF(2277,1175508_1)_Down,KAT2B(8850,1188483_1)_Down})=8.00
12	i({FIGF(2277,1175508_1)_Down,KAT2B(8850,1188483_1)_Down,CTBP1(1487,1193697_1)_Down})=8.00
13	i({FIGF(2277,1175508_1)_Down,KAT2B(8850,1188483_1)_Down,CCL2(6347,1191856_1)_Down})=8.00
14	i({CRMP1(1400,1187700_1)_Up,MAP2K6(5608,1185773_1)_Down,KAT2B(8850,1188483_1)_Down})=8.00
15	i({CRMP1(1400,1187700_1)_Up,NOTCH3(4854,1175161_1)_Down,KAT2B(8850,1188483_1)_Down})=8.00
16	i({EPS15(2060,1191958_1)_Up,FIGF(2277,1175508_1)_Down,CTBP1(1487,1193697_1)_Down})=7.67
17	i({NCOA1(8648,1179457_1)_Down,KAT2B(8850,1188483_1)_Down,CTBP1(1487,1193697_1)_Down})=7.67
18	i({CRMP1(1400,1187700_1)_Up,KAT2B(8850,1188483_1)_Down,THBS1(7057,1189656_1)_Down})=7.67
19	i({NCOA1(8648,1179457_1)_Down,KAT2B(8850,1188483_1)_Down,CCL2(6347,1191856_1)_Down})=7.67
20	i({CRMP1(1400,1187700_1)_Up,JUP(3728,1174090_1)_Down,KAT2B(8850,1188483_1)_Down})=7.67
21	i({U2AF1(7307,1178903_1)_Up,CRMP1(1400,1187700_1)_Up,KAT2B(8850,1188483_1)_Down})=7.67
22	i({CRMP1(1400,1187700_1)_Up,CDKN2B(1030,1179354_1)_Down,KAT2B(8850,1188483_1)_Down})=7.67
23	i({CRMP1(1400,1187700_1)_Up,KAT2B(8850,1188483_1)_Down,SESN3(143686,1194275_1)_Down})=7.67
24	i({CRMP1(1400,1187700_1)_Up,GADD45G(10912,1175328_1)_Down,KAT2B(8850,1188483_1)_Down})=7.67
25	i({CCL2(6347,1191856_1)_Down,CTBP1(1487,1193697_1)_Down})=7.50
26	i({NOTCH3(4854,1175161_1)_Down,KAT2B(8850,1188483_1)_Down,CCL2(6347,1191856_1)_Down})=7.33
27	i({U2AF1(7307,1178903_1)_Up,KAT2B(8850,1188483_1)_Down,CTBP1(1487,1193697_1)_Down})=7.33
28	i({CRMP1(1400,1187700_1)_Up,CTBP1(1487,1193697_1)_Down,SESN3(143686,1194275_1)_Down})=7.33
29	i({CRMP1(1400,1187700_1)_Up,FIGF(2277,1175508_1)_Down,CTBP1(1487,1193697_1)_Down})=7.33
30	i({CRMP1(1400,1187700_1)_Up,MAP2K1(5604,1174285_1)_Down,KAT2B(8850,1188483_1)_Down})=7.33
31	i({CRMP1(1400,1187700_1)_Up,SERPINE1(5054,1180445_1)_Down,KAT2B(8850,1188483_1)_Down})=7.33
32	i({EPS15(2060,1191958_1)_Up,KAT2B(8850,1188483_1)_Down,CCL2(6347,1191856_1)_Down})=7.33
33	i({BIRC2(329,1183078_1)_Up,CRMP1(1400,1187700_1)_Up,KAT2B(8850,1188483_1)_Down})=7.33
34	i({JUP(3728,1174090_1)_Down,KAT2B(8850,1188483_1)_Down,CTBP1(1487,1193697_1)_Down})=7.33
35	i({CRMP1(1400,1187700_1)_Up,SIAH1(6477,1188058_1)_Up,KAT2B(8850,1188483_1)_Down})=7.33
36	i({SIAH1(6477,1188058_1)_Up,KAT2B(8850,1188483_1)_Down,CTBP1(1487,1193697_1)_Down})=7.33
37	i({SPRY3(10251,1183715_1)_Up,KAT2B(8850,1188483_1)_Down,CTBP1(1487,1193697_1)_Down})=7.33
38	i({KAT2B(8850,1188483_1)_Down,THBS1(7057,1189656_1)_Down,CCL2(6347,1191856_1)_Down})=7.33
39	i({KAT2B(8850,1188483_1)_Down,CTBP1(1487,1193697_1)_Down,TP53(7157,1193761_1)_Down})=7.33

40	i({CRMP1(1400,1187700_1)_Up,NCOA2(10499,1177421_1)_Down,KAT2B(8850,1188483_1)_Down})=7.33
41	i({PABPN1(8106,1185655_1)_Up,CRMP1(1400,1187700_1)_Up,KAT2B(8850,1188483_1)_Down})=7.33
42	i({AQP2(359,1186541_1)_Up,CRMP1(1400,1187700_1)_Up,KAT2B(8850,1188483_1)_Down})=7.33
43	i({CRMP1(1400,1187700_1)_Up,KLK3(354,1181589_1)_Down,KAT2B(8850,1188483_1)_Down})=7.33
44	i({CDKN2B(1030,1179354_1)_Down,KAT2B(8850,1188483_1)_Down,CTBP1(1487,1193697_1)_Down})=7.33
45	i({KAT2B(8850,1188483_1)_Down,CTBP1(1487,1193697_1)_Down,SESN3(143686,1194275_1)_Down})=7.33
46	i({TEC(7006,1183972_1)_Up,CRMP1(1400,1187700_1)_Up,KAT2B(8850,1188483_1)_Down})=7.33
47	i({TEC(7006,1183972_1)_Up,KAT2B(8850,1188483_1)_Down,CTBP1(1487,1193697_1)_Down})=7.33
48	i({RB1(5925,1179746_1)_Up,CRMP1(1400,1187700_1)_Up,KAT2B(8850,1188483_1)_Down})=7.33
49	i({CRMP1(1400,1187700_1)_Up,IL6(3569,1184220_1)_Down,KAT2B(8850,1188483_1)_Down})=7.33
50	i({CRMP1(1400,1187700_1)_Up,U2AF1(7307,1190449_1)_Up,KAT2B(8850,1188483_1)_Down})=7.33

Supplementary Table 6. Top-50 impactful itemsets of F72A/R73A dataset in comparison 1with various degrees

Ranking	Impactful itemsets
1	i({KAT2B(8850,1188483_1)_Down,TP53(7157,1193761_1)_Down})=63.50
2	i({CCND1(595,1180551_1)_Down,TP53(7157,1193761_1)_Down})=54.00
3	i({CTBP1(1487,1193697_1)_Down,TP53(7157,1193761_1)_Down})=53.50
4	i({CRMP1(1400,1187700_1)_Up,TP53(7157,1193761_1)_Down})=53.50
5	i({NCOA1(8648,1179457_1)_Down,KAT2B(8850,1188483_1)_Down,TP53(7157,1193761_1)_Down})=50.33
6	i({CCND1(595,1180551_1)_Down,KAT2B(8850,1188483_1)_Down,TP53(7157,1193761_1)_Down})=49.33
7	i({TP53(7157,1193761_1)_Up,KAT2B(8850,1188483_1)_Down,TP53(7157,1193761_1)_Down})=49.00
8	i({JUP(3728,1174090_1)_Down,TP53(7157,1193761_1)_Down})=46.00
9	i({NCOA2(10499,1177421_1)_Down,KAT2B(8850,1188483_1)_Down,TP53(7157,1193761_1)_Down})=45.33
10	i({CCND1(595,1180551_1)_Down,CTBP1(1487,1193697_1)_Down,TP53(7157,1193761_1)_Down})=45.00
11	i({KAT2B(8850,1188483_1)_Down,MAPK1(5594,1192793_1)_Down,TP53(7157,1193761_1)_Down})=45.00
12	i({CRMP1(1400,1187700_1)_Up,KAT2B(8850,1188483_1)_Down,TP53(7157,1193761_1)_Down})=44.67
13	i({TEC(7006,1183972_1)_Up,KAT2B(8850,1188483_1)_Down,TP53(7157,1193761_1)_Down})=44.33
14	i({IL8(3576,1173646_1)_Down,KAT2B(8850,1188483_1)_Down,TP53(7157,1193761_1)_Down})=44.33
15	i({KAT2B(8850,1188483_1)_Down,CTBP1(1487,1193697_1)_Down,TP53(7157,1193761_1)_Down})=44.33
16	i({STAT3(6774,1182869_1)_Down,KAT2B(8850,1188483_1)_Down,TP53(7157,1193761_1)_Down})=44.33
17	i({KAT2B(8850,1188483_1)_Down,SMAD3(4088,1188764_1)_Down,TP53(7157,1193761_1)_Down})=44.33
18	i({NCOA2(10499,1183329_1)_Down,KAT2B(8850,1188483_1)_Down,TP53(7157,1193761_1)_Down})=44.33
19	i({NOTCH3(4854,1175161_1)_Down,KAT2B(8850,1188483_1)_Down,TP53(7157,1193761_1)_Down})=44.00
20	i({MAP2K6(5608,1185773_1)_Down,KAT2B(8850,1188483_1)_Down,TP53(7157,1193761_1)_Down})=44.00
21	i({TNF(7124,1189430_1)_Up,KAT2B(8850,1188483_1)_Down,TP53(7157,1193761_1)_Down})=44.00
22	i({MAPK8(5599,1188109_1)_Down,KAT2B(8850,1188483_1)_Down,TP53(7157,1193761_1)_Down})=44.00
23	i({MYC(4609,1180176_1)_Down,KAT2B(8850,1188483_1)_Down,TP53(7157,1193761_1)_Down})=44.00
24	i({CDKN2B(1030,1179354_1)_Down,KAT2B(8850,1188483_1)_Down,TP53(7157,1193761_1)_Down})=43.67
25	i({PAX6(5080,1183934_1)_Up,KAT2B(8850,1188483_1)_Down,TP53(7157,1193761_1)_Down})=43.67
26	i({NOS2(4843,1188233_1)_Up,KAT2B(8850,1188483_1)_Down,TP53(7157,1193761_1)_Down})=43.67
27	i({EPS15(2060,1191958_1)_Up,KAT2B(8850,1188483_1)_Down,TP53(7157,1193761_1)_Down})=43.67
28	i({KAT2B(8850,1188483_1)_Down,THBS1(7057,1189656_1)_Down,TP53(7157,1193761_1)_Down})=43.67
29	i({U2AF1(7307,1178903_1)_Up,KAT2B(8850,1188483_1)_Down,TP53(7157,1193761_1)_Down})=43.67
30	i({ZBTB16(7704,1177910_1)_Down,KAT2B(8850,1188483_1)_Down,TP53(7157,1193761_1)_Down})=43.67
31	i({ARNTL(406,1179501_1)_Down,KAT2B(8850,1188483_1)_Down,TP53(7157,1193761_1)_Down})=43.67
32	i({FIGF(2277,1175508_1)_Down,KAT2B(8850,1188483_1)_Down,TP53(7157,1193761_1)_Down})=43.67
33	i({IL12A(3592,1173656_1)_Down,KAT2B(8850,1188483_1)_Down,TP53(7157,1193761_1)_Down})=43.67
34	i({NR5A1(2516,1180799_1)_Up,KAT2B(8850,1188483_1)_Down,TP53(7157,1193761_1)_Down})=43.67
35	i({IRF7(3665,1181229_1)_Down,KAT2B(8850,1188483_1)_Down,TP53(7157,1193761_1)_Down})=43.67
36	i({CRMP1(1400,1187700_1)_Up,NCOA1(8648,1179457_1)_Down,TP53(7157,1193761_1)_Down})=43.67
37	i({IAPP(3375,1186981_1)_Down,KAT2B(8850,1188483_1)_Down,TP53(7157,1193761_1)_Down})=43.67
38	i({NOS2(4843,1188233_1)_Down,KAT2B(8850,1188483_1)_Down,TP53(7157,1193761_1)_Down})=43.67
39	i({GADD45G(10912,1175328_1)_Down,KAT2B(8850,1188483_1)_Down,TP53(7157,1193761_1)_Down})=43.33

40	i({PABPN1(8106,1185655_1)_Up,KAT2B(8850,1188483_1)_Down,TP53(7157,1193761_1)_Down})=43.33
41	i({KAT2B(8850,1188483_1)_Down,TP53(7157,1193761_1)_Down,SESN3(143686,1194275_1)_Down})=43.33
42	i({RB1(5925,1179746_1)_Up,KAT2B(8850,1188483_1)_Down,TP53(7157,1193761_1)_Down})=43.33
43	i({MAP2K1(5604,1174285_1)_Down,KAT2B(8850,1188483_1)_Down,TP53(7157,1193761_1)_Down})=43.33
44	i({KAT2B(8850,1188483_1)_Down,TP73(7161,1189560_1)_Down,TP53(7157,1193761_1)_Down})=43.33
45	i({ESR1(2099,1173928_1)_Down,KAT2B(8850,1188483_1)_Down,TP53(7157,1193761_1)_Down})=43.33
46	i({BAX(581,1181007_1)_Up,KAT2B(8850,1188483_1)_Down,TP53(7157,1193761_1)_Down})=43.33
47	i({PRKRA(8575,1176256_1)_Down,KAT2B(8850,1188483_1)_Down,TP53(7157,1193761_1)_Down})=43.33
48	i({SP3(6670,1188037_1)_Down,KAT2B(8850,1188483_1)_Down,TP53(7157,1193761_1)_Down})=43.33
49	i({GLI1(2735,1174154_1)_Down,KAT2B(8850,1188483_1)_Down,TP53(7157,1193761_1)_Down})=43.33
50	i({CIITA(4261,1174230_1)_Down,KAT2B(8850,1188483_1)_Down,TP53(7157,1193761_1)_Down})=43.33

Supplementary Table 7. Top-50 impactful itemsets of Wild type dataset in comparison 2 of undifferentiation control method

Ranking	Impactful itemsets
1	i({UBR1(197131,1177372_1)_Up,ELMO1(9844,1177818_1)_Up})=27.00
2	i({UBR1(197131,1177372_1)_Up,AJAP1(55966,1185257_1)_Up})=27.00
3	i({ELMO1(9844,1177818_1)_Up,AJAP1(55966,1185257_1)_Up})=27.00
4	i({UBR1(197131,1177372_1)_Up,CNOT4(4850,1189014_1)_Up})=27.00
5	i({ELMO1(9844,1177818_1)_Up,CNOT4(4850,1189014_1)_Up})=27.00
6	i({AJAP1(55966,1185257_1)_Up,CNOT4(4850,1189014_1)_Up})=27.00
7	i({UBR1(197131,1177372_1)_Up,ELMO1(9844,1177818_1)_Up,AJAP1(55966,1185257_1)_Up})=27.00
8	i({UBR1(197131,1177372_1)_Up,ELMO1(9844,1177818_1)_Up,CNOT4(4850,1189014_1)_Up})=27.00
9	i({UBR1(197131,1177372_1)_Up,AJAP1(55966,1185257_1)_Up,CNOT4(4850,1189014_1)_Up})=27.00
10	i({UBR1(197131,1177372_1)_Up,ELMO1(9844,1177818_1)_Up,AJAP1(55966,1185257_1)_Up,CNOT4(4850,1189014_1)_Up})=27.00
11	i({UBR1(197131,1177372_1)_Up,ELMO1(9844,1177818_1)_Up,AJAP1(55966,1185257_1)_Up,CNOT4(4850,1189014_1)_Up,TMSB4Y(9087,1173734_1)_Down})=26.80
12	i({UBR1(197131,1177372_1)_Up,ELMO1(9844,1177818_1)_Up,AJAP1(55966,1185257_1)_Up,CNOT4(4850,1189014_1)_Up,APIP(51074,1174466_1)_Down})=26.80
13	i({UBR1(197131,1177372_1)_Up,ELMO1(9844,1177818_1)_Up,AJAP1(55966,1185257_1)_Up,TMSB4Y(9087,1173734_1)_Down})=26.75
14	i({UBR1(197131,1177372_1)_Up,ELMO1(9844,1177818_1)_Up,CNOT4(4850,1189014_1)_Up,TMSB4Y(9087,1173734_1)_Down})=26.75
15	i({UBR1(197131,1177372_1)_Up,ELMO1(9844,1177818_1)_Up,AJAP1(55966,1185257_1)_Up,APIP(51074,1174466_1)_Down})=26.75
16	i({UBR1(197131,1177372_1)_Up,ELMO1(9844,1177818_1)_Up,CNOT4(4850,1189014_1)_Up,APIP(51074,1174466_1)_Down})=26.75
17	i({UBR1(197131,1177372_1)_Up,AJAP1(55966,1185257_1)_Up,CNOT4(4850,1189014_1)_Up,TMSB4Y(9087,1173734_1)_Down})=26.75
18	i({UBR1(197131,1177372_1)_Up,AJAP1(55966,1185257_1)_Up,CNOT4(4850,1189014_1)_Up,APIP(51074,1174466_1)_Down})=26.75
19	i({UBR1(197131,1177372_1)_Up,ELMO1(9844,1177818_1)_Up,AJAP1(55966,1185257_1)_Up,CNOT4(4850,1189014_1)_Up,TMSB4Y(9087,1173734_1)_Down,APIP(51074,1174466_1)_Down})=26.67
20	i({UBR1(197131,1177372_1)_Up,ELMO1(9844,1177818_1)_Up,TMSB4Y(9087,1173734_1)_Down})=26.67
21	i({UBR1(197131,1177372_1)_Up,AJAP1(55966,1185257_1)_Up,TMSB4Y(9087,1173734_1)_Down})=26.67
22	i({UBR1(197131,1177372_1)_Up,CNOT4(4850,1189014_1)_Up,TMSB4Y(9087,1173734_1)_Down})=26.67
23	i({UBR1(197131,1177372_1)_Up,ELMO1(9844,1177818_1)_Up,APIP(51074,1174466_1)_Down})=26.67
24	i({UBR1(197131,1177372_1)_Up,AJAP1(55966,1185257_1)_Up,APIP(51074,1174466_1)_Down})=26.67
25	i({UBR1(197131,1177372_1)_Up,CNOT4(4850,1189014_1)_Up,APIP(51074,1174466_1)_Down})=26.67
26	i({UBR1(197131,1177372_1)_Up,ELMO1(9844,1177818_1)_Up,AJAP1(55966,1185257_1)_Up,TMSB4Y(9087,1173734_1)_Down,APIP(51074,1174466_1)_Down})=26.60
27	i({UBR1(197131,1177372_1)_Up,ELMO1(9844,1177818_1)_Up,CCDC111(201973,1179751_1)_Up,AJAP1(55966,1185257_1)_Up,CNOT4(4850,1189014_1)_Up})=26.60
28	i({UBR1(197131,1177372_1)_Up,ELMO1(9844,1177818_1)_Up,AJAP1(55966,1185257_1)_Up,CNOT4(4850,1189014_1)_Up,HNF4G(3174,1173882_1)_Down})=26.60
29	i({UBR1(197131,1177372_1)_Up,ELMO1(9844,1177818_1)_Up,AJAP1(55966,1185257_1)_Up,CNOT4(4850,1189014_1)_Up,WDR52(55779,1174002_1)_Down})=26.60
30	i({UBR1(197131,1177372_1)_Up,ELMO1(9844,1177818_1)_Up,AJAP1(55966,1185257_1)_Up,CNOT4(4850,1189014_1)_Up,LOC100288281(100288281,1174004_1)_Down})=26.60

31	i({UBR1(197131,1177372_1)_Up,ELMO1(9844,1177818_1)_Up,AJAP1(55966,1185257_1)_Up,CNOT4(4850,1189014_1)_Up,NUP62(23636,1174117_1)_Down})=26.60
32	i({UBR1(197131,1177372_1)_Up,ELMO1(9844,1177818_1)_Up,PCIF1(63935,1185032_1)_Up,AJAP1(55966,1185257_1)_Up,CNOT4(4850,1189014_1)_Up})=26.60
33	i({UBR1(197131,1177372_1)_Up,ELMO1(9844,1177818_1)_Up,AJAP1(55966,1185257_1)_Up,CNOT4(4850,1189014_1)_Up,PBX3(5090,1177797_1)_Down})=26.60
34	i({UBR1(197131,1177372_1)_Up,ELMO1(9844,1177818_1)_Up,CNOT4(4850,1189014_1)_Up,TMSB4Y(9087,1173734_1)_Down,APIP(51074,1174466_1)_Down})=26.60
35	i({UBR1(197131,1177372_1)_Up,AJAP1(55966,1185257_1)_Up,CNOT4(4850,1189014_1)_Up,TMSB4Y(9087,1173734_1)_Down,APIP(51074,1174466_1)_Down})=26.60
36	i({UBR1(197131,1177372_1)_Up,TMSB4Y(9087,1173734_1)_Down})=26.50
37	i({ELMO1(9844,1177818_1)_Up,TMSB4Y(9087,1173734_1)_Down})=26.50
38	i({AJAP1(55966,1185257_1)_Up,TMSB4Y(9087,1173734_1)_Down})=26.50
39	i({CNOT4(4850,1189014_1)_Up,TMSB4Y(9087,1173734_1)_Down})=26.50
40	i({UBR1(197131,1177372_1)_Up,APIP(51074,1174466_1)_Down})=26.50
41	i({ELMO1(9844,1177818_1)_Up,APIP(51074,1174466_1)_Down})=26.50
42	i({AJAP1(55966,1185257_1)_Up,APIP(51074,1174466_1)_Down})=26.50
43	i({CNOT4(4850,1189014_1)_Up,APIP(51074,1174466_1)_Down})=26.50
44	i({UBR1(197131,1177372_1)_Up,ELMO1(9844,1177818_1)_Up,TMSB4Y(9087,1173734_1)_Down,APIP(51074,1174466_1)_Down})=26.50
45	i({UBR1(197131,1177372_1)_Up,ELMO1(9844,1177818_1)_Up,CCDC111(201973,1179751_1)_Up,AJAP1(55966,1185257_1)_Up})=26.50
46	i({UBR1(197131,1177372_1)_Up,ELMO1(9844,1177818_1)_Up,CCDC111(201973,1179751_1)_Up,CNOT4(4850,1189014_1)_Up})=26.50
47	i({UBR1(197131,1177372_1)_Up,ELMO1(9844,1177818_1)_Up,AJAP1(55966,1185257_1)_Up,HNF4G(3174,1173882_1)_Down})=26.50
48	i({UBR1(197131,1177372_1)_Up,ELMO1(9844,1177818_1)_Up,CNOT4(4850,1189014_1)_Up,HNF4G(3174,1173882_1)_Down})=26.50
49	i({UBR1(197131,1177372_1)_Up,ELMO1(9844,1177818_1)_Up,AJAP1(55966,1185257_1)_Up,WDR52(55779,1174002_1)_Down})=26.50
50	i({UBR1(197131,1177372_1)_Up,ELMO1(9844,1177818_1)_Up,CNOT4(4850,1189014_1)_Up,WDR52(55779,1174002_1)_Down})=26.50

Supplementary Table 8. Top-50 impactful itemsets of Wild type dataset in comparison 2 with a constant degree

Ranking	Impactful itemsets
1	i({STRAP(11171,1174318_1)_Up,MYC(4609,1180176_1)_Up})=14.50
2	i({MAP2K4(6416,1179586_1)_Up,MYC(4609,1180176_1)_Up})=13.00
3	i({STRAP(11171,1174318_1)_Up,MAP2K4(6416,1179586_1)_Up,MYC(4609,1180176_1)_Up})=13.00
4	i({STRAP(11171,1174318_1)_Up,MYC(4609,1180176_1)_Up,SOCS4(122809,1194141_1)_Down})=12.67
5	i({STRAP(11171,1174318_1)_Up,MAP2K4(6416,1179586_1)_Up,MYC(4609,1180176_1)_Up,BCL2(596,1180211_1)_Down})=12.50
6	i({STRAP(11171,1174318_1)_Up,MYC(4609,1180176_1)_Up,TCF7L2(6934,1187825_1)_Down})=12.33
7	i({STRAP(11171,1174318_1)_Up,MAP2K4(6416,1179586_1)_Up,RHOA(387,1179932_1)_Up,MYC(4609,1180176_1)_Up})=12.25
8	i({STRAP(11171,1174318_1)_Up,MYC(4609,1180176_1)_Up,GCK(2645,1173818_1)_Down})=12.00
9	i({STRAP(11171,1174318_1)_Up,MYC(4609,1180176_1)_Up,TYR(7299,1174020_1)_Down})=12.00
10	i({RHOA(387,1179932_1)_Up,MYC(4609,1180176_1)_Up})=12.00
11	i({STRAP(11171,1174318_1)_Up,MAP2K4(6416,1179586_1)_Up,MYC(4609,1180176_1)_Up,SOCS4(122809,1194141_1)_Down})=12.00
12	i({STRAP(11171,1174318_1)_Up,MYC(4609,1180176_1)_Up,CCND1(595,1180551_1)_Up})=12.00
13	i({STRAP(11171,1174318_1)_Up,MAP2K4(6416,1179586_1)_Up,MYC(4609,1180176_1)_Up,TCF7L2(6934,1187825_1)_Down})=11.75
14	i({STRAP(11171,1174318_1)_Up,MAP2K4(6416,1179586_1)_Up,MYC(4609,1180176_1)_Up,POU2AF1(5450,1174106_1)_Down})=11.75
15	i({STRAP(11171,1174318_1)_Up,RHOA(387,1179932_1)_Up,MYC(4609,1180176_1)_Up})=11.67
16	i({STRAP(11171,1174318_1)_Up,MYC(4609,1180176_1)_Up,CREBBP(1387,1176823_1)_Down})=11.67
17	i({SKI(6497,1174211_1)_Up,STRAP(11171,1174318_1)_Up,MYC(4609,1180176_1)_Up})=11.67
18	i({STRAP(11171,1174318_1)_Up,MYC(4609,1180176_1)_Up,NRIP1(8204,1187653_1)_Up})=11.67
19	i({MAP2K4(6416,1179586_1)_Up,MYC(4609,1180176_1)_Up,SOCS4(122809,1194141_1)_Down})=11.67
20	i({STRAP(11171,1174318_1)_Up,MYC(4609,1180176_1)_Up,NEUROD1(4760,1179370_1)_Down})=11.67
21	i({STRAP(11171,1174318_1)_Up,DNAJC3(5611,1174638_1)_Up,MYC(4609,1180176_1)_Up})=11.67
22	i({STRAP(11171,1174318_1)_Up,RB1CC1(9821,1178523_1)_Up,MYC(4609,1180176_1)_Up})=11.67
23	i({STRAP(11171,1174318_1)_Up,MYC(4609,1180176_1)_Up,CXCL11(6373,1184884_1)_Down})=11.67
24	i({STRAP(11171,1174318_1)_Up,RAN(5901,1175453_1)_Up,MYC(4609,1180176_1)_Up})=11.67
25	i({MYC(4609,1180176_1)_Up,BCL2(596,1180211_1)_Down})=11.50
26	i({STRAP(11171,1174318_1)_Up,MAP2K4(6416,1179586_1)_Up})=11.50
27	i({STRAP(11171,1174318_1)_Up,MAP2K4(6416,1179586_1)_Up,MYC(4609,1180176_1)_Up,TBP(6908,1173776_1)_Down})=11.50
28	i({STRAP(11171,1174318_1)_Up,MAP2K4(6416,1179586_1)_Up,MYC(4609,1180176_1)_Up,GCK(2645,1173818_1)_Down})=11.50
29	i({STRAP(11171,1174318_1)_Up,MAP2K4(6416,1179586_1)_Up,MYC(4609,1180176_1)_Up,TYR(7299,1174020_1)_Down})=11.50
30	i({STRAP(11171,1174318_1)_Up,MAP2K4(6416,1179586_1)_Up,MYC(4609,1180176_1)_Up,CCND1(595,1180551_1)_Up})=11.50
31	i({STRAP(11171,1174318_1)_Up,MYC(4609,1180176_1)_Up,BCL2(596,1180211_1)_Down})=11.33
32	i({STRAP(11171,1174318_1)_Up,MYC(4609,1180176_1)_Up,HNF4A(3172,1178626_1)_Down})=11.33
33	i({STRAP(11171,1174318_1)_Up,MYC(4609,1180176_1)_Up,MAPK1(5594,1192793_1)_Down})=11.33
34	i({STRAP(11171,1174318_1)_Up,MYC(4609,1180176_1)_Up,SPRY3(10251,1183715_1)_Down})=11.33
35	i({STRAP(11171,1174318_1)_Up,MYC(4609,1180176_1)_Up,FIS1(51024,1180669_1)_Up})=11.33
36	i({MAP2K4(6416,1179586_1)_Up,MYC(4609,1180176_1)_Up,TCF7L2(6934,1187825_1)_Down})=11.33
37	i({STRAP(11171,1174318_1)_Up,MYC(4609,1180176_1)_Up,PLAU(5328,1179933_1)_Down})=11.33
38	i({MAP2K4(6416,1179586_1)_Up,MYC(4609,1180176_1)_Up,POU2AF1(5450,1174106_1)_Down})=11.33
39	i({STRAP(11171,1174318_1)_Up,MYC(4609,1180176_1)_Up,SCNN1A(6337,1174175_1)_Down})=11.33

40	i({STRAP(11171,1174318_1)_Up,MYC(4609,1180176_1)_Up,IL18(3606,1180563_1)_Down})=11.33
41	i({STRAP(11171,1174318_1)_Up,PPP1R15A(23645,1179858_1)_Up,MYC(4609,1180176_1)_Up})=11.33
42	i({STRAP(11171,1174318_1)_Up,MYC(4609,1180176_1)_Up,IL1A(3552,1173944_1)_Down})=11.33
43	i({STRAP(11171,1174318_1)_Up,SNW1(22938,1180160_1)_Up,MYC(4609,1180176_1)_Up})=11.33
44	i({STRAP(11171,1174318_1)_Up,MYC(4609,1180176_1)_Up,GNA12(2768,1182206_1)_Up})=11.33
45	i({STRAP(11171,1174318_1)_Up,MYC(4609,1180176_1)_Up,RAC1(5879,1181740_1)_Up})=11.33
46	i({STRAP(11171,1174318_1)_Up,MYC(4609,1180176_1)_Up,STAT3(6774,1192670_1)_Up})=11.33
47	i({STRAP(11171,1174318_1)_Up,MAP2K4(6416,1179586_1)_Up,MYC(4609,1180176_1)_Up,CXCL11(6373,1184884_1)_Down})=11.25
48	i({SKI(6497,1174211_1)_Up,STRAP(11171,1174318_1)_Up,MAP2K4(6416,1179586_1)_Up,MYC(4609,1180176_1)_Up})=11.25
49	i({STRAP(11171,1174318_1)_Up,MAP2K4(6416,1179586_1)_Up,MYC(4609,1180176_1)_Up,NRIP1(8204,1187653_1)_Up})=11.25
50	i({STRAP(11171,1174318_1)_Up,MAP2K4(6416,1179586_1)_Up,MYC(4609,1180176_1)_Up,CREBBP(1387,1176823_1)_Down})=11.25

Supplementary Table 9. Top-50 impactful itemsets of Wild type dataset in comparison 2 with various degrees

**Ranking**	**Impactful itemsets**
1	i({STRAP(11171,1174318_1)_Up,MYC(4609,1180176_1)_Up})=61.00
2	i({MYC(4609,1180176_1)_Up,CCND1(595,1180551_1)_Up})=58.00
3	i({MAP2K4(6416,1179586_1)_Up,MYC(4609,1180176_1)_Up,CCND1(595,1180551_1)_Up})=54.00
4	i({MAP2K4(6416,1179586_1)_Up,MYC(4609,1180176_1)_Up,CREBBP(1387,1176823_1)_Down})=53.33
5	i({MAP2K4(6416,1179586_1)_Up,MYC(4609,1180176_1)_Up})=53.00
6	i({RHOA(387,1179932_1)_Up,MYC(4609,1180176_1)_Up})=52.00
7	i({MYC(4609,1180176_1)_Up,CREBBP(1387,1176823_1)_Down})=51.00
8	i({MYC(4609,1180176_1)_Up,CCND1(595,1180551_1)_Up,CREBBP(1387,1176823_1)_Down})=50.67
9	i({STRAP(11171,1174318_1)_Up,MYC(4609,1180176_1)_Up,STAT3(6774,1192670_1)_Up})=49.00
10	i({RHOA(387,1179932_1)_Up,MYC(4609,1180176_1)_Up,CCND1(595,1180551_1)_Up})=48.00
11	i({STRAP(11171,1174318_1)_Up,MAP2K4(6416,1179586_1)_Up,MYC(4609,1180176_1)_Up,CCND1(595,1180551_1)_Up})=47.00
12	i({STRAP(11171,1174318_1)_Up,MAP2K4(6416,1179586_1)_Up,MYC(4609,1180176_1)_Up,CREBBP(1387,1176823_1)_Down})=46.50
13	i({STRAP(11171,1174318_1)_Up,MYC(4609,1180176_1)_Up,TP53(7157,1193761_1)_Down})=46.33
14	i({STRAP(11171,1174318_1)_Up,MYC(4609,1180176_1)_Up,TCF7L2(6934,1187825_1)_Down})=46.00
15	i({STRAP(11171,1174318_1)_Up,MYC(4609,1180176_1)_Up,TNF(7124,1189430_1)_Down})=45.67
16	i({STRAP(11171,1174318_1)_Up,MYC(4609,1180176_1)_Up,FIS1(51024,1180669_1)_Up})=45.67
17	i({STRAP(11171,1174318_1)_Up,MYC(4609,1180176_1)_Up,IL8(3576,1173646_1)_Down})=45.67
18	i({MYC(4609,1180176_1)_Up,CCND1(595,1180551_1)_Up,STAT3(6774,1192670_1)_Up})=45.33
19	i({STRAP(11171,1174318_1)_Up,MYC(4609,1180176_1)_Up,GCK(2645,1173818_1)_Down})=45.33
20	i({STRAP(11171,1174318_1)_Up,MYC(4609,1180176_1)_Up,CCND1(595,1180551_1)_Up})=44.67
21	i({STRAP(11171,1174318_1)_Up,MYC(4609,1180176_1)_Up,FIGF(2277,1175508_1)_Down})=44.67
22	i({SKI(6497,1174211_1)_Up,STRAP(11171,1174318_1)_Up,MYC(4609,1180176_1)_Up})=44.67
23	i({MYC(4609,1180176_1)_Up,CCND1(595,1180551_1)_Up,POU2AF1(5450,1174106_1)_Down})=44.67
24	i({STRAP(11171,1174318_1)_Up,MYC(4609,1180176_1)_Up,CXCL11(6373,1184884_1)_Down})=44.67
25	i({STRAP(11171,1174318_1)_Up,MYC(4609,1180176_1)_Up,NEUROD1(4760,1179370_1)_Down})=44.67
26	i({MYC(4609,1180176_1)_Up,CCND1(595,1180551_1)_Up,TP53(7157,1193761_1)_Down})=44.33
27	i({STRAP(11171,1174318_1)_Up,MAP2K4(6416,1179586_1)_Up,MYC(4609,1180176_1)_Up})=44.00
28	i({STRAP(11171,1174318_1)_Up,MYC(4609,1180176_1)_Up,MAPK1(5594,1192793_1)_Down})=44.00
29	i({STRAP(11171,1174318_1)_Up,MYC(4609,1180176_1)_Up,IL1A(3552,1173944_1)_Down})=44.00
30	i({STRAP(11171,1174318_1)_Up,SNW1(22938,1180160_1)_Up,MYC(4609,1180176_1)_Up})=44.00
31	i({STRAP(11171,1174318_1)_Up,MYC(4609,1180176_1)_Up,PLAU(5328,1179933_1)_Down})=44.00
32	i({STRAP(11171,1174318_1)_Up,MYC(4609,1180176_1)_Up,IL18(3606,1180563_1)_Down})=44.00
33	i({MYC(4609,1180176_1)_Up,POU2AF1(5450,1174106_1)_Down,CREBBP(1387,1176823_1)_Down})=43.67
34	i({STRAP(11171,1174318_1)_Up,MYC(4609,1180176_1)_Up,SOCS4(122809,1194141_1)_Down})=43.67
35	i({STRAP(11171,1174318_1)_Up,MYC(4609,1180176_1)_Up,NCOA1(8648,1179457_1)_Down})=43.67
36	i({MAP2K4(6416,1179586_1)_Up,MYC(4609,1180176_1)_Up,STAT3(6774,1192670_1)_Up})=43.67
37	i({STRAP(11171,1174318_1)_Up,MYC(4609,1180176_1)_Up,IFNB1(3456,1184164_1)_Down})=43.33
38	i({STRAP(11171,1174318_1)_Up,MYC(4609,1180176_1)_Up,POU2AF1(5450,1174106_1)_Down})=43.33
39	i({STRAP(11171,1174318_1)_Up,MYC(4609,1180176_1)_Up,CXCL11(6373,1184884_1)_Up})=43.33

40	i({MYC(4609,1180176_1)_Up,POU2AF1(5450,1174106_1)_Down})=43.00
41	i({STRAP(11171,1174318_1)_Up,MYC(4609,1180176_1)_Up,TYR(7299,1174020_1)_Down})=43.00
42	i({STRAP(11171,1174318_1)_Up,MYC(4609,1180176_1)_Up,NRIP1(8204,1187653_1)_Up})=42.67
43	i({STRAP(11171,1174318_1)_Up,RAN(5901,1175453_1)_Up,MYC(4609,1180176_1)_Up})=42.67
44	i({STRAP(11171,1174318_1)_Up,MYC(4609,1180176_1)_Up,NCOA2(10499,1177421_1)_Down})=42.67
45	i({STRAP(11171,1174318_1)_Up,RB1CC1(9821,1178523_1)_Up,MYC(4609,1180176_1)_Up})=42.67
46	i({MYC(4609,1180176_1)_Up,CCND1(595,1180551_1)_Up,FIGF(2277,1175508_1)_Down})=42.67
47	i({STRAP(11171,1174318_1)_Up,MYC(4609,1180176_1)_Up,PDX1(3651,1177720_1)_Down})=42.67
48	i({STRAP(11171,1174318_1)_Up,MYC(4609,1180176_1)_Up,NCOA2(10499,1183329_1)_Down})=42.67
49	i({STRAP(11171,1174318_1)_Up,MYC(4609,1180176_1)_Up,TRAF2(7186,1188532_1)_Up})=42.67
50	i({STRAP(11171,1174318_1)_Up,MYC(4609,1180176_1)_Up,NEUROD1(4760,1194241_1)_Down})=42.67

Supplementary Table 10. Top-50 impactful itemsets of R80A dataset in comparison 2 of undifferentiation control method

**Ranking**	**Impactful itemsets**
1	i({MRPL39(54148,1174976_1)_Up,RUSC2(9853,1193203_1)_Up})=19.50
2	i({RUSC2(9853,1193203_1)_Up,TMCO7(79613,1182344_1)_Down})=19.50
3	i({TCOF1(6949,1190886_1)_Up,RUSC2(9853,1193203_1)_Up})=19.00
4	i({MRPL39(54148,1174976_1)_Up,RUSC2(9853,1193203_1)_Up,TMCO7(79613,1182344_1)_Down})=19.00
5	i({MRPL39(54148,1174976_1)_Up,DENND5B(160518,1185896_1)_Up,RUSC2(9853,1193203_1)_Up,TMCO7(79613,1182344_1)_Down})=19.00
6	i({DENND5B(160518,1185896_1)_Up,TCOF1(6949,1190886_1)_Up,RUSC2(9853,1193203_1)_Up,TMCO7(79613,1182344_1)_Down})=18.75
7	i({MRPL39(54148,1174976_1)_Up,DENND5B(160518,1185896_1)_Up,TCOF1(6949,1190886_1)_Up,RUSC2(9853,1193203_1)_Up})=18.75
8	i({TCOF1(6949,1190886_1)_Up,RUSC2(9853,1193203_1)_Up,TMCO7(79613,1182344_1)_Down})=18.67
9	i({SASH1(23328,1178567_1)_Up,DENND5B(160518,1185896_1)_Up,RUSC2(9853,1193203_1)_Up})=18.67
10	i({OR2H1(26716,1185702_1)_Up,RUSC2(9853,1193203_1)_Up,TMCO7(79613,1182344_1)_Down})=18.67
11	i({MRPL39(54148,1174976_1)_Up,TCOF1(6949,1190886_1)_Up,RUSC2(9853,1193203_1)_Up})=18.67
12	i({MRPL39(54148,1174976_1)_Up,DENND5B(160518,1185896_1)_Up,TCOF1(6949,1190886_1)_Up,RUSC2(9853,1193203_1)_Up,TMCO7(79613,1182344_1)_Down})=18.60
13	i({DENND5B(160518,1185896_1)_Up,RUSC2(9853,1193203_1)_Up})=18.50
14	i({OR2H1(26716,1185702_1)_Up,RUSC2(9853,1193203_1)_Up})=18.50
15	i({SASH1(23328,1178567_1)_Up,RUSC2(9853,1193203_1)_Up})=18.50
16	i({MRPL39(54148,1174976_1)_Up,TCOF1(6949,1190886_1)_Up,RUSC2(9853,1193203_1)_Up,TMCO7(79613,1182344_1)_Down})=18.50
17	i({MRPL39(54148,1174976_1)_Up,SASH1(23328,1178567_1)_Up,DENND5B(160518,1185896_1)_Up,RUSC2(9853,1193203_1)_Up})=18.50
18	i({SASH1(23328,1178567_1)_Up,DENND5B(160518,1185896_1)_Up,RUSC2(9853,1193203_1)_Up,TMCO7(79613,1182344_1)_Down})=18.50
19	i({MRPL39(54148,1174976_1)_Up,OR2H1(26716,1185702_1)_Up,RUSC2(9853,1193203_1)_Up,TMCO7(79613,1182344_1)_Down})=18.50
20	i({MRPL39(54148,1174976_1)_Up,SASH1(23328,1178567_1)_Up,DENND5B(160518,1185896_1)_Up,RUSC2(9853,1193203_1)_Up,TMCO7(79613,1182344_1)_Down})=18.40
21	i({SLC6A12(6539,1185358_1)_Up,RUSC2(9853,1193203_1)_Up,TMCO7(79613,1182344_1)_Down})=18.33
22	i({MRPL39(54148,1174976_1)_Up,SASH1(23328,1178567_1)_Up,RUSC2(9853,1193203_1)_Up})=18.33
23	i({SASH1(23328,1178567_1)_Up,RUSC2(9853,1193203_1)_Up,TMCO7(79613,1182344_1)_Down})=18.33
24	i({MRPL39(54148,1174976_1)_Up,DENND5B(160518,1185896_1)_Up,TMCO7(79613,1182344_1)_Down})=18.33
25	i({DENND5B(160518,1185896_1)_Up,PDS5B(23047,1190115_1)_Up,RUSC2(9853,1193203_1)_Up})=18.33
26	i({DENND5B(160518,1185896_1)_Up,SEMA3G(56920,1187952_1)_Up,RUSC2(9853,1193203_1)_Up})=18.33
27	i({MRPL39(54148,1174976_1)_Up,SLC6A12(6539,1185358_1)_Up,RUSC2(9853,1193203_1)_Up})=18.33
28	i({MRPL39(54148,1174976_1)_Up,SASH1(23328,1178567_1)_Up,RUSC2(9853,1193203_1)_Up,TMCO7(79613,1182344_1)_Down})=18.25
29	i({MRPL39(54148,1174976_1)_Up,DENND5B(160518,1185896_1)_Up,SEMA3G(56920,1187952_1)_Up,RUSC2(9853,1193203_1)_Up})=18.25
30	i({SASH1(23328,1178567_1)_Up,DENND5B(160518,1185896_1)_Up,TCOF1(6949,1190886_1)_Up,RUSC2(9853,1193203_1)_Up})=18.25
31	i({MRPL39(54148,1174976_1)_Up,DENND5B(160518,1185896_1)_Up,PDS5B(23047,1190115_1)_Up,RUSC2(9853,1193203_1)_Up})=18.25
32	i({DENND5B(160518,1185896_1)_Up,PDS5B(23047,1190115_1)_Up,RUSC2(9853,1193203_1)_Up,TMCO7(79613,1182344_1)_Down})=18.25
33	i({DENND5B(160518,1185896_1)_Up,SEMA3G(56920,1187952_1)_Up,RUSC2(9853,1193203_1)_Up,TMCO7(79613,1182344_1)_Down})=18.25
34	i({MRPL39(54148,1174976_1)_Up,SLC6A12(6539,1185358_1)_Up,RUSC2(9853,1193203_1)_Up,TMCO7(79613,1182344_1)_Down})=18.25

35	i({OR2H1(26716,1185702_1)_Up,TCOF1(6949,1190886_1)_Up,RUSC2(9853,1193203_1)_Up,TMCO7(79613,1182344_1)_Down})=18.25
36	i({MRPL39(54148,1174976_1)_Up,OR2H1(26716,1185702_1)_Up,TCOF1(6949,1190886_1)_Up,RUSC2(9853,1193203_1)_Up})=18.25
37	i({MRPL39(54148,1174976_1)_Up,OR2H1(26716,1185702_1)_Up,TCOF1(6949,1190886_1)_Up,RUSC2(9853,1193203_1)_Up,TMCO7(79613,1182344_1)_Down})=18.20
38	i({MRPL39(54148,1174976_1)_Up,SEMA3G(56920,1187952_1)_Up,RUSC2(9853,1193203_1)_Up})=18.00
39	i({AQPEP(206338,1179483_1)_Up,RUSC2(9853,1193203_1)_Up})=18.00
40	i({MRPL39(54148,1174976_1)_Up,TMCO7(79613,1182344_1)_Down})=18.00
41	i({PDS5B(23047,1190115_1)_Up,RUSC2(9853,1193203_1)_Up})=18.00
42	i({SEMA3G(56920,1187952_1)_Up,RUSC2(9853,1193203_1)_Up})=18.00
43	i({MRPL39(54148,1174976_1)_Up,OR2H1(26716,1185702_1)_Up,RUSC2(9853,1193203_1)_Up})=18.00
44	i({SASH1(23328,1178567_1)_Up,TCOF1(6949,1190886_1)_Up,RUSC2(9853,1193203_1)_Up})=18.00
45	i({MRPL39(54148,1174976_1)_Up,PDS5B(23047,1190115_1)_Up,RUSC2(9853,1193203_1)_Up})=18.00
46	i({PDS5B(23047,1190115_1)_Up,RUSC2(9853,1193203_1)_Up,TMCO7(79613,1182344_1)_Down})=18.00
47	i({SEMA3G(56920,1187952_1)_Up,RUSC2(9853,1193203_1)_Up,TMCO7(79613,1182344_1)_Down})=18.00
48	i({OR2H1(26716,1185702_1)_Up,DENND5B(160518,1185896_1)_Up,RUSC2(9853,1193203_1)_Up})=18.00
49	i({MRPL39(54148,1174976_1)_Up,DENND5B(160518,1185896_1)_Up,TCOF1(6949,1190886_1)_Up})=18.00
50	i({DENND5B(160518,1185896_1)_Up,TCOF1(6949,1190886_1)_Up,TMCO7(79613,1182344_1)_Down})=18.00

Supplementary Table 11. Top-50 impactful itemsets of R80A dataset in comparison 2 with a constant degree

Ranking	Impactful itemsets
1	i({DPYSL2(1808,1181711_1)_Up,MSX1(4487,1173880_1)_Down})=11.00
2	i({RB1(5925,1179746_1)_Up,DPYSL2(1808,1181711_1)_Up,MSX1(4487,1173880_1)_Down})=10.33
3	i({STAT1(6772,1176550_1)_Up,DPYSL2(1808,1181711_1)_Up,MSX1(4487,1173880_1)_Down})=10.00
4	i({RB1(5925,1179746_1)_Up,DPYSL2(1808,1181711_1)_Up})=10.00
5	i({DPYSL2(1808,1181711_1)_Up,MSX1(4487,1173880_1)_Down,FIGF(2277,1175508_1)_Down})=9.67
6	i({DPYSL2(1808,1181711_1)_Up,TEC(7006,1183972_1)_Up,MSX1(4487,1173880_1)_Down})=9.67
7	i({DPYSL2(1808,1181711_1)_Up,RBMS1(5937,1189342_1)_Up})=9.50
8	i({STAT1(6772,1176550_1)_Up,RB1(5925,1179746_1)_Up,DPYSL2(1808,1181711_1)_Up})=9.33
9	i({DPYSL2(1808,1181711_1)_Up,IL12A(3592,1173656_1)_Down,MSX1(4487,1173880_1)_Down})=9.33
10	i({DPYSL2(1808,1181711_1)_Up,MSX1(4487,1173880_1)_Down,PDX1(3651,1177720_1)_Down})=9.33
11	i({DPYSL2(1808,1181711_1)_Up,KAT2B(8850,1188483_1)_Down})=9.00
12	i({RB1(5925,1179746_1)_Up,DPYSL2(1808,1181711_1)_Up,RBMS1(5937,1189342_1)_Up})=9.00
13	i({DPYSL2(1808,1181711_1)_Up,RBMS1(5937,1189342_1)_Up,MSX1(4487,1173880_1)_Down})=9.00
14	i({RB1(5925,1179746_1)_Up,DPYSL2(1808,1181711_1)_Up,FIGF(2277,1175508_1)_Down})=9.00
15	i({DPYSL2(1808,1181711_1)_Up,RBMS1(5937,1189342_1)_Up,KAT2B(8850,1188483_1)_Down})=9.00
16	i({DPYSL2(1808,1181711_1)_Up,MSX1(4487,1173880_1)_Down,MYC(4609,1180176_1)_Down})=9.00
17	i({RB1(5925,1179746_1)_Up,DPYSL2(1808,1181711_1)_Up,TEC(7006,1183972_1)_Up})=9.00
18	i({DPYSL2(1808,1181711_1)_Up,MSX1(4487,1173880_1)_Down,TP53(7157,1193761_1)_Down})=9.00
19	i({DPYSL2(1808,1181711_1)_Up,MSX1(4487,1173880_1)_Down,ARHGEF12(23365,1192025_1)_Down})=9.00
20	i({DPYSL2(1808,1181711_1)_Up,MSX1(4487,1173880_1)_Down,IAPP(3375,1186981_1)_Down})=9.00
21	i({DPYSL2(1808,1181711_1)_Up,MSX1(4487,1173880_1)_Down,GNA13(10672,1173929_1)_Down})=9.00
22	i({DPYSL2(1808,1181711_1)_Up,CEBPB(1051,1184540_1)_Up,MSX1(4487,1173880_1)_Down})=9.00
23	i({DPYSL2(1808,1181711_1)_Up,POLA1(5422,1190258_1)_Up,MSX1(4487,1173880_1)_Down})=9.00
24	i({DPYSL2(1808,1181711_1)_Up,MSX1(4487,1173880_1)_Down,CIITA(4261,1174230_1)_Down})=9.00
25	i({STAT1(6772,1176550_1)_Up,RB1(5925,1179746_1)_Up,DPYSL2(1808,1181711_1)_Up,MSX1(4487,1173880_1)_Down})=9.00
26	i({RB1(5925,1179746_1)_Up,DPYSL2(1808,1181711_1)_Up,MSX1(4487,1173880_1)_Down,KAT2B(8850,1188483_1)_Down})=9.00
27	i({RB1(5925,1179746_1)_Up,DPYSL2(1808,1181711_1)_Up,RBMS1(5937,1189342_1)_Up,MSX1(4487,1173880_1)_Down})=8.75
28	i({RB1(5925,1179746_1)_Up,DPYSL2(1808,1181711_1)_Up,PABPN1(8106,1185655_1)_Up,MSX1(4487,1173880_1)_Down})=8.75
29	i({RB1(5925,1179746_1)_Up,DPYSL2(1808,1181711_1)_Up,MSX1(4487,1173880_1)_Down,FIGF(2277,1175508_1)_Down})=8.75
30	i({STAT1(6772,1176550_1)_Up,DPYSL2(1808,1181711_1)_Up,RBMS1(5937,1189342_1)_Up,MSX1(4487,1173880_1)_Down})=8.75
31	i({DPYSL2(1808,1181711_1)_Up,RBMS1(5937,1189342_1)_Up,MSX1(4487,1173880_1)_Down,KAT2B(8850,1188483_1)_Down})=8.75
32	i({RB1(5925,1179746_1)_Up,DPYSL2(1808,1181711_1)_Up,TEC(7006,1183972_1)_Up,MSX1(4487,1173880_1)_Down})=8.75
33	i({DPYSL2(1808,1181711_1)_Up,MSX1(4487,1173880_1)_Down,ETS1(2113,1176218_1)_Down})=8.67
34	i({DPYSL2(1808,1181711_1)_Up,KHDRBS1(10657,1185831_1)_Up,MSX1(4487,1173880_1)_Down})=8.67
35	i({DPYSL2(1808,1181711_1)_Up,RAC1(5879,1181740_1)_Up,MSX1(4487,1173880_1)_Down})=8.67
36	i({DPYSL2(1808,1181711_1)_Up,IL8(3576,1173646_1)_Down,MSX1(4487,1173880_1)_Down})=8.67
37	i({DPYSL2(1808,1181711_1)_Up,PABPN1(8106,1185655_1)_Up,RBMS1(5937,1189342_1)_Up})=8.67
38	i({DPYSL2(1808,1181711_1)_Up,ELK1(2002,1182086_1)_Up,MSX1(4487,1173880_1)_Down})=8.67
39	i({RB1(5925,1179746_1)_Up,DPYSL2(1808,1181711_1)_Up,IL12A(3592,1173656_1)_Down})=8.67

40	i({DPYSL2(1808,1181711_1)_Up,SIAH1(6477,1183236_1)_Up,MSX1(4487,1173880_1)_Down})=8.67
41	i({DPYSL2(1808,1181711_1)_Up,RASA1(5921,1173683_1)_Down,MSX1(4487,1173880_1)_Down})=8.67
42	i({DPYSL2(1808,1181711_1)_Up,MSX1(4487,1173880_1)_Down,ARHGEF12(23365,1174760_1)_Down})=8.67
43	i({DPYSL2(1808,1181711_1)_Up,MSX1(4487,1173880_1)_Down,NCOA1(8648,1179457_1)_Down})=8.67
44	i({LEF1(51176,1178271_1)_Up,DPYSL2(1808,1181711_1)_Up,MSX1(4487,1173880_1)_Down})=8.67
45	i({DPYSL2(1808,1181711_1)_Up,MSX1(4487,1173880_1)_Down,VDR(7421,1174023_1)_Down})=8.67
46	i({RB1(5925,1179746_1)_Up,MSX1(4487,1173880_1)_Down})=8.50
47	i({RB1(5925,1179746_1)_Up,RBMS1(5937,1189342_1)_Up})=8.50
48	i({STAT1(6772,1176550_1)_Up,RB1(5925,1179746_1)_Up})=8.50
49	i({DPYSL2(1808,1181711_1)_Up,PABPN1(8106,1185655_1)_Up})=8.50
50	i({STAT1(6772,1176550_1)_Up,RBMS1(5937,1189342_1)_Up})=8.50

Supplementary Table 12. Top-50 impactful itemsets of R80A dataset in comparison 2 with various degrees

Ranking	Impactful itemsets
1	i({DPYSL2(1808,1181711_1)_Up,TP53(7157,1193761_1)_Down})=46.00
2	i({RB1(5925,1179746_1)_Up,TP53(7157,1193761_1)_Down})=45.50
3	i({MSX1(4487,1173880_1)_Down,TP53(7157,1193761_1)_Down})=45.00
4	i({CEBPA(1050,1179814_1)_Down,TP53(7157,1193761_1)_Down})=40.00
5	i({SMURF1(57154,1192023_1)_Down,TP53(7157,1193761_1)_Down})=38.50
6	i({TEC(7006,1183972_1)_Up,TP53(7157,1193761_1)_Down})=36.00
7	i({GNA13(10672,1173929_1)_Down,TP53(7157,1193761_1)_Down})=36.00
8	i({DPYSL2(1808,1181711_1)_Up,FIGF(2277,1175508_1)_Down,TP53(7157,1193761_1)_Down})=36.00
9	i({RB1(5925,1179746_1)_Up,FIGF(2277,1175508_1)_Down,TP53(7157,1193761_1)_Down})=35.67
10	i({RBMS1(5937,1189342_1)_Up,TP53(7157,1193761_1)_Down})=35.50
11	i({MSX1(4487,1173880_1)_Down,FIGF(2277,1175508_1)_Down,TP53(7157,1193761_1)_Down})=35.33
12	i({STAT1(6772,1176550_1)_Up,DPYSL2(1808,1181711_1)_Up,TP53(7157,1193761_1)_Down})=34.67
13	i({DPYSL2(1808,1181711_1)_Up,CEBPA(1050,1179814_1)_Down,TP53(7157,1193761_1)_Down})=34.67
14	i({STAT1(6772,1176550_1)_Up,RB1(5925,1179746_1)_Up,TP53(7157,1193761_1)_Down})=34.33
15	i({RB1(5925,1179746_1)_Up,CEBPA(1050,1179814_1)_Down,TP53(7157,1193761_1)_Down})=34.33
16	i({STAT1(6772,1176550_1)_Up,MSX1(4487,1173880_1)_Down,TP53(7157,1193761_1)_Down})=34.00
17	i({MSX1(4487,1173880_1)_Down,CEBPA(1050,1179814_1)_Down,TP53(7157,1193761_1)_Down})=34.00
18	i({DPYSL2(1808,1181711_1)_Up,SMURF1(57154,1192023_1)_Down,TP53(7157,1193761_1)_Down})=33.67
19	i({LEF1(51176,1178271_1)_Up,DPYSL2(1808,1181711_1)_Up,TP53(7157,1193761_1)_Down})=33.33
20	i({RB1(5925,1179746_1)_Up,SMURF1(57154,1192023_1)_Down,TP53(7157,1193761_1)_Down})=33.33
21	i({MSX1(4487,1173880_1)_Down,SMURF1(57154,1192023_1)_Down,TP53(7157,1193761_1)_Down})=33.00
22	i({LEF1(51176,1178271_1)_Up,RB1(5925,1179746_1)_Up,TP53(7157,1193761_1)_Down})=33.00
23	i({RB1(5925,1179746_1)_Up,DPYSL2(1808,1181711_1)_Up,TP53(7157,1193761_1)_Down})=32.67
24	i({DPYSL2(1808,1181711_1)_Up,KAT2B(8850,1188483_1)_Down,TP53(7157,1193761_1)_Down})=32.67
25	i({DPYSL2(1808,1181711_1)_Up,MYC(4609,1180176_1)_Down,TP53(7157,1193761_1)_Down})=32.67
26	i({DPYSL2(1808,1181711_1)_Up,IL12A(3592,1173656_1)_Down,TP53(7157,1193761_1)_Down})=32.67
27	i({DPYSL2(1808,1181711_1)_Up,IL6(3569,1184220_1)_Down,TP53(7157,1193761_1)_Down})=32.67
28	i({DPYSL2(1808,1181711_1)_Up,NCOA1(8648,1179457_1)_Down,TP53(7157,1193761_1)_Down})=32.67
29	i({LEF1(51176,1178271_1)_Up,MSX1(4487,1173880_1)_Down,TP53(7157,1193761_1)_Down})=32.67
30	i({DPYSL2(1808,1181711_1)_Up,SMAD3(4088,1191570_1)_Down,TP53(7157,1193761_1)_Down})=32.67
31	i({DPYSL2(1808,1181711_1)_Up,MSX1(4487,1173880_1)_Down,TP53(7157,1193761_1)_Down})=32.33
32	i({NFKB1(4790,1180596_1)_Up,DPYSL2(1808,1181711_1)_Up,TP53(7157,1193761_1)_Down})=32.33
33	i({RB1(5925,1179746_1)_Up,KAT2B(8850,1188483_1)_Down,TP53(7157,1193761_1)_Down})=32.33
34	i({DPYSL2(1808,1181711_1)_Up,STAT3(6774,1192670_1)_Up,TP53(7157,1193761_1)_Down})=32.33
35	i({RB1(5925,1179746_1)_Up,IL12A(3592,1173656_1)_Down,TP53(7157,1193761_1)_Down})=32.33
36	i({RB1(5925,1179746_1)_Up,MYC(4609,1180176_1)_Down,TP53(7157,1193761_1)_Down})=32.33
37	i({DPYSL2(1808,1181711_1)_Up,IL8(3576,1173646_1)_Down,TP53(7157,1193761_1)_Down})=32.33
38	i({RB1(5925,1179746_1)_Up,IL6(3569,1184220_1)_Down,TP53(7157,1193761_1)_Down})=32.33
39	i({RB1(5925,1179746_1)_Up,NCOA1(8648,1179457_1)_Down,TP53(7157,1193761_1)_Down})=32.33

40	i({RB1(5925,1179746_1)_Up,SMAD3(4088,1191570_1)_Down,TP53(7157,1193761_1)_Down})=32.33
41	i({FIGF(2277,1175508_1)_Down,CEBPA(1050,1179814_1)_Down,TP53(7157,1193761_1)_Down})=32.00
42	i({DPYSL2(1808,1181711_1)_Up,ETS1(2113,1176218_1)_Down,TP53(7157,1193761_1)_Down})=32.00
43	i({IL12A(3592,1173656_1)_Down,MSX1(4487,1173880_1)_Down,TP53(7157,1193761_1)_Down})=32.00
44	i({RB1(5925,1179746_1)_Up,NFKB1(4790,1180596_1)_Up,TP53(7157,1193761_1)_Down})=32.00
45	i({MSX1(4487,1173880_1)_Down,MYC(4609,1180176_1)_Down,TP53(7157,1193761_1)_Down})=32.00
46	i({DPYSL2(1808,1181711_1)_Up,PDX1(3651,1177720_1)_Down,TP53(7157,1193761_1)_Down})=32.00
47	i({DPYSL2(1808,1181711_1)_Up,RASA1(5921,1173683_1)_Down,TP53(7157,1193761_1)_Down})=32.00
48	i({RB1(5925,1179746_1)_Up,IL8(3576,1173646_1)_Down,TP53(7157,1193761_1)_Down})=32.00
49	i({DPYSL2(1808,1181711_1)_Up,TEC(7006,1183972_1)_Up,TP53(7157,1193761_1)_Down})=32.00
50	i({MSX1(4487,1173880_1)_Down,IL6(3569,1184220_1)_Down,TP53(7157,1193761_1)_Down})=32.00

Supplementary Table 13. Top-50 impactful itemsets of Air dataset in comparison 3 of undifferentiation control method

Ranking	Impactful itemsets
1	i({Mela(17276,1433438_x_at)_Down,Mela(17276,1456182_x_at)_Down})=48.00
2	i({Mela(17276,1433438_x_at)_Down,Mela(17276,1456456_x_at)_Down})=48.00
3	i({Mela(17276,1456182_x_at)_Down,Mela(17276,1456456_x_at)_Down})=48.00
4	i({Mela(17276,1433438_x_at)_Down,Mela(17276,1456182_x_at)_Down,Mela(17276,1456456_x_at)_Down})=48.00
5	i({Mela(17276,1433438_x_at)_Down,Fam167b(230766,1455587_at)_Down,Mela(17276,1456182_x_at)_Down,Mela(17276,1456456_x_at)_Down})=47.75
6	i({Mela(17276,1433438_x_at)_Down,Fam167b(230766,1455587_at)_Down,Mela(17276,1456182_x_at)_Down})=47.67
7	i({Mela(17276,1433438_x_at)_Down,Fam167b(230766,1455587_at)_Down,Mela(17276,1456456_x_at)_Down})=47.67
8	i({Mela(17276,1433438_x_at)_Down,Fam167b(230766,1455587_at)_Down})=47.50
9	i({Fam167b(230766,1455587_at)_Down,Mela(17276,1456182_x_at)_Down})=47.50
10	i({Fam167b(230766,1455587_at)_Down,Mela(17276,1456456_x_at)_Down})=47.50
11	i({BC018473(193217,1427451_a_at)_Down,Mela(17276,1433438_x_at)_Down,Mela(17276,1456182_x_at)_Down,Mela(17276,1456456_x_at)_Down})=47.00
12	i({Mela(17276,1433438_x_at)_Down,Mycl1(16918,1434777_at)_Down,Mela(17276,1456182_x_at)_Down,Mela(17276,1456456_x_at)_Down})=47.00
13	i({Mela(17276,1433438_x_at)_Down,Bola2(66162,1434544_at)_Down,Mela(17276,1456182_x_at)_Down,Mela(17276,1456456_x_at)_Down})=47.00
14	i({BC018473(193217,1427451_a_at)_Down,Mela(17276,1433438_x_at)_Down,Fam167b(230766,1455587_at)_Down,Mela(17276,1456182_x_at)_Down,Mela(17276,1456456_x_at)_Down})=47.00
15	i({Mela(17276,1433438_x_at)_Down,Bola2(66162,1434544_at)_Down,Fam167b(230766,1455587_at)_Down,Mela(17276,1456182_x_at)_Down,Mela(17276,1456456_x_at)_Down})=47.00
16	i({Mela(17276,1433438_x_at)_Down,Mycl1(16918,1434777_at)_Down,Fam167b(230766,1455587_at)_Down,Mela(17276,1456182_x_at)_Down,Mela(17276,1456456_x_at)_Down})=47.00
17	i({Dhcr24(74754,1418129_at)_Down,Mela(17276,1433438_x_at)_Down,Fam167b(230766,1455587_at)_Down,Mela(17276,1456182_x_at)_Down,Mela(17276,1456456_x_at)_Down})=46.80
18	i({Mela(17276,1433438_x_at)_Down,0610011L14Rik(68295,1437835_a_at)_Down,Fam167b(230766,1455587_at)_Down,Mela(17276,1456182_x_at)_Down,Mela(17276,1456456_x_at)_Down})=46.80
19	i({LOC433053(433053,1424931_s_at)_Down,Mela(17276,1433438_x_at)_Down,Fam167b(230766,1455587_at)_Down,Mela(17276,1456182_x_at)_Down,Mela(17276,1456456_x_at)_Down})=46.80
20	i({Cntn1(12805,1452981_at)_Up,Mela(17276,1433438_x_at)_Down,Fam167b(230766,1455587_at)_Down,Mela(17276,1456182_x_at)_Down, Mela(17276,1456456_x_at)_Down})=46.80
21	i({Mela(17276,1433438_x_at)_Down,Dbp(13170,1438211_s_at)_Down,Fam167b(230766,1455587_at)_Down,Mela(17276,1456182_x_at)_Down,Mela(17276,1456456_x_at)_Down})=46.80
22	i({BC018473(193217,1427451_a_at)_Down,Mela(17276,1433438_x_at)_Down,Fam167b(230766,1455587_at)_Down,Mela(17276,1456182_x_at)_Down})=46.75
23	i({Mela(17276,1433438_x_at)_Down,Bola2(66162,1434544_at)_Down,Fam167b(230766,1455587_at)_Down,Mela(17276,1456182_x_at)_Down})=46.75
24	i({Mela(17276,1433438_x_at)_Down,Mycl1(16918,1434777_at)_Down,Fam167b(230766,1455587_at)_Down,Mela(17276,1456182_x_at)_Down})=46.75

25	i({Dhcr24(74754,1418129_at)_Down,Mela(17276,1433438_x_at)_Down,Mela(17276,1456182_x_at)_Down,Mela(17276,1456456_x_at)_Down})=46.75
26	i({LOC433053(433053,1424931_s_at)_Down,Mela(17276,1433438_x_at)_Down,Mela(17276,1456182_x_at)_Down,Mela(17276,1456456_x_at)_Down})=46.75
27	i({Cntn1(12805,1452981_at)_Up,Mela(17276,1433438_x_at)_Down,Mela(17276,1456182_x_at)_Down,Mela(17276,1456456_x_at)_Down})=46.75
28	i({Mela(17276,1433438_x_at)_Down,0610011L14Rik(68295,1437835_a_at)_Down,Mela(17276,1456182_x_at)_Down,Mela(17276,1456456_x_at)_Down})=46.75
29	i({Mela(17276,1433438_x_at)_Down,Dbp(13170,1438211_s_at)_Down,Mela(17276,1456182_x_at)_Down,Mela(17276,1456456_x_at)_Down})=46.75
30	i({BC018473(193217,1427451_a_at)_Down,Mela(17276,1433438_x_at)_Down,Fam167b(230766,1455587_at)_Down,Mela(17276,1456456_x_at)_Down})=46.75
31	i({Mela(17276,1433438_x_at)_Down,Bola2(66162,1434544_at)_Down,Fam167b(230766,1455587_at)_Down,Mela(17276,1456456_x_at)_Down})=46.75
32	i({Mela(17276,1433438_x_at)_Down,Mycl1(16918,1434777_at)_Down,Fam167b(230766,1455587_at)_Down,Mela(17276,1456456_x_at)_Down})=46.75
33	i({BC018473(193217,1427451_a_at)_Down,Mela(17276,1433438_x_at)_Down,Mela(17276,1456182_x_at)_Down})=46.67
34	i({BC018473(193217,1427451_a_at)_Down,Mela(17276,1433438_x_at)_Down,Mela(17276,1456456_x_at)_Down})=46.67
35	i({Mela(17276,1433438_x_at)_Down,Mycl1(16918,1434777_at)_Down,Mela(17276,1456182_x_at)_Down})=46.67
36	i({Mela(17276,1433438_x_at)_Down,Mycl1(16918,1434777_at)_Down,Mela(17276,1456456_x_at)_Down})=46.67
37	i({Mela(17276,1433438_x_at)_Down,Bola2(66162,1434544_at)_Down,Mela(17276,1456182_x_at)_Down})=46.67
38	i({Mela(17276,1433438_x_at)_Down,Bola2(66162,1434544_at)_Down,Mela(17276,1456456_x_at)_Down})=46.67
39	i({Mela(17276,1433438_x_at)_Down,Slc25a13(50799,1449481_at)_Down,Fam167b(230766,1455587_at)_Down,Mela(17276,1456182_x_at)_Down,Mela(17276,1456456_x_at)_Down})=46.60
40	i({Osr2(107587,1426155_a_at)_Down,Mela(17276,1433438_x_at)_Down,Fam167b(230766,1455587_at)_Down,Mela(17276,1456182_x_at)_Down,Mela(17276,1456456_x_at)_Down})=46.60
41	i({Mela(17276,1433438_x_at)_Down,Erlin1(226144,1441344_at)_Down,Fam167b(230766,1455587_at)_Down,Mela(17276,1456182_x_at)_Down,Mela(17276,1456456_x_at)_Down})=46.60
42	i({Dhcr24(74754,1418129_at)_Down,Mela(17276,1433438_x_at)_Down,Fam167b(230766,1455587_at)_Down,Mela(17276,1456182_x_at)_Down})=46.50
43	i({Mela(17276,1433438_x_at)_Down,0610011L14Rik(68295,1437835_a_at)_Down,Fam167b(230766,1455587_at)_Down,Mela(17276,1456182_x_at)_Down})=46.50
44	i({LOC433053(433053,1424931_s_at)_Down,Mela(17276,1433438_x_at)_Down,Fam167b(230766,1455587_at)_Down,Mela(17276,1456182_x_at)_Down})=46.50
45	i({Cntn1(12805,1452981_at)_Up,Mela(17276,1433438_x_at)_Down,Fam167b(230766,1455587_at)_Down,Mela(17276,1456182_x_at)_Down})=46.50
46	i({Osr2(107587,1426155_a_at)_Down,Mela(17276,1433438_x_at)_Down,Mela(17276,1456182_x_at)_Down,Mela(17276,1456456_x_at)_Down})=46.50
47	i({Mela(17276,1433438_x_at)_Down,Erlin1(226144,1441344_at)_Down,Mela(17276,1456182_x_at)_Down,Mela(17276,1456456_x_at)_Down})=46.50

48	i({Mela(17276,1433438_x_at)_Down,Slc25a13(50799,1449481_at)_Down,Mela(17276,1456182_x_at)_Down,Mela(17276,1456456_x_at)_Down})=46.50
49	i({Mela(17276,1433438_x_at)_Down,Dbp(13170,1438211_s_at)_Down,Fam167b(230766,1455587_at)_Down,Mela(17276,1456182_x_at)_Down})=46.50
50	i({Dhcr24(74754,1418129_at)_Down,Mela(17276,1433438_x_at)_Down,Fam167b(230766,1455587_at)_Down,Mela(17276,1456456_x_at)_Down})=46.50

Supplementary Table 14. Top-50 impactful itemsets of Air dataset in comparison 3 with a constant degree

Ranking	Impactful itemsets
1	i({Egr1(13653,1417065_at)_Down,Cxcl10(15945,1418930_at)_Down})=23.00
2	i({Creb1(12912,1452529_a_at)_Up,Egr1(13653,1417065_at)_Down})=22.00
3	i({Mre11a(17535,1416748_a_at)_Down,Egr1(13653,1417065_at)_Down})=20.50
4	i({Il1a(16175,1421473_at)_Up,Egr1(13653,1417065_at)_Down})=20.50
5	i({Egr1(13653,1417065_at)_Down,Vdr(22337,1418176_at)_Down})=20.50
6	i({Epas1(13819,1449888_at)_Up,Egr1(13653,1417065_at)_Down,Cxcl10(15945,1418930_at)_Down})=19.67
7	i({Steap3(68428,1453498_x_at)_Up,Egr1(13653,1417065_at)_Down,Cxcl10(15945,1418930_at)_Down})=19.67
8	i({Egr1(13653,1417065_at)_Down,Traf6(22034,1421377_at)_Down})=19.50
9	i({Egr1(13653,1417065_at)_Down,Slc2a2(20526,1449067_at)_Down})=19.50
10	i({Egr1(13653,1417065_at)_Down,Tnf(21926,1419607_at)_Down})=19.50
11	i({Egr1(13653,1417065_at)_Down,Rrm2(20135,1448226_at)_Down})=19.50
12	i({Creb1(12912,1452529_a_at)_Up,Egr1(13653,1417065_at)_Down,Vdr(22337,1418176_at)_Down})=19.33
13	i({Egr1(13653,1417065_at)_Down,Cxcl10(15945,1418930_at)_Down,Tnf(21926,1419607_at)_Down})=19.33
14	i({Egr1(13653,1417065_at)_Down,Vdr(22337,1418176_at)_Down,Cxcl10(15945,1418930_at)_Down})=19.33
15	i({Egr1(13653,1417065_at)_Down,Runx1(12394,1422864_at)_Down})=19.00
16	i({Foxo1(56458,1456529_at)_Up,Egr1(13653,1417065_at)_Down})=19.00
17	i({Cxcl11(56066,1419697_at)_Up,Egr1(13653,1417065_at)_Down})=19.00
18	i({Hnf1b(21410,1421224_a_at)_Up,Egr1(13653,1417065_at)_Down})=19.00
19	i({Pck1(18534,1455209_at)_Up,Egr1(13653,1417065_at)_Down})=19.00
20	i({Egr1(13653,1417065_at)_Down,Cxcl10(15945,1418930_at)_Down,Socs3(12702,1455899_x_at)_Down})=19.00
21	i({Wnt1(22408,1425377_at)_Up,Egr1(13653,1417065_at)_Down,Cxcl10(15945,1418930_at)_Down})=19.00
22	i({Slc8a1(20541,1425817_a_at)_Up,Egr1(13653,1417065_at)_Down,Cxcl10(15945,1418930_at)_Down})=19.00
23	i({Egr1(13653,1417065_at)_Down,Cxcl10(15945,1418930_at)_Down,Sos1(20662,1421886_at)_Down})=19.00
24	i({Epas1(13819,1449888_at)_Up,Creb1(12912,1452529_a_at)_Up,Egr1(13653,1417065_at)_Down})=19.00
25	i({Ppard(19015,1425703_at)_Up,Egr1(13653,1417065_at)_Down,Cxcl10(15945,1418930_at)_Down})=19.00
26	i({Foxo1(56458,1456529_at)_Up,Egr1(13653,1417065_at)_Down,Cxcl10(15945,1418930_at)_Down})=18.67
27	i({Cxcl11(56066,1419697_at)_Up,Egr1(13653,1417065_at)_Down,Cxcl10(15945,1418930_at)_Down})=18.67
28	i({Slc8a1(20541,1440201_at)_Up,Egr1(13653,1417065_at)_Down,Cxcl10(15945,1418930_at)_Down})=18.67
29	i({Egr1(13653,1417065_at)_Down,Cxcl10(15945,1418930_at)_Down,Mapk10(26414,1437195_x_at)_Down})=18.67
30	i({Creb1(12912,1452529_a_at)_Up,Egr1(13653,1417065_at)_Down,Per2(18627,1417602_at)_Down})=18.67
31	i({Mre11a(17535,1416748_a_at)_Down,Egr1(13653,1417065_at)_Down,Vdr(22337,1418176_at)_Down})=18.67
32	i({Creb1(12912,1452529_a_at)_Up,Egr1(13653,1417065_at)_Down,Sos1(20662,1421886_at)_Down})=18.67
33	i({Egr1(13653,1417065_at)_Down,Cxcl10(15945,1418930_at)_Down,Slc2a2(20526,1449067_at)_Down})=18.67
34	i({Cxcl11(56066,1419698_at)_Up,Egr1(13653,1417065_at)_Down,Cxcl10(15945,1418930_at)_Down})=18.67
35	i({Ifng(15978,1425947_at)_Up,Egr1(13653,1417065_at)_Down})=18.50
36	i({Egr1(13653,1417065_at)_Down,Socs3(12702,1455899_x_at)_Down})=18.50
37	i({Egr1(13653,1417065_at)_Down,Cxcl10(15945,1418930_at)_Down,Bai1(107831,1455363_at)_Down})=18.33
38	i({Vdr(22337,1418175_at)_Up,Egr1(13653,1417065_at)_Down,Cxcl10(15945,1418930_at)_Down})=18.33
39	i({Egr1(13653,1417065_at)_Down,Vdr(22337,1418176_at)_Down,Rrm2(20135,1448226_at)_Down})=18.33

40	i({Creb1(12912,1452529_a_at)_Up,Egr1(13653,1417065_at)_Down,Cxcl10(15945,1418930_at)_Down})=18.33
41	i({Creb1(12912,1452529_a_at)_Up,Egr1(13653,1417065_at)_Down,Slc2a2(20526,1449067_at)_Down})=18.33
42	i({Egr1(13653,1417065_at)_Down,Cxcl10(15945,1418930_at)_Down,Trp73(22062,1452325_at)_Down})=18.33
43	i({Il1a(16175,1421473_at)_Up,Egr1(13653,1417065_at)_Down,Cxcl10(15945,1418930_at)_Down})=18.33
44	i({Bcl2l1(12048,1426050_at)_Up,Egr1(13653,1417065_at)_Down,Cxcl10(15945,1418930_at)_Down})=18.33
45	i({Egr1(13653,1417065_at)_Down,Cxcl10(15945,1418930_at)_Down,Ifna1(15962,1450593_at)_Down})=18.33
46	i({Cxcl11(56066,1419698_at)_Up,Creb1(12912,1452529_a_at)_Up,Egr1(13653,1417065_at)_Down})=18.33
47	i({Gck(103988,1425303_at)_Up,Egr1(13653,1417065_at)_Down,Cxcl10(15945,1418930_at)_Down})=18.33
48	i({Csnk1e(27373,1438808_at)_Up,Egr1(13653,1417065_at)_Down,Cxcl10(15945,1418930_at)_Down})=18.33
49	i({Esr1(13982,1421244_at)_Up,Egr1(13653,1417065_at)_Down,Cxcl10(15945,1418930_at)_Down})=18.00
50	i({Creb1(12912,1452529_a_at)_Up,Egr1(13653,1417065_at)_Down,Hnf4g(30942,1450518_at)_Down})=18.00

Supplementary Table 15. Top-50 impactful itemsets of Air dataset in comparison 3 with various degrees

Ranking	Impactful itemsets
1	i({Egr1(13653,1417065_at)_Down,Ccnd1(12443,1417420_at)_Down,Tnf(21926,1419607_at)_Down})=73.33
2	i({Egr1(13653,1417065_at)_Down,Tnf(21926,1419607_at)_Down})=71.50
3	i({Myc(17869,1424942_a_at)_Down,Apc(11789,1450056_at)_Down})=70.50
4	i({Egr1(13653,1417065_at)_Down,Myc(17869,1424942_a_at)_Down})=68.00
5	i({Egr1(13653,1417065_at)_Down,Tnf(21926,1419607_at)_Down,Apc(11789,1450056_at)_Down})=67.00
6	i({Pax6(18508,1452526_a_at)_Up,Myc(17869,1424942_a_at)_Down,Apc(11789,1450056_at)_Down})=67.00
7	i({Egr1(13653,1417065_at)_Down,Tnf(21926,1419607_at)_Down,Trp53(22059,1426538_a_at)_Down})=67.00
8	i({Egr1(13653,1417065_at)_Down,Tnf(21926,1419607_at)_Down,Myc(17869,1424942_a_at)_Down})=65.67
9	i({Ccnd1(12443,1417420_at)_Down,Vdr(22337,1418176_at)_Down,Tnf(21926,1419607_at)_Down})=64.00
10	i({Ifng(15978,1425947_at)_Up,Myc(17869,1424942_a_at)_Down,Apc(11789,1450056_at)_Down})=63.67
11	i({Mapk10(26414,1437195_x_at)_Down,Apc(11789,1450056_at)_Down})=63.50
12	i({Vdr(22337,1418176_at)_Down,Myc(17869,1424942_a_at)_Down,Apc(11789,1450056_at)_Down})=63.00
13	i({Epas1(13819,1449888_at)_Up,Egr1(13653,1417065_at)_Down,Tnf(21926,1419607_at)_Down})=62.33
14	i({Pax6(18508,1437816_at)_Up,Myc(17869,1424942_a_at)_Down,Apc(11789,1450056_at)_Down})=62.33
15	i({Egr1(13653,1417065_at)_Down,Vdr(22337,1418176_at)_Down,Apc(11789,1450056_at)_Down})=61.67
16	i({Ccnd1(12443,1417419_at)_Down,Myc(17869,1424942_a_at)_Down,Apc(11789,1450056_at)_Down})=61.67
17	i({Pklr(18770,1421259_at)_Up,Myc(17869,1424942_a_at)_Down,Apc(11789,1450056_at)_Down})=61.33
18	i({Cdh2(12558,1418815_at)_Up,Myc(17869,1424942_a_at)_Down,Apc(11789,1450056_at)_Down})=61.33
19	i({Sfrp1(20377,1438620_x_at)_Up,Myc(17869,1424942_a_at)_Down,Apc(11789,1450056_at)_Down})=61.00
20	i({Myc(17869,1424942_a_at)_Down,Mapk10(26414,1437195_x_at)_Down,Apc(11789,1450056_at)_Down})=60.33
21	i({Egr1(13653,1417065_at)_Down,Ccnd1(12443,1417419_at)_Down,Myc(17869,1424942_a_at)_Down})=60.00
22	i({Pdx1(18609,1422173_at)_Up,Ccnd1(12443,1417420_at)_Down,Tnf(21926,1419607_at)_Down})=60.00
23	i({Pax6(18508,1437816_at)_Up,Ccnd1(12443,1417420_at)_Down,Tnf(21926,1419607_at)_Down})=59.67
24	i({Wnt1(22408,1425377_at)_Up,Apc(11789,1450056_at)_Down})=59.50
25	i({Egr1(13653,1417065_at)_Down,Tnf(21926,1419607_at)_Down,Mapk10(26414,1437195_x_at)_Down})=59.33
26	i({Egr1(13653,1417065_at)_Down,Mapk1(26413,1419568_at)_Down,Tnf(21926,1419607_at)_Down})=59.33
27	i({Il1a(16175,1421473_at)_Up,Ccnd1(12443,1417420_at)_Down,Tnf(21926,1419607_at)_Down})=59.00
28	i({Vdr(22337,1418175_at)_Up,Ccnd1(12443,1417420_at)_Down,Tnf(21926,1419607_at)_Down})=59.00
29	i({Egr1(13653,1417065_at)_Down,Myc(17869,1424942_a_at)_Down,Mapk10(26414,1437195_x_at)_Down})=58.67
30	i({Tnf(21926,1419607_at)_Down,Myc(17869,1424942_a_at)_Down,Apc(11789,1450056_at)_Down})=58.67
31	i({Mre11a(17535,1416748_a_at)_Down,Ccnd1(12443,1417420_at)_Down,Tnf(21926,1419607_at)_Down})=58.67
32	i({Egr1(13653,1417065_at)_Down,Tnf(21926,1419607_at)_Down,Fos(14281,1423100_at)_Down})=58.33
33	i({Egr1(13653,1417065_at)_Down,Ccnd1(12443,1417420_at)_Down,Vdr(22337,1418176_at)_Down})=58.33
34	i({Il1a(16175,1421473_at)_Up,Egr1(13653,1417065_at)_Down,Tnf(21926,1419607_at)_Down})=57.67
35	i({Vdr(22337,1418175_at)_Up,Egr1(13653,1417065_at)_Down,Tnf(21926,1419607_at)_Down})=57.67
36	i({Wnt1(22408,1425377_at)_Up,Myc(17869,1424942_a_at)_Down,Apc(11789,1450056_at)_Down})=57.67
37	i({Vdr(22337,1418176_at)_Down,Tnf(21926,1419607_at)_Down,Trp53(22059,1426538_a_at)_Down})=57.67
38	i({Egr1(13653,1417065_at)_Down,Tnf(21926,1419607_at)_Down,Sos1(20662,1421886_at)_Down})=57.67
39	i({Vdr(22337,1418176_at)_Down,Tnf(21926,1419607_at)_Down,Apc(11789,1450056_at)_Down})=57.67

40	i({Egr1(13653,1417065_at)_Down,Apc(11789,1450056_at)_Down})=57.50
41	i({Vdr(22337,1418176_at)_Down,Tnf(21926,1419607_at)_Down})=57.50
42	i({Egr1(13653,1417065_at)_Down,Tnf(21926,1419607_at)_Down,Apc(11789,1420957_at)_Down})=57.33
43	i({Pdx1(18609,1422173_at)_Up,Tnf(21926,1419607_at)_Down,Apc(11789,1450056_at)_Down})=57.33
44	i({Apc(11789,1420956_at)_Up,Egr1(13653,1417065_at)_Down,Tnf(21926,1419607_at)_Down})=57.33
45	i({Pdx1(18609,1422173_at)_Up,Tnf(21926,1419607_at)_Down,Trp53(22059,1426538_a_at)_Down})=57.33
46	i({Dkk1(13380,1420360_at)_Up,Egr1(13653,1417065_at)_Down,Tnf(21926,1419607_at)_Down})=57.00
47	i({Wnt1(22408,1425377_at)_Up,Egr1(13653,1417065_at)_Down,Tnf(21926,1419607_at)_Down})=57.00
48	i({Nkx2-2(18088,1421112_at)_Up,Myc(17869,1424942_a_at)_Down,Apc(11789,1450056_at)_Down})=57.00
49	i({Pax6(18508,1452526_a_at)_Up,Myc(17869,1424942_a_at)_Down})=57.00
50	i({Pdx1(18609,1422173_at)_Up,Tnf(21926,1419607_at)_Down})=57.00

Supplementary Table 16. Top-50 impactful itemsets of Phosgene dataset in comparison 3 of undifferentiation control method

Ranking	Impactful itemsets
1	i({Mt2(17750,1428942_at)_Up,Atf3(11910,1449363_at)_Up})=46.00
2	i({Mt1(17748,1422557_s_at)_Up,Mt2(17750,1428942_at)_Up,Atf3(11910,1449363_at)_Up})=45.00
3	i({Tnfrsf12a(27279,1418571_at)_Up,Mt2(17750,1428942_at)_Up,Atf3(11910,1449363_at)_Up})=44.67
4	i({Mt1(17748,1422557_s_at)_Up,Mt2(17750,1428942_at)_Up})=44.50
5	i({Mt1(17748,1422557_s_at)_Up,Atf3(11910,1449363_at)_Up})=44.50
6	i({Tnfrsf12a(27279,1418572_x_at)_Up,Mt2(17750,1428942_at)_Up,Atf3(11910,1449363_at)_Up})=44.33
7	i({Tnfrsf12a(27279,1418571_at)_Up,Mt1(17748,1422557_s_at)_Up,Mt2(17750,1428942_at)_Up,Atf3(11910,1449363_at)_Up})=44.25
8	i({Tnfrsf12a(27279,1418571_at)_Up,Mt2(17750,1428942_at)_Up})=44.00
9	i({Tnfrsf12a(27279,1418571_at)_Up,Atf3(11910,1449363_at)_Up})=44.00
10	i({Tnfrsf12a(27279,1418572_x_at)_Up,Mt1(17748,1422557_s_at)_Up,Mt2(17750,1428942_at)_Up,Atf3(11910,1449363_at)_Up})=44.00
11	i({Tnfrsf12a(27279,1418571_at)_Up,Tnfrsf12a(27279,1418572_x_at)_Up,Mt2(17750,1428942_at)_Up,Atf3(11910,1449363_at)_Up})=43.75
12	i({Tnfrsf12a(27279,1418571_at)_Up,Mt1(17748,1422557_s_at)_Up,Mt2(17750,1428942_at)_Up})=43.67
13	i({Tnfrsf12a(27279,1418571_at)_Up,Mt1(17748,1422557_s_at)_Up,Atf3(11910,1449363_at)_Up})=43.67
14	i({Tnfrsf12a(27279,1418571_at)_Up,Tnfrsf12a(27279,1418572_x_at)_Up,Mt1(17748,1422557_s_at)_Up,Mt2(17750,1428942_at)_Up,Atf3(11910,1449363_at)_Up})=43.60
15	i({Tnfrsf12a(27279,1418572_x_at)_Up,Mt2(17750,1428942_at)_Up})=43.50
16	i({Tnfrsf12a(27279,1418572_x_at)_Up,Atf3(11910,1449363_at)_Up})=43.50
17	i({Krt8(16691,1423691_x_at)_Up,Mt2(17750,1428942_at)_Up,Atf3(11910,1449363_at)_Up})=43.33
18	i({Tnfrsf12a(27279,1418572_x_at)_Up,Mt1(17748,1422557_s_at)_Up,Mt2(17750,1428942_at)_Up})=43.33
19	i({Tnfrsf12a(27279,1418572_x_at)_Up,Mt1(17748,1422557_s_at)_Up,Atf3(11910,1449363_at)_Up})=43.33
20	i({Srxn1(76650,1426875_s_at)_Up,Mt2(17750,1428942_at)_Up,Atf3(11910,1449363_at)_Up})=43.33
21	i({Mt1(17748,1422557_s_at)_Up,Srxn1(76650,1426875_s_at)_Up,Mt2(17750,1428942_at)_Up,Atf3(11910,1449363_at)_Up})=43.25
22	i({Mt1(17748,1422557_s_at)_Up,Krt8(16691,1423691_x_at)_Up,Mt2(17750,1428942_at)_Up,Atf3(11910,1449363_at)_Up})=43.25
23	i({Mt2(17750,1428942_at)_Up,Clu(12759,1437689_x_at)_Up,Atf3(11910,1449363_at)_Up})=43.00
24	i({Tnfrsf12a(27279,1418571_at)_Up,Srxn1(76650,1426875_s_at)_Up,Mt2(17750,1428942_at)_Up,Atf3(11910,1449363_at)_Up})=43.00
25	i({Mt1(17748,1422557_s_at)_Up,Mt2(17750,1428942_at)_Up,Clu(12759,1437689_x_at)_Up,Atf3(11910,1449363_at)_Up})=43.00
26	i({Tnfrsf12a(27279,1418571_at)_Up,Tnfrsf12a(27279,1418572_x_at)_Up,Mt1(17748,1422557_s_at)_Up,Mt2(17750,1428942_at)_Up})=43.00
27	i({Tnfrsf12a(27279,1418571_at)_Up,Tnfrsf12a(27279,1418572_x_at)_Up,Mt1(17748,1422557_s_at)_Up,Atf3(11910,1449363_at)_Up})=43.00
28	i({Tnfrsf12a(27279,1418571_at)_Up,Krt8(16691,1423691_x_at)_Up,Mt2(17750,1428942_at)_Up,Atf3(11910,1449363_at)_Up})=43.00
29	i({Tnfrsf12a(27279,1418571_at)_Up,Mt1(17748,1422557_s_at)_Up,Srxn1(76650,1426875_s_at)_Up,Mt2(17750,1428942_at)_Up,Atf3(11910,1449363_at)_Up})=43.00
30	i({Tnfrsf12a(27279,1418571_at)_Up,Mt1(17748,1422557_s_at)_Up,Krt8(16691,1423691_x_at)_Up,Mt2(17750,1428942_at)_Up,Atf3(11910,1449363_at)_Up})=43.00
31	i({Tnfrsf12a(27279,1418571_at)_Up,Tnfrsf12a(27279,1418572_x_at)_Up,Mt2(17750,1428942_at)_Up})=43.00
32	i({Tnfrsf12a(27279,1418571_at)_Up,Tnfrsf12a(27279,1418572_x_at)_Up,Atf3(11910,1449363_at)_Up})=43.00
33	i({Tnfrsf12a(27279,1418572_x_at)_Up,Mt1(17748,1422557_s_at)_Up,Krt8(16691,1423691_x_at)_Up,Mt2(17750,1428942_at)_Up,Atf3(11910,1449363_at)_Up})=42.80

34	i({Tnfrsf12a(27279,1418571_at)_Up,Mt1(17748,1422557_s_at)_Up,Mt2(17750,1428942_at)_Up,Clu(12759,1437689_x_at)_Up,Atf3(11910,1449363_at)_Up})=42.80
35	i({Tnfrsf12a(27279,1418572_x_at)_Up,Mt1(17748,1422557_s_at)_Up,Srxn1(76650,1426875_s_at)_Up,Mt2(17750,1428942_at)_Up,Atf3(11910,1449363_at)_Up})=42.80
36	i({Mt1(17748,1422557_s_at)_Up,Mt2(17750,1428942_at)_Up,Slc2a1(20525,1434773_a_at)_Up,Atf3(11910,1449363_at)_Up})=42.75
37	i({Tnfrsf12a(27279,1418572_x_at)_Up,Srxn1(76650,1426875_s_at)_Up,Mt2(17750,1428942_at)_Up,Atf3(11910,1449363_at)_Up})=42.75
38	i({Tnfrsf12a(27279,1418571_at)_Up,Mt2(17750,1428942_at)_Up,Clu(12759,1437689_x_at)_Up,Atf3(11910,1449363_at)_Up})=42.75
39	i({Mt1(17748,1422557_s_at)_Up,Mt2(17750,1428942_at)_Up,Atf3(11910,1449363_at)_Up,Timp1(21857,1460227_at)_Up})=42.75
40	i({Tnfrsf12a(27279,1418572_x_at)_Up,Krt8(16691,1423691_x_at)_Up,Mt2(17750,1428942_at)_Up,Atf3(11910,1449363_at)_Up})=42.75
41	i({Mt2(17750,1428942_at)_Up,Slc2a1(20525,1434773_a_at)_Up,Atf3(11910,1449363_at)_Up})=42.67
42	i({Mt2(17750,1428942_at)_Up,Atf3(11910,1449363_at)_Up,Timp1(21857,1460227_at)_Up})=42.67
43	i({Tnfrsf12a(27279,1418571_at)_Up,Tnfrsf12a(27279,1418572_x_at)_Up,Mt1(17748,1422557_s_at)_Up,Srxn1(76650,1426875_s_at)_Up,Mt2(17750,1428942_at)_Up,Atf3(11910,1449363_at)_Up})=42.67
44	i({Tnfrsf12a(27279,1418571_at)_Up,Tnfrsf12a(27279,1418572_x_at)_Up,Mt1(17748,1422557_s_at)_Up,Krt8(16691,1423691_x_at)_Up,Mt2(17750,1428942_at)_Up,Atf3(11910,1449363_at)_Up})=42.67
45	i({Tnfrsf12a(27279,1418571_at)_Up,Mt1(17748,1422557_s_at)_Up,Mt2(17750,1428942_at)_Up,Slc2a1(20525,1434773_a_at)_Up,Atf3(11910,1449363_at)_Up})=42.60
46	i({Tnfrsf12a(27279,1418571_at)_Up,Mt1(17748,1422557_s_at)_Up,Mt2(17750,1428942_at)_Up,Atf3(11910,1449363_at)_Up,Timp1(21857,1460227_at)_Up})=42.60
47	i({Tnfrsf12a(27279,1418571_at)_Up,Tnfrsf12a(27279,1418572_x_at)_Up,Krt8(16691,1423691_x_at)_Up,Mt2(17750,1428942_at)_Up,Atf3(11910,1449363_at)_Up})=42.60
48	i({Tnfrsf12a(27279,1418571_at)_Up,Tnfrsf12a(27279,1418572_x_at)_Up,Srxn1(76650,1426875_s_at)_Up,Mt2(17750,1428942_at)_Up,Atf3(11910,1449363_at)_Up})=42.60
49	i({Tnfrsf12a(27279,1418571_at)_Up,Mt1(17748,1422557_s_at)_Up})=42.50
50	i({Mt1(17748,1422557_s_at)_Up,Mt2(17750,1428942_at)_Up,Atf3(11910,1449363_at)_Up,Spns2(216892,1451601_a_at)_Down})=42.50

Supplementary Table 17. Top-50 impactful itemsets of Phosgene dataset in comparison 3 with a constant degree

Ranking	Impactful itemsets
1	i({Myc(17869,1424942_a_at)_Up,Slc2a1(20525,1434773_a_at)_Up})=37.50
2	i({Myc(17869,1424942_a_at)_Up,Socs3(12702,1456212_x_at)_Up})=37.00
3	i({Myc(17869,1424942_a_at)_Up,Slc2a1(20525,1434773_a_at)_Up,Socs3(12702,1456212_x_at)_Up})=36.67
4	i({Myc(17869,1424942_a_at)_Up,Prickle1(106042,1452249_at)_Down})=36.50
5	i({Myc(17869,1424942_a_at)_Up,Slc2a1(20525,1434773_a_at)_Up,Prickle1(106042,1452249_at)_Down})=36.33
6	i({Myc(17869,1424942_a_at)_Up,Socs3(12702,1456212_x_at)_Up,Prickle1(106042,1452249_at)_Down})=36.00
7	i({Myc(17869,1424942_a_at)_Up,Slc2a1(20525,1434773_a_at)_Up,Socs3(12702,1456212_x_at)_Up,Prickle1(106042,1452249_at)_Down})=36.00
8	i({Myc(17869,1424942_a_at)_Up,Slc2a1(20525,1434773_a_at)_Up,Socs3(12702,1455899_x_at)_Up,Prickle1(106042,1452249_at)_Down})=35.75
9	i({Cdkn1a(12575,1424638_at)_Up,Myc(17869,1424942_a_at)_Up,Slc2a1(20525,1434773_a_at)_Up})=35.67
10	i({Myc(17869,1424942_a_at)_Up,Socs3(12702,1455899_x_at)_Up,Prickle1(106042,1452249_at)_Down})=35.67
11	i({Myc(17869,1424942_a_at)_Up,Slc2a1(20525,1434773_a_at)_Up,Socs3(12702,1455899_x_at)_Up,Socs3(12702,1456212_x_at)_Up,Prickle1(106042,1452249_at)_Down})=35.60
12	i({Slc2a1(20525,1434773_a_at)_Up,Socs3(12702,1456212_x_at)_Up})=35.50
13	i({Cdkn1a(12575,1424638_at)_Up,Myc(17869,1424942_a_at)_Up})=35.50
14	i({Cdkn1a(12575,1424638_at)_Up,Myc(17869,1424942_a_at)_Up,Slc2a1(20525,1434773_a_at)_Up,Socs3(12702,1456212_x_at)_Up})=35.50
15	i({Myc(17869,1424942_a_at)_Up,Socs3(12702,1455899_x_at)_Up,Socs3(12702,1456212_x_at)_Up,Prickle1(106042,1452249_at)_Down})=35.50
16	i({Cdkn1a(12575,1424638_at)_Up,Myc(17869,1424942_a_at)_Up,Socs3(12702,1456212_x_at)_Up})=35.33
17	i({Cdkn1a(12575,1424638_at)_Up,Myc(17869,1424942_a_at)_Up,Slc2a1(20525,1434773_a_at)_Up,Prickle1(106042,1452249_at)_Down})=35.25
18	i({Myc(17869,1424942_a_at)_Up,Slc2a1(20525,1426599_a_at)_Up,Slc2a1(20525,1434773_a_at)_Up,Prickle1(106042,1452249_at)_Down})=35.25
19	i({Cdkn1a(12575,1424638_at)_Up,Myc(17869,1424942_a_at)_Up,Slc2a1(20525,1434773_a_at)_Up,Socs3(12702,1455899_x_at)_Up})=35.25
20	i({Cdkn1a(12575,1424638_at)_Up,Myc(17869,1424942_a_at)_Up,Slc2a1(20525,1434773_a_at)_Up,Socs3(12702,1456212_x_at)_Up,Prickle1(106042,1452249_at)_Down})=35.20
21	i({Cdkn1a(12575,1424638_at)_Up,Myc(17869,1424942_a_at)_Up,Slc2a1(20525,1434773_a_at)_Up,Socs3(12702,1455899_x_at)_Up,Socs3(12702,1456212_x_at)_Up})=35.20
22	i({Myc(17869,1424942_a_at)_Up,Slc2a1(20525,1426599_a_at)_Up,Slc2a1(20525,1434773_a_at)_Up,Socs3(12702,1456212_x_at)_Up,Prickle1(106042,1452249_at)_Down})=35.20
23	i({Slc2a1(20525,1434773_a_at)_Up,Prickle1(106042,1452249_at)_Down})=35.00
24	i({Cdkn1a(12575,1424638_at)_Up,Myc(17869,1424942_a_at)_Up,Prickle1(106042,1452249_at)_Down})=35.00
25	i({Slc2a1(20525,1434773_a_at)_Up,Socs3(12702,1456212_x_at)_Up,Prickle1(106042,1452249_at)_Down})=35.00
26	i({Cdkn1a(12575,1424638_at)_Up,Myc(17869,1424942_a_at)_Up,Socs3(12702,1456212_x_at)_Up,Prickle1(106042,1452249_at)_Down})=35.00
27	i({Myc(17869,1424942_a_at)_Up,Slc2a1(20525,1426599_a_at)_Up,Socs3(12702,1456212_x_at)_Up,Prickle1(106042,1452249_at)_Down})=35.00
28	i({Cdkn1a(12575,1424638_at)_Up,Myc(17869,1424942_a_at)_Up,Socs3(12702,1455899_x_at)_Up,Socs3(12702,1456212_x_at)_Up})=35.00

29	i({Cdkn1a(12575,1424638_at)_Up,Myc(17869,1424942_a_at)_Up,Slc2a1(20525,1434773_a_at)_Up,Socs3(12702,1455899_x_at)_Up,Prickle1(1 0 6042,1452249_at)_Down})=35.00
30	i({Cdkn1a(12575,1424638_at)_Up,Myc(17869,1424942_a_at)_Up,Slc2a1(20525,1434773_a_at)_Up,Socs3(12702,1455899_x_at)_Up,Socs3(127 02,1456212_x_at)_Up,Prickle1(106042,1452249_at)_Down})=35.00
31	i({Cdkn1a(12575,1424638_at)_Up,Myc(17869,1424942_a_at)_Up,Slc2a1(20525,1426599_a_at)_Up,Slc2a1(20525,1434773_a_at)_Up,Socs3(12 702,1456212_x_at)_Up})=34.80
32	i({Cdkn1a(12575,1424638_at)_Up,Myc(17869,1424942_a_at)_Up,Socs3(12702,1455899_x_at)_Up,Socs3(12702,1456212_x_at)_Up,Prickle1(10 6042,1452249_at)_Down})=34.80
33	i({Cdkn1a(12575,1424638_at)_Up,Myc(17869,1424942_a_at)_Up,Slc2a1(20525,1426599_a_at)_Up,Slc2a1(20525,1434773_a_at)_Up})=34.75
34	i({Cdkn1a(12575,1424638_at)_Up,Myc(17869,1424942_a_at)_Up,Socs3(12702,1455899_x_at)_Up,Prickle1(106042,1452249_at)_Down})=34.7 5
35	i({Slc2a1(20525,1434773_a_at)_Up,Socs3(12702,1455899_x_at)_Up,Socs3(12702,1456212_x_at)_Up,Prickle1(106042,1452249_at)_Down})=3 4.75
36	i({Cdkn1a(12575,1424638_at)_Up,Myc(17869,1424942_a_at)_Up,Slc2a1(20525,1426599_a_at)_Up,Slc2a1(20525,1434773_a_at)_Up,Socs3(12 702,1456212_x_at)_Up,Prickle1(106042,1452249_at)_Down})=34.67
37	i({Slc2a1(20525,1434773_a_at)_Up,Socs3(12702,1455899_x_at)_Up,Prickle1(106042,1452249_at)_Down})=34.67
38	i({Cdkn1a(12575,1424638_at)_Up,Myc(17869,1424942_a_at)_Up,Slc2a1(20525,1426599_a_at)_Up,Slc2a1(20525,1434773_a_at)_Up,Prickle1(106042,1452249_at)_Down})=34.60
39	i({Myc(17869,1424942_a_at)_Up,Slc2a1(20525,1426599_a_at)_Up})=34.50
40	i({Socs3(12702,1456212_x_at)_Up,Prickle1(106042,1452249_at)_Down})=34.50
41	i({Cdkn1a(12575,1424638_at)_Up,Myc(17869,1424942_a_at)_Up,Slc2a1(20525,1426599_a_at)_Up,Socs3(12702,1456212_x_at)_Up})=34.50
42	i({Cdkn1a(12575,1424638_at)_Up,Myc(17869,1424942_a_at)_Up,Slc2a1(20525,1426599_a_at)_Up,Socs3(12702,1456212_x_at)_Up,Prickle1(1 06042,1452249_at)_Down})=34.40
43	i({Myc(17869,1424942_a_at)_Up,Slc2a1(20525,1426599_a_at)_Up,Slc2a1(20525,1434773_a_at)_Up})=34.33
44	i({Cdkn1a(12575,1424638_at)_Up,Slc2a1(20525,1434773_a_at)_Up,Socs3(12702,1456212_x_at)_Up})=34.33
45	i({Socs3(12702,1455899_x_at)_Up,Socs3(12702,1456212_x_at)_Up,Prickle1(106042,1452249_at)_Down})=34.33
46	i({Cdkn1a(12575,1424638_at)_Up,Slc2a1(20525,1434773_a_at)_Up,Socs3(12702,1456212_x_at)_Up,Prickle1(106042,1452249_at)_Down})=34 .25
47	i({Cdkn1a(12575,1424638_at)_Up,Myc(17869,1424942_a_at)_Up,Slc2a1(20525,1426599_a_at)_Up,Prickle1(106042,1452249_at)_Down})=34. 25
48	i({Slc2a1(20525,1426599_a_at)_Up,Slc2a1(20525,1434773_a_at)_Up,Socs3(12702,1456212_x_at)_Up,Prickle1(106042,1452249_at)_Down})= 34.25
49	i({Cdkn1a(12575,1424638_at)_Up,Slc2a1(20525,1434773_a_at)_Up,Socs3(12702,1455899_x_at)_Up,Socs3(12702,1456212_x_at)_Up})=34.25
50	i({Cdkn1a(12575,1424638_at)_Up,Slc2a1(20525,1434773_a_at)_Up,Socs3(12702,1455899_x_at)_Up,Socs3(12702,1456212_x_at)_Up,Prickle1(106042,1452249_at)_Down})=34.20

Supplementary Table 18. Top-50 impactful itemsets of Phosgene dataset in comparison 3 with various degrees

Ranking	Impactful itemsets
1	i({Myc(17869,1424942_a_at)_Up,Socs3(12702,1456212_x_at)_Up})=228.00
2	i({Apc(11789,1420957_at)_Up,Myc(17869,1424942_a_at)_Up,Socs3(12702,1456212_x_at)_Up})=210.00
3	i({Myc(17869,1424942_a_at)_Up,Socs3(12702,1455899_x_at)_Up})=204.00
4	i({Myc(17869,1424942_a_at)_Up,Socs3(12702,1456212_x_at)_Up,Trp53(22059,1426538_a_at)_Down})=200.33
5	i({Myc(17869,1424942_a_at)_Up,Socs3(12702,1456212_x_at)_Up,Apc(11789,1450056_at)_Down})=200.33
6	i({Myc(17869,1424942_a_at)_Up,Il6(16193,1450297_at)_Up})=196.00
7	i({Apc(11789,1420957_at)_Up,Myc(17869,1424942_a_at)_Up,Socs3(12702,1455899_x_at)_Up})=194.00
8	i({Myc(17869,1424942_a_at)_Up,Slc2a1(20525,1434773_a_at)_Up})=193.50
9	i({Myc(17869,1424942_a_at)_Up,Prickle1(106042,1452249_at)_Down})=192.50
10	i({Socs3(12702,1416576_at)_Up,Myc(17869,1424942_a_at)_Up})=192.00
11	i({Cdkn1a(12575,1424638_at)_Up,Myc(17869,1424942_a_at)_Up})=191.50
12	i({Myc(17869,1424942_a_at)_Up,Sufu(24069,1450024_at)_Down})=188.50
13	i({Myc(17869,1424942_a_at)_Up,Igfbp3(16009,1423062_at)_Down})=188.00
14	i({Myc(17869,1424942_a_at)_Up,Socs3(12702,1456212_x_at)_Up,Ctnnb1(12387,1430533_a_at)_Down})=188.00
15	i({Fos(14281,1423100_at)_Up,Myc(17869,1424942_a_at)_Up})=187.50
16	i({Myc(17869,1424942_a_at)_Up,Thbs1(21825,1450377_at)_Up})=187.50
17	i({Apc(11789,1420957_at)_Up,Myc(17869,1424942_a_at)_Up,Slc2a1(20525,1434773_a_at)_Up})=187.00
18	i({Apc(11789,1420957_at)_Up,Myc(17869,1424942_a_at)_Up,Prickle1(106042,1452249_at)_Down})=186.33
19	i({Socs3(12702,1416576_at)_Up,Apc(11789,1420957_at)_Up,Myc(17869,1424942_a_at)_Up})=186.00
20	i({Apc(11789,1420957_at)_Up,Cdkn1a(12575,1424638_at)_Up,Myc(17869,1424942_a_at)_Up})=185.67
21	i({Myc(17869,1424942_a_at)_Up,Socs3(12702,1455899_x_at)_Up,Apc(11789,1450056_at)_Down})=184.33
22	i({Fos(14281,1423100_at)_Up,Myc(17869,1424942_a_at)_Up,Socs3(12702,1456212_x_at)_Up})=184.00
23	i({Myc(17869,1424942_a_at)_Up,Figf(14205,1438953_at)_Up,Socs3(12702,1456212_x_at)_Up})=184.00
24	i({Apc(11789,1420957_at)_Up,Myc(17869,1424942_a_at)_Up,Sufu(24069,1450024_at)_Down})=183.67
25	i({Myc(17869,1424942_a_at)_Up,Vcam1(22329,1415989_at)_Down})=183.50
26	i({Apc(11789,1420957_at)_Up,Myc(17869,1424942_a_at)_Up,Igfbp3(16009,1423062_at)_Down})=183.33
27	i({Apc(11789,1420957_at)_Up,Myc(17869,1424942_a_at)_Up,Thbs1(21825,1450377_at)_Up})=183.00
28	i({Myc(17869,1424942_a_at)_Up,Slc2a1(20525,1426599_a_at)_Up})=182.50
29	i({Myc(17869,1424942_a_at)_Up,Socs3(12702,1456212_x_at)_Up,Lef1(16842,1421299_a_at)_Down})=182.00
30	i({Apc(11789,1420957_at)_Up,Myc(17869,1424942_a_at)_Up})=181.50
31	i({Apc(11789,1420957_at)_Up,Myc(17869,1424942_a_at)_Up,Vcam1(22329,1415989_at)_Down})=180.33
32	i({Apc(11789,1420957_at)_Up,Myc(17869,1424942_a_at)_Up,Slc2a1(20525,1426599_a_at)_Up})=179.67
33	i({Serpine1(18787,1419149_at)_Up,Myc(17869,1424942_a_at)_Up})=179.50
34	i({Apc(11789,1420957_at)_Up,Myc(17869,1424942_a_at)_Up,Il6(16193,1450297_at)_Up})=179.00
35	i({Serpine1(18787,1419149_at)_Up,Apc(11789,1420957_at)_Up,Myc(17869,1424942_a_at)_Up})=177.67
36	i({Myc(17869,1424942_a_at)_Up,Slc2a1(20525,1434773_a_at)_Up,Apc(11789,1450056_at)_Down})=177.33
37	i({Myc(17869,1424942_a_at)_Up,Slc2a1(20525,1434773_a_at)_Up,Trp53(22059,1426538_a_at)_Down})=177.33
38	i({Myc(17869,1424942_a_at)_Up,Agrn(11603,1426670_at)_Down})=177.00
39	i({Myc(17869,1424942_a_at)_Up,Trp53(22059,1426538_a_at)_Down,Prickle1(106042,1452249_at)_Down})=176.67

40	i({Myc(17869,1424942_a_at)_Up,Apc(11789,1450056_at)_Down,Prickle1(106042,1452249_at)_Down})=176.67
41	i({Socs3(12702,1416576_at)_Up,Myc(17869,1424942_a_at)_Up,Apc(11789,1450056_at)_Down})=176.33
42	i({Myc(17869,1424942_a_at)_Up,Vangl2(93840,1419218_at)_Down})=176.00
43	i({Cdkn1a(12575,1424638_at)_Up,Myc(17869,1424942_a_at)_Up,Trp53(22059,1426538_a_at)_Down})=176.00
44	i({Cdkn1a(12575,1424638_at)_Up,Myc(17869,1424942_a_at)_Up,Apc(11789,1450056_at)_Down})=176.00
45	i({Egr1(13653,1417065_at)_Up,Myc(17869,1424942_a_at)_Up})=174.50
46	i({Myc(17869,1424942_a_at)_Up,Cebpb(12608,1427844_a_at)_Up})=174.00
47	i({Myc(17869,1424942_a_at)_Up,Trp53(22059,1426538_a_at)_Down,Sufu(24069,1450024_at)_Down})=174.00
48	i({Myc(17869,1424942_a_at)_Up,Sufu(24069,1450024_at)_Down,Apc(11789,1450056_at)_Down})=174.00
49	i({Apc(11789,1420957_at)_Up,Myc(17869,1424942_a_at)_Up,Cebpb(12608,1427844_a_at)_Up})=174.00
50	i({Jun(16476,1417409_at)_Up,Myc(17869,1424942_a_at)_Up,Socs3(12702,1456212_x_at)_Up})=173.67

Supplementary Table 19. Disassembled relations derived from Top-50 impactful itemsets of Wild type dataset in comparison 1 of undifferentiation control method

Disassembled Relations	References
ELMO1(9844)_Up,UBR1(197131)_Up	New
AJAP1(55966)_Up,UBR1(197131)_Up	New
ELMO1(9844)_Up,AJAP1(55966)_Up	New
CNOT4(4850)_Up,UBR1(197131)_Up	New
CNOT4(4850)_Up,ELMO1(9844)_Up	New
CNOT4(4850)_Up,AJAP1(55966)_Up	New
MLF2(8079)_Down,UBR1(197131)_Up	New
MLF2(8079)_Down,ELMO1(9844)_Up	New
MLF2(8079)_Down,AJAP1(55966)_Up	New
CNOT4(4850)_Up,MLF2(8079)_Down	New
ITGA8(8516)_Down,UBR1(197131)_Up	New
ITGA8(8516)_Down,ELMO1(9844)_Up	New
ITGA8(8516)_Down,AJAP1(55966)_Up	New
CNOT4(4850)_Up,ITGA8(8516)_Down	New
MLF2(8079)_Down,ITGA8(8516)_Down	New
UBR1(197131)_Up,CCDC111(201973)_Up	New
ELMO1(9844)_Up,CCDC111(201973)_Up	New
AJAP1(55966)_Up,CCDC111(201973)_Up	New
CNOT4(4850)_Up,CCDC111(201973)_Up	New
KLHL8(57563)_Down,UBR1(197131)_Up	New
ELMO1(9844)_Up,KLHL8(57563)_Down	New
AJAP1(55966)_Up,KLHL8(57563)_Down	New
CNOT4(4850)_Up,KLHL8(57563)_Down	New
SCRN2(90507)_Down,UBR1(197131)_Up	New
ELMO1(9844)_Up,SCRN2(90507)_Down	New
AJAP1(55966)_Up,SCRN2(90507)_Down	New
CNOT4(4850)_Up,SCRN2(90507)_Down	New
CALML4(91860)_Down,UBR1(197131)_Up	New
ELMO1(9844)_Up,CALML4(91860)_Down	New
AJAP1(55966)_Up,CALML4(91860)_Down	New
CNOT4(4850)_Up,CALML4(91860)_Down	New
PDXDC1(23042)_Down,UBR1(197131)_Up	New
ELMO1(9844)_Up,PDXDC1(23042)_Down	New
PDXDC1(23042)_Down,AJAP1(55966)_Up	New
CNOT4(4850)_Up,PDXDC1(23042)_Down	New
PCIF1(63935)_Up,UBR1(197131)_Up	New
ELMO1(9844)_Up,PCIF1(63935)_Up	New
AJAP1(55966)_Up,PCIF1(63935)_Up	New

CNOT4(4850)_Up,PCIF1(63935)_Up	New
UBR1(197131)_Up,TARP(445347)_Down	New
ELMO1(9844)_Up,TARP(445347)_Down	New
AJAP1(55966)_Up,TARP(445347)_Down	New
CNOT4(4850)_Up,TARP(445347)_Down	New

Supplementary Table 20. Disassembled relations derived from Top-50 impactful itemsets of Wild type dataset in comparison 1 with a constant degree

Disassembled Relations	References
PTK2B(2185)_Up,STRAP(11171)_Up	New
HSPA1A/HSPA1B(3303)_Up,STRAP(11171)_Up	New
HSPA1A/HSPA1B(3303)_Up,HSPA1A/HSPA1B(3303)_Up	New
HSPA1A/HSPA1B(3303)_Up,MYC(4609)_Up	New
MYC(4609)_Up,STRAP(11171)_Up	New
PTK2B(2185)_Up,HSPA1A/HSPA1B(3303)_Up	New
PTK2B(2185)_Up,MYC(4609)_Up	New
PTK2B(2185)_Up,SPI1(6688)_Down	New
SPI1(6688)_Down,STRAP(11171)_Up	New

Supplementary Table 21. Disassembled relations derived from Top-50 impactful itemsets of Wild type dataset in comparison 1 with various degrees

Disassembled Relations	References
CREBBP(1387)_Down,TNF(7124)_Down	[1]
MYC(4609)_Up,STAT3(6774)_Up	[2]
CREBBP(1387)_Down,TP53(7157)_Down	[3]
MYC(4609)_Up,TP53(7157)_Up	[4]
CREBBP(1387)_Down,HNF4A(3172)_Down	[5]
CREBBP(1387)_Down,TP53(7157)_Up	[3]*
MYC(4609)_Up,TP53(7157)_Down	[4]*
CREBBP(1387)_Down,MYC(4609)_Up	[6]*
CREBBP(1387)_Down,SPI1(6688)_Down	New
MYC(4609)_Up,SPI1(6688)_Down	New
CREBBP(1387)_Down,STRAP(11171)_Up	New
MYC(4609)_Up,STRAP(11171)_Up	New
GLI1(2735)_Down,MYC(4609)_Up	New
CREBBP(1387)_Down,GLI1(2735)_Down	New
PDX1(3651)_Down,MYC(4609)_Up	New
CREBBP(1387)_Down,PDX1(3651)_Down	New
GCK(2645)_Down,MYC(4609)_Up	New
CREBBP(1387)_Down,GCK(2645)_Down	New
MYC(4609)_Up,NEUROD1(4760)_Down	New
CREBBP(1387)_Down,NEUROD1(4760)_Down	New
MYC(4609)_Up,SP4(6671)_Down	New
CREBBP(1387)_Down,SP4(6671)_Down	New
PTK2B(2185)_Up,MYC(4609)_Up	New
CREBBP(1387)_Down,PTK2B(2185)_Up	New
HSPA1A/HSPA1B(3303)_Up,MYC(4609)_Up	New
CREBBP(1387)_Down,HSPA1A/HSPA1B(3303)_Up	New
EGR1(1958)_Up,MYC(4609)_Up	New
CREBBP(1387)_Down,EGR1(1958)_Up	New
MYC(4609)_Up,MAPK1(5594)_Down	New
CREBBP(1387)_Down,MAPK1(5594)_Down	New
CREBBP(1387)_Down,STAT3(6774)_Up	New
MYC(4609)_Up,SPRY3(10251)_Down	New
CREBBP(1387)_Down,SPRY3(10251)_Down	New
MYC(4609)_Up,SCNN1A(6337)_Down	New
CREBBP(1387)_Down,SCNN1A(6337)_Down	New
HNF4A(3172)_Down,MYC(4609)_Up	New
MYC(4609)_Up,TNF(7124)_Down	New
MYC(4609)_Up,NCOA2(10499)_Down	New
CREBBP(1387)_Down,NCOA2(10499)_Down	New

IFNG(3458)_Down,MYC(4609)_Up	New
CREBBP(1387)_Down,IFNG(3458)_Down	New
CSF2RA(1438)_Down,MYC(4609)_Up	New
CREBBP(1387)_Down,CSF2RA(1438)_Down	New
IL8(3576)_Down,MYC(4609)_Up	New
CREBBP(1387)_Down,IL8(3576)_Down	New
PDX1(3651)_Up,MYC(4609)_Up	New
CREBBP(1387)_Down,PDX1(3651)_Up	New
IFNB1(3456)_Down,MYC(4609)_Up	New
CREBBP(1387)_Down,IFNB1(3456)_Down	New
MYC(4609)_Up,CXCL11(6373)_Down	New
CREBBP(1387)_Down,CXCL11(6373)_Down	New
MYC(4609)_Up,SOCS4(122809)_Down	New
CREBBP(1387)_Down,SOCS4(122809)_Down	New
MYC(4609)_Up,SHISA5(51246)_Up	New
CREBBP(1387)_Down,SHISA5(51246)_Up	New
LTBR(4055)_Down,MYC(4609)_Up	New
CREBBP(1387)_Down,LTBR(4055)_Down	New
MYC(4609)_Up,PEX11A(8800)_Down	New
CREBBP(1387)_Down,PEX11A(8800)_Down	New
BAI1(575)_Up,MYC(4609)_Up	New
BAI1(575)_Up,CREBBP(1387)_Down	New

*: Inconsistent with literature.

Supplementary Table 22. Disassembled relations derived from Top-50 impactful itemsets of F72A/R73A dataset in comparison 1 of undifferentiation control method

Disassembled Relations	References
ITPK1(3705)_Down,RUSC2(9853)_Up	New
RUSC2(9853)_Up,PPIF(10105)_Up	New
ITPK1(3705)_Down,PPIF(10105)_Up	New
RUSC2(9853)_Up,MINA(84864)_Down	New
ITPK1(3705)_Down,MINA(84864)_Down	New
RUSC2(9853)_Up,LMCD1(29995)_Up	New
ITPK1(3705)_Down,LMCD1(29995)_Up	New
KSR1(8844)_Down,RUSC2(9853)_Up	New
ITPK1(3705)_Down,KSR1(8844)_Down	New
RUSC2(9853)_Up,RMND5B(64777)_Up	New
ITPK1(3705)_Down,RMND5B(64777)_Up	New
RUSC2(9853)_Up,RBMXL2(27288)_Down	New
ITPK1(3705)_Down,RBMXL2(27288)_Down	New
PLXNB1(5364)_Up,RUSC2(9853)_Up	New
ITPK1(3705)_Down,PLXNB1(5364)_Up	New
RUSC2(9853)_Up,ELOVL1(64834)_Down	New
ITPK1(3705)_Down,ELOVL1(64834)_Down	New
DSG1(1828)_Down,RUSC2(9853)_Up	New
DSG1(1828)_Down,ITPK1(3705)_Down	New
RUSC2(9853)_Up,C11orf54(28970)_Up	New
ITPK1(3705)_Down,C11orf54(28970)_Up	New
SH3PXD2A(9644)_Down,RUSC2(9853)_Up	New
ITPK1(3705)_Down,SH3PXD2A(9644)_Down	New
PML(5371)_Down,RUSC2(9853)_Up	New
ITPK1(3705)_Down,PML(5371)_Down	New
RUSC2(9853)_Up,C3orf18(51161)_Down	New
ITPK1(3705)_Down,C3orf18(51161)_Down	New
ACTB(60)_Down,RUSC2(9853)_Up	New
ACTB(60)_Down,ITPK1(3705)_Down	New
LMCD1(29995)_Up,MINA(84864)_Down	New
SH3PXD2A(9644)_Down,MINA(84864)_Down	New
SH3PXD2A(9644)_Down,LMCD1(29995)_Up	New
KSR1(8844)_Down,MINA(84864)_Down	New
KSR1(8844)_Down,LMCD1(29995)_Up	New
RMND5B(64777)_Up,MINA(84864)_Down	New
LMCD1(29995)_Up,RMND5B(64777)_Up	New
RBMXL2(27288)_Down,MINA(84864)_Down	New
RBMXL2(27288)_Down,LMCD1(29995)_Up	New

DNASE1L3(1776)_Up,RUSC2(9853)_Up	New
DNASE1L3(1776)_Up,MINA(84864)_Down	New
DNASE1L3(1776)_Up,ITPK1(3705)_Down	New
DNASE1L3(1776)_Up,LMCD1(29995)_Up	New
PPIF(10105)_Up,MINA(84864)_Down	New
PPIF(10105)_Up,LMCD1(29995)_Up	New
CRHR1(1394)_Down,RUSC2(9853)_Up	New
CRHR1(1394)_Down,ITPK1(3705)_Down	New
HELLS(3070)_Down,RUSC2(9853)_Up	New
HELLS(3070)_Down,ITPK1(3705)_Down	New
RUSC2(9853)_Up,KCTD17(79734)_Down	New
ITPK1(3705)_Down,KCTD17(79734)_Down	New
RUSC2(9853)_Up,LOC644656(644656)_Down	New
ITPK1(3705)_Down,LOC644656(644656)_Down	New
RUSC2(9853)_Up,KIF20A(10112)_Down	New
ITPK1(3705)_Down,KIF20A(10112)_Down	New
C1QBP(708)_Down,RUSC2(9853)_Up	New
C1QBP(708)_Down,ITPK1(3705)_Down	New
PLCG2(5336)_Down,RUSC2(9853)_Up	New
ITPK1(3705)_Down,PLCG2(5336)_Down	New
RUSC2(9853)_Up,TCEB3B(51224)_Down	New
ITPK1(3705)_Down,TCEB3B(51224)_Down	New
RUSC2(9853)_Up,GPR157(80045)_Down	New
ITPK1(3705)_Down,GPR157(80045)_Down	New
MAGEA1(4100)_Down,RUSC2(9853)_Up	New
MAGEA1(4100)_Down,MINA(84864)_Down	New
ITPK1(3705)_Down,MAGEA1(4100)_Down	New
MAGEA1(4100)_Down,LMCD1(29995)_Up	New
RUSC2(9853)_Up,MFSD5(84975)_Down	New
MINA(84864)_Down,MFSD5(84975)_Down	New
ITPK1(3705)_Down,MFSD5(84975)_Down	New
LMCD1(29995)_Up,MFSD5(84975)_Down	New
PLXNB1(5364)_Up,MINA(84864)_Down	New
PLXNB1(5364)_Up,LMCD1(29995)_Up	New
ELOVL1(64834)_Down,MINA(84864)_Down	New
LMCD1(29995)_Up,ELOVL1(64834)_Down	New

Supplementary Table 23. Disassembled relations derived from Top-50 impactful itemsets of F72A/R73A dataset in comparison 1 with a constant degree

Disassembled Relations	References
TP53(7157)_Down,KAT2B(8850)_Down	[3]
NCOA1(8648)_Down,KAT2B(8850)_Down	[7]
AQP2(359)_Up,CRMP1(1400)_Up	New
AQP2(359)_Up,KAT2B(8850)_Down	New
BIRC2(329)_Up,CRMP1(1400)_Up	New
BIRC2(329)_Up,KAT2B(8850)_Down	New
CCL2(6347)_Down,KAT2B(8850)_Down	New
CCL2(6347)_Down,NCOA1(8648)_Down	New
CCL2(6347)_Down,THBS1(7057)_Down	New
CDKN2B(1030)_Down,CRMP1(1400)_Up	New
CDKN2B(1030)_Down,CTBP1(1487)_Down	New
CDKN2B(1030)_Down,KAT2B(8850)_Down	New
CRMP1(1400)_Up,CTBP1(1487)_Down	New
CRMP1(1400)_Up,EPS15(2060)_Up	New
CRMP1(1400)_Up,FIGF(2277)_Down	New
CRMP1(1400)_Up,GADD45G(10912)_Down	New
CRMP1(1400)_Up,IL6(3569)_Down	New
CRMP1(1400)_Up,JUP(3728)_Down	New
CRMP1(1400)_Up,KAT2B(8850)_Down	New
CRMP1(1400)_Up,MAP2K1(5604)_Down	New
CRMP1(1400)_Up,MAP2K6(5608)_Down	New
CRMP1(1400)_Up,NCOA1(8648)_Down	New
CRMP1(1400)_Up,NCOA2(10499)_Down	New
CRMP1(1400)_Up,NOTCH3(4854)_Down	New
CRMP1(1400)_Up,PABPN1(8106)_Up	New
CRMP1(1400)_Up,RB1(5925)_Up	New
CRMP1(1400)_Up,SERPINE1(5054)_Down	New
CRMP1(1400)_Up,SESN3(143686)_Down	New
CRMP1(1400)_Up,SIAH1(6477)_Up	New
CRMP1(1400)_Up,TEC(7006)_Up	New
CRMP1(1400)_Up,THBS1(7057)_Down	New
CRMP1(1400)_Up,U2AF1(7307)_Up	New
CTBP1(1487)_Down,CCL2(6347)_Down	New
CTBP1(1487)_Down,EPS15(2060)_Up	New
CTBP1(1487)_Down,FIGF(2277)_Down	New
CTBP1(1487)_Down,JUP(3728)_Down	New
CTBP1(1487)_Down,KAT2B(8850)_Down	New
CTBP1(1487)_Down,NCOA1(8648)_Down	New
CTBP1(1487)_Down,SESN3(143686)_Down	New

CTBP1(1487)_Down,SIAH1(6477)_Up	New
CTBP1(1487)_Down,SPRY3(10251)_Up	New
CTBP1(1487)_Down,TEC(7006)_Up	New
CTBP1(1487)_Down,TP53(7157)_Down	New
CTBP1(1487)_Down,U2AF1(7307)_Up	New
EPS15(2060)_Up,CCL2(6347)_Down	New
EPS15(2060)_Up,FIGF(2277)_Down	New
EPS15(2060)_Up,KAT2B(8850)_Down	New
FIGF(2277)_Down,CCL2(6347)_Down	New
FIGF(2277)_Down,KAT2B(8850)_Down	New
IL6(3569)_Down,KAT2B(8850)_Down	New
JUP(3728)_Down,KAT2B(8850)_Down	New
KAT2B(8850)_Down,GADD45G(10912)_Down	New
KAT2B(8850)_Down,NCOA2(10499)_Down	New
KAT2B(8850)_Down,SESN3(143686)_Down	New
KAT2B(8850)_Down,SPRY3(10251)_Up	New
KLK3(354)_Down,CRMP1(1400)_Up	New
KLK3(354)_Down,KAT2B(8850)_Down	New
MAP2K1(5604)_Down,KAT2B(8850)_Down	New
MAP2K6(5608)_Down,KAT2B(8850)_Down	New
NOTCH3(4854)_Down,CCL2(6347)_Down	New
NOTCH3(4854)_Down,KAT2B(8850)_Down	New
PABPN1(8106)_Up,KAT2B(8850)_Down	New
RB1(5925)_Up,KAT2B(8850)_Down	New
SERPINE1(5054)_Down,KAT2B(8850)_Down	New
SIAH1(6477)_Up,KAT2B(8850)_Down	New
TEC(7006)_Up,KAT2B(8850)_Down	New
THBS1(7057)_Down,KAT2B(8850)_Down	New
U2AF1(7307)_Up,KAT2B(8850)_Down	New

Supplementary Table 24. Disassembled relations derived from Top-50 impactful itemsets of F72A/R73A dataset in comparison 1 with various degrees

Disassembled Relations	References
IL8(3576)_Down,TP53(7157)_Down	[8]
MAP2K1(5604)_Down,TP53(7157)_Down	[9]
NCOA1(8648)_Down,KAT2B(8850)_Down	[7]
TP53(7157)_Down,TP73(7161)_Down	[10]
TP73(7161)_Down,KAT2B(8850)_Down	[11]
MAP2K6(5608)_Down,TP53(7157)_Down	[9]
NOS2(4843)_Down,TP53(7157)_Down	[12]
MAPK8(5599)_Down,TP53(7157)_Down	[13]
NOTCH3(4854)_Down,TP53(7157)_Down	[14]
TP53(7157)_Down,KAT2B(8850)_Down	[3]
MAPK1(5594)_Down,TP53(7157)_Down	[15]
MYC(4609)_Down,TP53(7157)_Down	[4]
ESR1(2099)_Down,TP53(7157)_Down	[16]
BAX(581)_Up,TP53(7157)_Down	[17]*
TNF(7124)_Up,KAT2B(8850)_Down	[18]*
NOS2(4843)_Up,TP53(7157)_Down	[12]*
TP53(7157)_Up,KAT2B(8850)_Down	[3]*
RB1(5925)_Up,TP53(7157)_Down	[19]*
ARNTL(406)_Down,KAT2B(8850)_Down	New
ARNTL(406)_Down,TP53(7157)_Down	New
BAX(581)_Up,KAT2B(8850)_Down	New
CCND1(595)_Down,CTBP1(1487)_Down	New
CCND1(595)_Down,KAT2B(8850)_Down	New
CCND1(595)_Down,TP53(7157)_Down	New
CDKN2B(1030)_Down,KAT2B(8850)_Down	New
CDKN2B(1030)_Down,TP53(7157)_Down	New
CIITA(4261)_Down,KAT2B(8850)_Down	New
CIITA(4261)_Down,TP53(7157)_Down	New
CRMP1(1400)_Up,KAT2B(8850)_Down	New
CRMP1(1400)_Up,NCOA1(8648)_Down	New
CRMP1(1400)_Up,TP53(7157)_Down	New
CTBP1(1487)_Down,KAT2B(8850)_Down	New
CTBP1(1487)_Down,TP53(7157)_Down	New
EPS15(2060)_Up,KAT2B(8850)_Down	New
EPS15(2060)_Up,TP53(7157)_Down	New
ESR1(2099)_Down,KAT2B(8850)_Down	New
FIGF(2277)_Down,KAT2B(8850)_Down	New
FIGF(2277)_Down,TP53(7157)_Down	New
GLI1(2735)_Down,KAT2B(8850)_Down	New

GLI1(2735)_Down,TP53(7157)_Down	New
IAPP(3375)_Down,KAT2B(8850)_Down	New
IAPP(3375)_Down,TP53(7157)_Down	New
IL12A(3592)_Down,KAT2B(8850)_Down	New
IL12A(3592)_Down,TP53(7157)_Down	New
IL8(3576)_Down,KAT2B(8850)_Down	New
IRF7(3665)_Down,KAT2B(8850)_Down	New
IRF7(3665)_Down,TP53(7157)_Down	New
JUP(3728)_Down,TP53(7157)_Down	New
KAT2B(8850)_Down,GADD45G(10912)_Down	New
KAT2B(8850)_Down,NCOA2(10499)_Down	New
KAT2B(8850)_Down,SESN3(143686)_Down	New
MAP2K1(5604)_Down,KAT2B(8850)_Down	New
MAP2K6(5608)_Down,KAT2B(8850)_Down	New
MAPK1(5594)_Down,KAT2B(8850)_Down	New
MAPK8(5599)_Down,KAT2B(8850)_Down	New
MYC(4609)_Down,KAT2B(8850)_Down	New
NOS2(4843)_Down,KAT2B(8850)_Down	New
NOS2(4843)_Up,KAT2B(8850)_Down	New
NOTCH3(4854)_Down,KAT2B(8850)_Down	New
NR5A1(2516)_Up,KAT2B(8850)_Down	New
NR5A1(2516)_Up,TP53(7157)_Down	New
PABPN1(8106)_Up,KAT2B(8850)_Down	New
PAX6(5080)_Up,KAT2B(8850)_Down	New
PAX6(5080)_Up,TP53(7157)_Down	New
PRKRA(8575)_Down,KAT2B(8850)_Down	New
RB1(5925)_Up,KAT2B(8850)_Down	New
SMAD3(4088)_Down,KAT2B(8850)_Down	New
SMAD3(4088)_Down,TP53(7157)_Down	New
SP3(6670)_Down,KAT2B(8850)_Down	New
SP3(6670)_Down,TP53(7157)_Down	New
STAT3(6774)_Down,KAT2B(8850)_Down	New
STAT3(6774)_Down,TP53(7157)_Down	New
TEC(7006)_Up,KAT2B(8850)_Down	New
TEC(7006)_Up,TP53(7157)_Down	New
THBS1(7057)_Down,KAT2B(8850)_Down	New
THBS1(7057)_Down,TP53(7157)_Down	New
TNF(7124)_Up,TP53(7157)_Down	New
TP53(7157)_Down,GADD45G(10912)_Down	New
TP53(7157)_Down,NCOA1(8648)_Down	New
TP53(7157)_Down,NCOA2(10499)_Down	New

TP53(7157)_Down,PABPN1(8106)_Up	New
TP53(7157)_Down,PRKRA(8575)_Down	New
TP53(7157)_Down,SESN3(143686)_Down	New
TP53(7157)_Down,TP53(7157)_Up	New
TP53(7157)_Down,U2AF1(7307)_Up	New
TP53(7157)_Down,ZBTB16(7704)_Down	New
U2AF1(7307)_Up,KAT2B(8850)_Down	New
ZBTB16(7704)_Down,KAT2B(8850)_Down	New

*: Inconsistent with literature.

Supplementary Table 25. Disassembled relations derived from Top-50 impactful itemsets of Wild type dataset in comparison 2 of undifferentiation control method

Disassembled Relations	References
ELMO1(9844)_Up,UBR1(197131)_Up	New
AJAP1(55966)_Up,UBR1(197131)_Up	New
ELMO1(9844)_Up,AJAP1(55966)_Up	New
CNOT4(4850)_Up,UBR1(197131)_Up	New
CNOT4(4850)_Up,ELMO1(9844)_Up	New
CNOT4(4850)_Up,AJAP1(55966)_Up	New
TMSB4Y(9087)_Down,UBR1(197131)_Up	New
TMSB4Y(9087)_Down,ELMO1(9844)_Up	New
TMSB4Y(9087)_Down,AJAP1(55966)_Up	New
CNOT4(4850)_Up,TMSB4Y(9087)_Down	New
APIP(51074)_Down,UBR1(197131)_Up	New
ELMO1(9844)_Up,APIP(51074)_Down	New
APIP(51074)_Down,AJAP1(55966)_Up	New
CNOT4(4850)_Up,APIP(51074)_Down	New
TMSB4Y(9087)_Down,APIP(51074)_Down	New
UBR1(197131)_Up,CCDC111(201973)_Up	New
ELMO1(9844)_Up,CCDC111(201973)_Up	New
AJAP1(55966)_Up,CCDC111(201973)_Up	New
CNOT4(4850)_Up,CCDC111(201973)_Up	New
HNF4G(3174)_Down,UBR1(197131)_Up	New
HNF4G(3174)_Down,ELMO1(9844)_Up	New
HNF4G(3174)_Down,AJAP1(55966)_Up	New
HNF4G(3174)_Down,CNOT4(4850)_Up	New
WDR52(55779)_Down,UBR1(197131)_Up	New
ELMO1(9844)_Up,WDR52(55779)_Down	New
WDR52(55779)_Down,AJAP1(55966)_Up	New
CNOT4(4850)_Up,WDR52(55779)_Down	New
UBR1(197131)_Up,LOC100288281(100288281)_Down	New
ELMO1(9844)_Up,LOC100288281(100288281)_Down	New
AJAP1(55966)_Up,LOC100288281(100288281)_Down	New
CNOT4(4850)_Up,LOC100288281(100288281)_Down	New
NUP62(23636)_Down,UBR1(197131)_Up	New
ELMO1(9844)_Up,NUP62(23636)_Down	New
NUP62(23636)_Down,AJAP1(55966)_Up	New
CNOT4(4850)_Up,NUP62(23636)_Down	New
PCIF1(63935)_Up,UBR1(197131)_Up	New
ELMO1(9844)_Up,PCIF1(63935)_Up	New
AJAP1(55966)_Up,PCIF1(63935)_Up	New

CNOT4(4850)_Up,PCIF1(63935)_Up	New
PBX3(5090)_Down,UBR1(197131)_Up	New
PBX3(5090)_Down,ELMO1(9844)_Up	New
PBX3(5090)_Down,AJAP1(55966)_Up	New
CNOT4(4850)_Up,PBX3(5090)_Down	New

Supplementary Table 26. Disassembled relations derived from Top-50 impactful itemsets of Wild type dataset in comparison 2 with a constant degree

Disassembled Relations	References
MYC(4609)_Up,STAT3(6774)_Up	[2]
MYC(4609)_Up,PLAU(5328)_Down	[20]*
MYC(4609)_Up,TBP(6908)_Down	[21]*
BCL2(596)_Down,MYC(4609)_Up	[22]*
MYC(4609)_Up,TCF7L2(6934)_Down	[23]*
CREBBP(1387)_Down,MYC(4609)_Up	[6]*
BCL2(596)_Down,MAP2K4(6416)_Up	New
BCL2(596)_Down,STRAP(11171)_Up	New
CCND1(595)_Up,MAP2K4(6416)_Up	New
CCND1(595)_Up,MYC(4609)_Up	New
CCND1(595)_Up,STRAP(11171)_Up	New
CREBBP(1387)_Down,MAP2K4(6416)_Up	New
CREBBP(1387)_Down,STRAP(11171)_Up	New
CXCL11(6373)_Down,MAP2K4(6416)_Up	New
CXCL11(6373)_Down,STRAP(11171)_Up	New
DNAJC3(5611)_Up,STRAP(11171)_Up	New
GCK(2645)_Down,MAP2K4(6416)_Up	New
GCK(2645)_Down,MYC(4609)_Up	New
GCK(2645)_Down,STRAP(11171)_Up	New
GNA12(2768)_Up,MYC(4609)_Up	New
GNA12(2768)_Up,STRAP(11171)_Up	New
HNF4A(3172)_Down,MYC(4609)_Up	New
HNF4A(3172)_Down,STRAP(11171)_Up	New
IL18(3606)_Down,MYC(4609)_Up	New
IL18(3606)_Down,STRAP(11171)_Up	New
IL1A(3552)_Down,MYC(4609)_Up	New
IL1A(3552)_Down,STRAP(11171)_Up	New
MAP2K4(6416)_Up,NRIP1(8204)_Up	New
MAP2K4(6416)_Up,SKI(6497)_Up	New
MAP2K4(6416)_Up,SOCS4(122809)_Down	New
MAP2K4(6416)_Up,STRAP(11171)_Up	New
MAP2K4(6416)_Up,TBP(6908)_Down	New
MAP2K4(6416)_Up,TCF7L2(6934)_Down	New
MAP2K4(6416)_Up,TYR(7299)_Down	New
MAPK1(5594)_Down,STRAP(11171)_Up	New
MYC(4609)_Up,CXCL11(6373)_Down	New
MYC(4609)_Up,DNAJC3(5611)_Up	New
MYC(4609)_Up,FIS1(51024)_Up	New
MYC(4609)_Up,MAP2K4(6416)_Up	New

MYC(4609)_Up,MAPK1(5594)_Down	New
MYC(4609)_Up,NEUROD1(4760)_Down	New
MYC(4609)_Up,NRIP1(8204)_Up	New
MYC(4609)_Up,POU2AF1(5450)_Down	New
MYC(4609)_Up,PPP1R15A(23645)_Up	New
MYC(4609)_Up,RAC1(5879)_Up	New
MYC(4609)_Up,RAN(5901)_Up	New
MYC(4609)_Up,RB1CC1(9821)_Up	New
MYC(4609)_Up,SCNN1A(6337)_Down	New
MYC(4609)_Up,SKI(6497)_Up	New
MYC(4609)_Up,SNW1(22938)_Up	New
MYC(4609)_Up,SOCS4(122809)_Down	New
MYC(4609)_Up,SPRY3(10251)_Down	New
MYC(4609)_Up,STRAP(11171)_Up	New
MYC(4609)_Up,TYR(7299)_Down	New
NEUROD1(4760)_Down,STRAP(11171)_Up	New
NRIP1(8204)_Up,STRAP(11171)_Up	New
PLAU(5328)_Down,STRAP(11171)_Up	New
POU2AF1(5450)_Down,MAP2K4(6416)_Up	New
POU2AF1(5450)_Down,STRAP(11171)_Up	New
RAC1(5879)_Up,STRAP(11171)_Up	New
RAN(5901)_Up,STRAP(11171)_Up	New
RB1CC1(9821)_Up,STRAP(11171)_Up	New
RHOA(387)_Up,MAP2K4(6416)_Up	New
RHOA(387)_Up,MYC(4609)_Up	New
RHOA(387)_Up,STRAP(11171)_Up	New
SCNN1A(6337)_Down,STRAP(11171)_Up	New
SKI(6497)_Up,STRAP(11171)_Up	New
SPRY3(10251)_Down,STRAP(11171)_Up	New
STAT3(6774)_Up,STRAP(11171)_Up	New
STRAP(11171)_Up,FIS1(51024)_Up	New
STRAP(11171)_Up,PPP1R15A(23645)_Up	New
STRAP(11171)_Up,SNW1(22938)_Up	New
STRAP(11171)_Up,SOCS4(122809)_Down	New
TBP(6908)_Down,STRAP(11171)_Up	New
TCF7L2(6934)_Down,STRAP(11171)_Up	New
TYR(7299)_Down,STRAP(11171)_Up	New

*: Inconsistent with literature.

Supplementary Table 27. Disassembled relations derived from Top-50 impactful itemsets of Wild type dataset in comparison 2 with various degrees

Disassembled Relations	References
MYC(4609)_Up,STAT3(6774)_Up	[2]
CCND1(595)_Up,STAT3(6774)_Up	[24]
MAP2K4(6416)_Up,STAT3(6774)_Up	[25]
MYC(4609)_Up,PLAU(5328)_Down	[20]*
TP53(7157)_Down,STRAP(11171)_Up	[26]*
MYC(4609)_Up,TP53(7157)_Down	[4]*
MYC(4609)_Up,TCF7L2(6934)_Down	[23]*
CREBBP(1387)_Down,MYC(4609)_Up	[6]*
CCND1(595)_Up,CREBBP(1387)_Down	New
CCND1(595)_Up,FIGF(2277)_Down	New
CCND1(595)_Up,MAP2K4(6416)_Up	New
CCND1(595)_Up,MYC(4609)_Up	New
CCND1(595)_Up,POU2AF1(5450)_Down	New
CCND1(595)_Up,STRAP(11171)_Up	New
CCND1(595)_Up,TP53(7157)_Down	New
CREBBP(1387)_Down,MAP2K4(6416)_Up	New
CREBBP(1387)_Down,POU2AF1(5450)_Down	New
CREBBP(1387)_Down,STRAP(11171)_Up	New
CXCL11(6373)_Down,STRAP(11171)_Up	New
CXCL11(6373)_Up,STRAP(11171)_Up	New
FIGF(2277)_Down,MYC(4609)_Up	New
FIGF(2277)_Down,STRAP(11171)_Up	New
GCK(2645)_Down,MYC(4609)_Up	New
GCK(2645)_Down,STRAP(11171)_Up	New
IFNB1(3456)_Down,MYC(4609)_Up	New
IFNB1(3456)_Down,STRAP(11171)_Up	New
IL18(3606)_Down,MYC(4609)_Up	New
IL18(3606)_Down,STRAP(11171)_Up	New
IL1A(3552)_Down,MYC(4609)_Up	New
IL1A(3552)_Down,STRAP(11171)_Up	New
IL8(3576)_Down,MYC(4609)_Up	New
IL8(3576)_Down,STRAP(11171)_Up	New
MAP2K4(6416)_Up,STRAP(11171)_Up	New
MAPK1(5594)_Down,STRAP(11171)_Up	New
MYC(4609)_Up,CXCL11(6373)_Down	New
MYC(4609)_Up,CXCL11(6373)_Up	New
MYC(4609)_Up,FIS1(51024)_Up	New
MYC(4609)_Up,MAP2K4(6416)_Up	New
MYC(4609)_Up,MAPK1(5594)_Down	New

MYC(4609)_Up,NCOA1(8648)_Down	New
MYC(4609)_Up,NCOA2(10499)_Down	New
MYC(4609)_Up,NEUROD1(4760)_Down	New
MYC(4609)_Up,NRIP1(8204)_Up	New
MYC(4609)_Up,POU2AF1(5450)_Down	New
MYC(4609)_Up,RAN(5901)_Up	New
MYC(4609)_Up,RB1CC1(9821)_Up	New
MYC(4609)_Up,SKI(6497)_Up	New
MYC(4609)_Up,SNW1(22938)_Up	New
MYC(4609)_Up,SOCS4(122809)_Down	New
MYC(4609)_Up,STRAP(11171)_Up	New
MYC(4609)_Up,TNF(7124)_Down	New
MYC(4609)_Up,TRAF2(7186)_Up	New
MYC(4609)_Up,TYR(7299)_Down	New
NCOA1(8648)_Down,STRAP(11171)_Up	New
NCOA2(10499)_Down,STRAP(11171)_Up	New
NEUROD1(4760)_Down,STRAP(11171)_Up	New
NRIP1(8204)_Up,STRAP(11171)_Up	New
PDX1(3651)_Down,MYC(4609)_Up	New
PDX1(3651)_Down,STRAP(11171)_Up	New
PLAU(5328)_Down,STRAP(11171)_Up	New
POU2AF1(5450)_Down,STRAP(11171)_Up	New
RAN(5901)_Up,STRAP(11171)_Up	New
RB1CC1(9821)_Up,STRAP(11171)_Up	New
RHOA(387)_Up,CCND1(595)_Up	New
RHOA(387)_Up,MYC(4609)_Up	New
SKI(6497)_Up,STRAP(11171)_Up	New
STAT3(6774)_Up,STRAP(11171)_Up	New
STRAP(11171)_Up,FIS1(51024)_Up	New
STRAP(11171)_Up,SNW1(22938)_Up	New
STRAP(11171)_Up,SOCS4(122809)_Down	New
TCF7L2(6934)_Down,STRAP(11171)_Up	New
TNF(7124)_Down,STRAP(11171)_Up	New
TRAF2(7186)_Up,STRAP(11171)_Up	New
TYR(7299)_Down,STRAP(11171)_Up	New

*: Inconsistent with literature.

Supplementary Table 28. Disassembled relations derived from Top-50 impactful itemsets of R80A dataset in comparison 2 of undifferentiation control method

Disassembled Relations	References
RUSC2(9853)_Up,MRPL39(54148)_Up	New
RUSC2(9853)_Up,TMCO7(79613)_Down	New
TCOF1(6949)_Up,RUSC2(9853)_Up	New
MRPL39(54148)_Up,TMCO7(79613)_Down	New
MRPL39(54148)_Up,DENND5B(160518)_Up	New
RUSC2(9853)_Up,DENND5B(160518)_Up	New
TMCO7(79613)_Down,DENND5B(160518)_Up	New
TCOF1(6949)_Up,DENND5B(160518)_Up	New
TCOF1(6949)_Up,TMCO7(79613)_Down	New
TCOF1(6949)_Up,MRPL39(54148)_Up	New
SASH1(23328)_Up,DENND5B(160518)_Up	New
RUSC2(9853)_Up,SASH1(23328)_Up	New
RUSC2(9853)_Up,OR2H1(26716)_Up	New
OR2H1(26716)_Up,TMCO7(79613)_Down	New
SASH1(23328)_Up,MRPL39(54148)_Up	New
SASH1(23328)_Up,TMCO7(79613)_Down	New
OR2H1(26716)_Up,MRPL39(54148)_Up	New
SLC6A12(6539)_Up,RUSC2(9853)_Up	New
SLC6A12(6539)_Up,TMCO7(79613)_Down	New
PDS5B(23047)_Up,DENND5B(160518)_Up	New
RUSC2(9853)_Up,PDS5B(23047)_Up	New
SEMA3G(56920)_Up,DENND5B(160518)_Up	New
RUSC2(9853)_Up,SEMA3G(56920)_Up	New
SLC6A12(6539)_Up,MRPL39(54148)_Up	New
MRPL39(54148)_Up,SEMA3G(56920)_Up	New
TCOF1(6949)_Up,SASH1(23328)_Up	New
PDS5B(23047)_Up,MRPL39(54148)_Up	New
PDS5B(23047)_Up,TMCO7(79613)_Down	New
SEMA3G(56920)_Up,TMCO7(79613)_Down	New
TCOF1(6949)_Up,OR2H1(26716)_Up	New
RUSC2(9853)_Up,AQPEP(206338)_Up	New
OR2H1(26716)_Up,DENND5B(160518)_Up	New

Supplementary Table 29. Disassembled relations derived from Top-50 impactful itemsets of R80A dataset in comparison 2 with a constant degree

Disassembled Relations	References
CEBPB(1051)_Up,DPYSL2(1808)_Up	New
CEBPB(1051)_Up,MSX1(4487)_Down	New
CIITA(4261)_Down,MSX1(4487)_Down	New
DPYSL2(1808)_Up,ARHGEF12(23365)_Down	New
DPYSL2(1808)_Up,CIITA(4261)_Down	New
DPYSL2(1808)_Up,ELK1(2002)_Up	New
DPYSL2(1808)_Up,ETS1(2113)_Down	New
DPYSL2(1808)_Up,FIGF(2277)_Down	New
DPYSL2(1808)_Up,GNA13(10672)_Down	New
DPYSL2(1808)_Up,IAPP(3375)_Down	New
DPYSL2(1808)_Up,IL12A(3592)_Down	New
DPYSL2(1808)_Up,IL8(3576)_Down	New
DPYSL2(1808)_Up,KAT2B(8850)_Down	New
DPYSL2(1808)_Up,KHDRBS1(10657)_Up	New
DPYSL2(1808)_Up,LEF1(51176)_Up	New
DPYSL2(1808)_Up,MSX1(4487)_Down	New
DPYSL2(1808)_Up,MYC(4609)_Down	New
DPYSL2(1808)_Up,NCOA1(8648)_Down	New
DPYSL2(1808)_Up,PABPN1(8106)_Up	New
DPYSL2(1808)_Up,PDX1(3651)_Down	New
DPYSL2(1808)_Up,POLA1(5422)_Up	New
DPYSL2(1808)_Up,RAC1(5879)_Up	New
DPYSL2(1808)_Up,RASA1(5921)_Down	New
DPYSL2(1808)_Up,RB1(5925)_Up	New
DPYSL2(1808)_Up,RBMS1(5937)_Up	New
DPYSL2(1808)_Up,SIAH1(6477)_Up	New
DPYSL2(1808)_Up,STAT1(6772)_Up	New
DPYSL2(1808)_Up,TEC(7006)_Up	New
DPYSL2(1808)_Up,TP53(7157)_Down	New
DPYSL2(1808)_Up,VDR(7421)_Down	New
ELK1(2002)_Up,MSX1(4487)_Down	New
ETS1(2113)_Down,MSX1(4487)_Down	New
FIGF(2277)_Down,MSX1(4487)_Down	New
FIGF(2277)_Down,RB1(5925)_Up	New
IAPP(3375)_Down,MSX1(4487)_Down	New
IL12A(3592)_Down,MSX1(4487)_Down	New
IL12A(3592)_Down,RB1(5925)_Up	New
IL8(3576)_Down,MSX1(4487)_Down	New
MSX1(4487)_Down,ARHGEF12(23365)_Down	New

MSX1(4487)_Down,GNA13(10672)_Down	New
MSX1(4487)_Down,KAT2B(8850)_Down	New
MSX1(4487)_Down,KHDRBS1(10657)_Up	New
MSX1(4487)_Down,LEF1(51176)_Up	New
MSX1(4487)_Down,MYC(4609)_Down	New
MSX1(4487)_Down,NCOA1(8648)_Down	New
MSX1(4487)_Down,PABPN1(8106)_Up	New
MSX1(4487)_Down,POLA1(5422)_Up	New
MSX1(4487)_Down,RAC1(5879)_Up	New
MSX1(4487)_Down,RASA1(5921)_Down	New
MSX1(4487)_Down,RB1(5925)_Up	New
MSX1(4487)_Down,RBMS1(5937)_Up	New
MSX1(4487)_Down,SIAH1(6477)_Up	New
MSX1(4487)_Down,STAT1(6772)_Up	New
MSX1(4487)_Down,TEC(7006)_Up	New
MSX1(4487)_Down,TP53(7157)_Down	New
MSX1(4487)_Down,VDR(7421)_Down	New
PDX1(3651)_Down,MSX1(4487)_Down	New
RB1(5925)_Up,KAT2B(8850)_Down	New
RB1(5925)_Up,PABPN1(8106)_Up	New
RB1(5925)_Up,RBMS1(5937)_Up	New
RB1(5925)_Up,STAT1(6772)_Up	New
RB1(5925)_Up,TEC(7006)_Up	New
RBMS1(5937)_Up,KAT2B(8850)_Down	New
RBMS1(5937)_Up,PABPN1(8106)_Up	New
RBMS1(5937)_Up,STAT1(6772)_Up	New

Supplementary Table 30. Disassembled relations derived from Top-50 impactful itemsets of R80A dataset in comparison 2 with various degrees

Disassembled Relations	References
IL8(3576)_Down,TP53(7157)_Down	[8]
CEBPA(1050)_Down,TP53(7157)_Down	[27]
TP53(7157)_Down,SMURF1(57154)_Down	[28]
TP53(7157)_Down,GNA13(10672)_Down	[29]
TP53(7157)_Down,KAT2B(8850)_Down	[3]
MYC(4609)_Down,TP53(7157)_Down	[4]
IL6(3569)_Down,TP53(7157)_Down	[8]
ETS1(2113)_Down,TP53(7157)_Down	[30]
IL6(3569)_Down,RB1(5925)_Up	[31]*
RB1(5925)_Up,TP53(7157)_Down	[19]*
NFKB1(4790)_Up,TP53(7157)_Down	[32]*
MYC(4609)_Down,RB1(5925)_Up	[33]*
CEBPA(1050)_Down,DPYSL2(1808)_Up	New
CEBPA(1050)_Down,FIGF(2277)_Down	New
CEBPA(1050)_Down,MSX1(4487)_Down	New
CEBPA(1050)_Down,RB1(5925)_Up	New
DPYSL2(1808)_Up,ETS1(2113)_Down	New
DPYSL2(1808)_Up,FIGF(2277)_Down	New
DPYSL2(1808)_Up,IL12A(3592)_Down	New
DPYSL2(1808)_Up,IL6(3569)_Down	New
DPYSL2(1808)_Up,IL8(3576)_Down	New
DPYSL2(1808)_Up,KAT2B(8850)_Down	New
DPYSL2(1808)_Up,LEF1(51176)_Up	New
DPYSL2(1808)_Up,MSX1(4487)_Down	New
DPYSL2(1808)_Up,MYC(4609)_Down	New
DPYSL2(1808)_Up,NCOA1(8648)_Down	New
DPYSL2(1808)_Up,NFKB1(4790)_Up	New
DPYSL2(1808)_Up,PDX1(3651)_Down	New
DPYSL2(1808)_Up,RASA1(5921)_Down	New
DPYSL2(1808)_Up,RB1(5925)_Up	New
DPYSL2(1808)_Up,SMAD3(4088)_Down	New
DPYSL2(1808)_Up,SMURF1(57154)_Down	New
DPYSL2(1808)_Up,STAT1(6772)_Up	New
DPYSL2(1808)_Up,STAT3(6774)_Up	New
DPYSL2(1808)_Up,TEC(7006)_Up	New
DPYSL2(1808)_Up,TP53(7157)_Down	New
FIGF(2277)_Down,MSX1(4487)_Down	New
FIGF(2277)_Down,RB1(5925)_Up	New
FIGF(2277)_Down,TP53(7157)_Down	New

IL12A(3592)_Down,MSX1(4487)_Down	New
IL12A(3592)_Down,RB1(5925)_Up	New
IL12A(3592)_Down,TP53(7157)_Down	New
IL6(3569)_Down,MSX1(4487)_Down	New
IL8(3576)_Down,RB1(5925)_Up	New
MSX1(4487)_Down,LEF1(51176)_Up	New
MSX1(4487)_Down,MYC(4609)_Down	New
MSX1(4487)_Down,SMURF1(57154)_Down	New
MSX1(4487)_Down,STAT1(6772)_Up	New
MSX1(4487)_Down,TP53(7157)_Down	New
NFKB1(4790)_Up,RB1(5925)_Up	New
PDX1(3651)_Down,TP53(7157)_Down	New
RASA1(5921)_Down,TP53(7157)_Down	New
RB1(5925)_Up,KAT2B(8850)_Down	New
RB1(5925)_Up,LEF1(51176)_Up	New
RB1(5925)_Up,NCOA1(8648)_Down	New
RB1(5925)_Up,SMURF1(57154)_Down	New
RB1(5925)_Up,STAT1(6772)_Up	New
RBMS1(5937)_Up,TP53(7157)_Down	New
SMAD3(4088)_Down,RB1(5925)_Up	New
SMAD3(4088)_Down,TP53(7157)_Down	New
STAT1(6772)_Up,TP53(7157)_Down	New
STAT3(6774)_Up,TP53(7157)_Down	New
TEC(7006)_Up,TP53(7157)_Down	New
TP53(7157)_Down,LEF1(51176)_Up	New
TP53(7157)_Down,NCOA1(8648)_Down	New

*: Inconsistent with literature.

Supplementary Table 31. Disassembled relations derived from Top-50 impactful itemsets of Air dataset in comparison 3 of undifferentiation control method

Disassembled Relations	References
Mela(17276)_Down,Mela(17276)_Down	New
Mela(17276)_Down,FAM167B(230766)_Down	New
Mela(17276)_Down,BC018473(193217)_Down	New
MYCL1(16918)_Down,Mela(17276)_Down	New
Mela(17276)_Down,Bola2(66162)_Down	New
BC018473(193217)_Down,FAM167B(230766)_Down	New
Bola2(66162)_Down,FAM167B(230766)_Down	New
MYCL1(16918)_Down,FAM167B(230766)_Down	New
Mela(17276)_Down,DHCR24(74754)_Down	New
DHCR24(74754)_Down,FAM167B(230766)_Down	New
Mela(17276)_Down,AAR2(68295)_Down	New
AAR2(68295)_Down,FAM167B(230766)_Down	New
Mela(17276)_Down,LOC433053(433053)_Down	New
FAM167B(230766)_Down,LOC433053(433053)_Down	New
CNTN1(12805)_Up,Mela(17276)_Down	New
CNTN1(12805)_Up,FAM167B(230766)_Down	New
DBP(13170)_Down,Mela(17276)_Down	New
DBP(13170)_Down,FAM167B(230766)_Down	New
Mela(17276)_Down,SLC25A13(50799)_Down	New
SLC25A13(50799)_Down,FAM167B(230766)_Down	New
Mela(17276)_Down,OSR2(107587)_Down	New
OSR2(107587)_Down,FAM167B(230766)_Down	New
Mela(17276)_Down,ERLIN1(226144)_Down	New
ERLIN1(226144)_Down,FAM167B(230766)_Down	New

Supplementary Table 32. Disassembled relations derived from Top-50 impactful itemsets of Air dataset in comparison 3 with a constant degree

Disassembled Relations	References
EGR1(13653)_Down,TNF(21926)_Down	[34]
CREB1(12912)_Up,EGR1(13653)_Down	[35]*
BCL2L1(12048)_Up,CXCL10(15945)_Down	New
BCL2L1(12048)_Up,EGR1(13653)_Down	New
CREB1(12912)_Up,CXCL10(15945)_Down	New
CREB1(12912)_Up,CXCL11(56066)_Up	New
CREB1(12912)_Up,EPAS1(13819)_Up	New
CREB1(12912)_Up,HNF4G(30942)_Down	New
CREB1(12912)_Up,PER2(18627)_Down	New
CREB1(12912)_Up,SLC2A2(20526)_Down	New
CREB1(12912)_Up,SOS1(20662)_Down	New
CREB1(12912)_Up,VDR(22337)_Down	New
CXCL10(15945)_Down,BAI1(107831)_Down	New
CXCL10(15945)_Down,CSNK1E(27373)_Up	New
CXCL10(15945)_Down,CXCL11(56066)_Up	New
CXCL10(15945)_Down,FOXO1(56458)_Up	New
CXCL10(15945)_Down,GCK(103988)_Up	New
CXCL10(15945)_Down,IFNA1/IFNA13(15962)_Down	New
CXCL10(15945)_Down,IL1A(16175)_Up	New
CXCL10(15945)_Down,MAPK10(26414)_Down	New
CXCL10(15945)_Down,PPARD(19015)_Up	New
CXCL10(15945)_Down,SLC2A2(20526)_Down	New
CXCL10(15945)_Down,SLC8A1(20541)_Up	New
CXCL10(15945)_Down,SOS1(20662)_Down	New
CXCL10(15945)_Down,STEAP3(68428)_Up	New
CXCL10(15945)_Down,TNF(21926)_Down	New
CXCL10(15945)_Down,TP73(22062)_Down	New
CXCL10(15945)_Down,VDR(22337)_Down	New
CXCL10(15945)_Down,VDR(22337)_Up	New
CXCL10(15945)_Down,WNT1(22408)_Up	New
EGR1(13653)_Down,BAI1(107831)_Down	New
EGR1(13653)_Down,CSNK1E(27373)_Up	New
EGR1(13653)_Down,CXCL10(15945)_Down	New
EGR1(13653)_Down,CXCL11(56066)_Up	New
EGR1(13653)_Down,EPAS1(13819)_Up	New
EGR1(13653)_Down,ESR1(13982)_Up	New
EGR1(13653)_Down,FOXO1(56458)_Up	New
EGR1(13653)_Down,GCK(103988)_Up	New
EGR1(13653)_Down,HNF1B(21410)_Up	New

EGR1(13653)_Down,HNF4G(30942)_Down	New
EGR1(13653)_Down,IFNA1/IFNA13(15962)_Down	New
EGR1(13653)_Down,IFNG(15978)_Up	New
EGR1(13653)_Down,IL1A(16175)_Up	New
EGR1(13653)_Down,MAPK10(26414)_Down	New
EGR1(13653)_Down,MRE11A(17535)_Down	New
EGR1(13653)_Down,PCK1(18534)_Up	New
EGR1(13653)_Down,PER2(18627)_Down	New
EGR1(13653)_Down,PPARD(19015)_Up	New
EGR1(13653)_Down,RRM2(20135)_Down	New
EGR1(13653)_Down,SLC2A2(20526)_Down	New
EGR1(13653)_Down,SLC8A1(20541)_Up	New
EGR1(13653)_Down,SOS1(20662)_Down	New
EGR1(13653)_Down,STEAP3(68428)_Up	New
EGR1(13653)_Down,TP73(22062)_Down	New
EGR1(13653)_Down,TRAF6(22034)_Down	New
EGR1(13653)_Down,VDR(22337)_Down	New
EGR1(13653)_Down,VDR(22337)_Up	New
EGR1(13653)_Down,WNT1(22408)_Up	New
EPAS1(13819)_Up,CXCL10(15945)_Down	New
ESR1(13982)_Up,CXCL10(15945)_Down	New
MRE11A(17535)_Down,VDR(22337)_Down	New
RRM2(20135)_Down,VDR(22337)_Down	New
Runx1(12394)_Down,EGR1(13653)_Down	New
SOCS3(12702)_Down,CXCL10(15945)_Down	New
SOCS3(12702)_Down,EGR1(13653)_Down	New

*: Inconsistent with literature.

Supplementary Table 33. Disassembled relations derived from Top-50 impactful itemsets of Air dataset in comparison 3 with various degrees

Disassembled Relations	References
EGR1(13653)_Down,MYC(17869)_Down	[36]
EGR1(13653)_Down,TNF(21926)_Down	[34]
EGR1(13653)_Down,TP53(22059)_Down	[37]
FOS(14281)_Down,TNF(21926)_Down	[38]
TNF(21926)_Down,TP53(22059)_Down	[39]
APC(11789)_Down,CCND1(12443)_Down	New
APC(11789)_Down,CDH2(12558)_Up	New
APC(11789)_Down,EGR1(13653)_Down	New
APC(11789)_Down,IFNG(15978)_Up	New
APC(11789)_Down,MAPK10(26414)_Down	New
APC(11789)_Down,MYC(17869)_Down	New
APC(11789)_Down,NKX2-2(18088)_Up	New
APC(11789)_Down,PAX6(18508)_Up	New
APC(11789)_Down,PDX1(18609)_Up	New
APC(11789)_Down,PKLR(18770)_Up	New
APC(11789)_Down,SFRP1(20377)_Up	New
APC(11789)_Down,TNF(21926)_Down	New
APC(11789)_Down,VDR(22337)_Down	New
APC(11789)_Down,WNT1(22408)_Up	New
APC(11789)_Up,EGR1(13653)_Down	New
APC(11789)_Up,TNF(21926)_Down	New
CCND1(12443)_Down,EGR1(13653)_Down	New
CCND1(12443)_Down,IL1A(16175)_Up	New
CCND1(12443)_Down,MRE11A(17535)_Down	New
CCND1(12443)_Down,MYC(17869)_Down	New
CCND1(12443)_Down,PAX6(18508)_Up	New
CCND1(12443)_Down,PDX1(18609)_Up	New
CCND1(12443)_Down,TNF(21926)_Down	New
CCND1(12443)_Down,VDR(22337)_Down	New
CCND1(12443)_Down,VDR(22337)_Up	New
CDH2(12558)_Up,MYC(17869)_Down	New
DKK1(13380)_Up,EGR1(13653)_Down	New
DKK1(13380)_Up,TNF(21926)_Down	New
EGR1(13653)_Down,EPAS1(13819)_Up	New
EGR1(13653)_Down,FOS(14281)_Down	New
EGR1(13653)_Down,IL1A(16175)_Up	New
EGR1(13653)_Down,MAPK1(26413)_Down	New
EGR1(13653)_Down,MAPK10(26414)_Down	New
EGR1(13653)_Down,SOS1(20662)_Down	New

EGR1(13653)_Down,VDR(22337)_Down	New
EGR1(13653)_Down,VDR(22337)_Up	New
EGR1(13653)_Down,WNT1(22408)_Up	New
EPAS1(13819)_Up,TNF(21926)_Down	New
IFNG(15978)_Up,MYC(17869)_Down	New
IL1A(16175)_Up,TNF(21926)_Down	New
MRE11A(17535)_Down,TNF(21926)_Down	New
MYC(17869)_Down,MAPK10(26414)_Down	New
MYC(17869)_Down,NKX2-2(18088)_Up	New
MYC(17869)_Down,PAX6(18508)_Up	New
MYC(17869)_Down,PKLR(18770)_Up	New
MYC(17869)_Down,SFRP1(20377)_Up	New
MYC(17869)_Down,TNF(21926)_Down	New
MYC(17869)_Down,VDR(22337)_Down	New
MYC(17869)_Down,WNT1(22408)_Up	New
PAX6(18508)_Up,TNF(21926)_Down	New
PDX1(18609)_Up,TNF(21926)_Down	New
PDX1(18609)_Up,TP53(22059)_Down	New
SOS1(20662)_Down,TNF(21926)_Down	New
TNF(21926)_Down,MAPK1(26413)_Down	New
TNF(21926)_Down,MAPK10(26414)_Down	New
TNF(21926)_Down,VDR(22337)_Down	New
TNF(21926)_Down,VDR(22337)_Up	New
TNF(21926)_Down,WNT1(22408)_Up	New
TP53(22059)_Down,VDR(22337)_Down	New

Supplementary Table 34. Disassembled relations derived from Top-50 impactful itemsets of Phosgene dataset in comparison 3 of undifferentiation control method

Disassembled Relations	References
ATF3(11910)_Up,MT1H(17750)_Up	New
MT1E(17748)_Up,MT1H(17750)_Up	New
ATF3(11910)_Up,MT1E(17748)_Up	New
MT1H(17750)_Up,TNFRSF12A(27279)_Up	New
ATF3(11910)_Up,TNFRSF12A(27279)_Up	New
MT1E(17748)_Up,TNFRSF12A(27279)_Up	New
TNFRSF12A(27279)_Up,TNFRSF12A(27279)_Up	New
KRT8(16691)_Up,MT1H(17750)_Up	New
ATF3(11910)_Up,KRT8(16691)_Up	New
MT1H(17750)_Up,SRXN1(76650)_Up	New
ATF3(11910)_Up,SRXN1(76650)_Up	New
MT1E(17748)_Up,SRXN1(76650)_Up	New
KRT8(16691)_Up,MT1E(17748)_Up	New
CLU(12759)_Up,MT1H(17750)_Up	New
ATF3(11910)_Up,CLU(12759)_Up	New
TNFRSF12A(27279)_Up,SRXN1(76650)_Up	New
CLU(12759)_Up,MT1E(17748)_Up	New
KRT8(16691)_Up,TNFRSF12A(27279)_Up	New
CLU(12759)_Up,TNFRSF12A(27279)_Up	New
MT1E(17748)_Up,SLC2A1(20525)_Up	New
MT1H(17750)_Up,SLC2A1(20525)_Up	New
ATF3(11910)_Up,SLC2A1(20525)_Up	New
MT1E(17748)_Up,TIMP1(21857)_Up	New
MT1H(17750)_Up,TIMP1(21857)_Up	New
ATF3(11910)_Up,TIMP1(21857)_Up	New
SLC2A1(20525)_Up,TNFRSF12A(27279)_Up	New
TIMP1(21857)_Up,TNFRSF12A(27279)_Up	New
MT1E(17748)_Up,SPNS2(216892)_Down	New
MT1H(17750)_Up,SPNS2(216892)_Down	New
ATF3(11910)_Up,SPNS2(216892)_Down	New

Supplementary Table 35. Disassembled relations derived from Top-50 impactful itemsets of Phosgene dataset in comparison 3 with a constant degree

Disassembled Relations	References
CDKN1A(12575)_Up,MYC(17869)_Up	New
CDKN1A(12575)_Up,PRICKLE1(106042)_Down	New
CDKN1A(12575)_Up,SLC2A1(20525)_Up	New
CDKN1A(12575)_Up,SOCS3(12702)_Up	New
MYC(17869)_Up,PRICKLE1(106042)_Down	New
MYC(17869)_Up,SLC2A1(20525)_Up	New
SLC2A1(20525)_Up,PRICKLE1(106042)_Down	New
SLC2A1(20525)_Up,SLC2A1(20525)_Up	New
SOCS3(12702)_Up,MYC(17869)_Up	New
SOCS3(12702)_Up,PRICKLE1(106042)_Down	New
SOCS3(12702)_Up,SLC2A1(20525)_Up	New
SOCS3(12702)_Up,SOCS3(12702)_Up	New

Supplementary Table 36. Disassembled relations derived from Top-50 impactful itemsets of Phosgene dataset in comparison 3 with various degrees

Disassembled Relations	References
CEBPB(12608)_Up,MYC(17869)_Up	[40]
EGR1(13653)_Up,MYC(17869)_Up	[36]
MYC(17869)_Up,THBS1(21825)_Up	[41]
SOCS3(12702)_Up,FOS(14281)_Up	[42]
SOCS3(12702)_Up,JUN(16476)_Up	[42]
CDKN1A(12575)_Up,TP53(22059)_Down	[43]*
CTNNB1(12387)_Down,MYC(17869)_Up	[44]*
LEF1(16842)_Down,MYC(17869)_Up	[45]*
MYC(17869)_Up,TP53(22059)_Down	[46]*
AGRN(11603)_Down,MYC(17869)_Up	New
APC(11789)_Down,CDKN1A(12575)_Up	New
APC(11789)_Down,MYC(17869)_Up	New
APC(11789)_Down,PRICKLE1(106042)_Down	New
APC(11789)_Down,SLC2A1(20525)_Up	New
APC(11789)_Down,SOCS3(12702)_Up	New
APC(11789)_Down,SUFU(24069)_Down	New
APC(11789)_Up,CDKN1A(12575)_Up	New
APC(11789)_Up,CEBPB(12608)_Up	New
APC(11789)_Up,IGFBP3(16009)_Down	New
APC(11789)_Up,IL6(16193)_Up	New
APC(11789)_Up,MYC(17869)_Up	New
APC(11789)_Up,PRICKLE1(106042)_Down	New
APC(11789)_Up,SERPINE1(18787)_Up	New
APC(11789)_Up,SLC2A1(20525)_Up	New
APC(11789)_Up,SOCS3(12702)_Up	New
APC(11789)_Up,SUFU(24069)_Down	New
APC(11789)_Up,THBS1(21825)_Up	New
APC(11789)_Up,VCAM1(22329)_Down	New
CDKN1A(12575)_Up,MYC(17869)_Up	New
CTNNB1(12387)_Down,SOCS3(12702)_Up	New
FIGF(14205)_Up,MYC(17869)_Up	New
FOS(14281)_Up,MYC(17869)_Up	New
IGFBP3(16009)_Down,MYC(17869)_Up	New
IL6(16193)_Up,MYC(17869)_Up	New
JUN(16476)_Up,MYC(17869)_Up	New
MYC(17869)_Up,PRICKLE1(106042)_Down	New
MYC(17869)_Up,SERPINE1(18787)_Up	New
MYC(17869)_Up,SLC2A1(20525)_Up	New
MYC(17869)_Up,SUFU(24069)_Down	New

MYC(17869)_Up,VANGL2(93840)_Down	New
MYC(17869)_Up,VCAM1(22329)_Down	New
SLC2A1(20525)_Up,TP53(22059)_Down	New
SOCS3(12702)_Up,FIGF(14205)_Up	New
SOCS3(12702)_Up,LEF1(16842)_Down	New
SOCS3(12702)_Up,MYC(17869)_Up	New
SOCS3(12702)_Up,TP53(22059)_Down	New
TP53(22059)_Down,PRICKLE1(106042)_Down	New
TP53(22059)_Down,SUFU(24069)_Down	New

*: Inconsistent with literature.

References

1. Granja, A.G., et al., *The viral protein A238L inhibits TNF-alpha expression through a CBP/p300 transcriptional coactivators pathway.* J Immunol, 2006. **176**(1): p. 451-62.
2. Barre, B., S. Avril, and O. Coqueret, *Opposite regulation of myc and p21waf1 transcription by STAT3 proteins.* J Biol Chem, 2003. **278**(5): p. 2990-6.
3. Zhao, Y., et al., *Acetylation of p53 at lysine 373/382 by the histone deacetylase inhibitor depsipeptide induces expression of p21(Waf1/Cip1).* Mol Cell Biol, 2006. **26**(7): p. 2782-90.
4. Ben-Yosef, T., et al., *Involvement of Myc targets in c-myc and N-myc induced human tumors.* Oncogene, 1998. **17**(2): p. 165-71.
5. De Fabiani, E., et al., *Coordinated control of cholesterol catabolism to bile acids and of gluconeogenesis via a novel mechanism of transcription regulation linked to the fasted-to-fed cycle.* J Biol Chem, 2003. **278**(40): p. 39124-32.
6. Rajabi, H.N., et al., *Effects of depletion of CREB-binding protein on c-Myc regulation and cell cycle G1-S transition.* J Biol Chem, 2005. **280**(1): p. 361-74.
7. Spencer, T.E., et al., *Steroid receptor coactivator-1 is a histone acetyltransferase.* Nature, 1997. **389**(6647): p. 194-8.
8. Cohen, J., et al., *Attenuated transforming growth factor beta signaling promotes nuclear factor-kappaB activation in head and neck cancer.* Cancer Res, 2009. **69**(8): p. 3415-24.
9. Zhang, H., et al., *Nocodazole-induced p53-dependent c-Jun N-terminal kinase activation reduces apoptosis in human colon carcinoma HCT116 cells.* J Biol Chem, 2002. **277**(46): p. 43648-58.
10. Wang, S. and W.S. El-Deiry, *p73 or p53 directly regulates human p53 transcription to maintain cell cycle checkpoints.* Cancer Res, 2006. **66**(14): p. 6982-9.
11. Zhao, L.Y., et al., *PCAF is a coactivator for p73-mediated transactivation.* Oncogene, 2003. **22**(51): p. 8316-29.
12. Forrester, K., et al., *Nitric oxide-induced p53 accumulation and regulation of inducible nitric oxide synthase expression by wild-type p53.* Proc Natl Acad Sci U S A, 1996. **93**(6): p. 2442-7.
13. Buschmann, T., et al., *p53 phosphorylation and association with murine double minute 2, c-Jun NH2-terminal kinase, p14ARF, and p300/CBP during the cell cycle and after exposure to ultraviolet irradiation.* Cancer Res, 2000. **60**(4): p. 896-900.
14. Ban, J., et al., *EWS-FLI1 suppresses NOTCH-activated p53 in Ewing's sarcoma.* Cancer Res, 2008. **68**(17): p. 7100-9.
15. Shih, A., et al., *Thyroid hormone promotes serine phosphorylation of p53 by mitogen-activated protein kinase.* Biochemistry, 2001. **40**(9): p. 2870-8.
16. Shirley, S.H., et al., *Transcriptional regulation of estrogen receptor-alpha by p53 in human breast cancer cells.* Cancer Res, 2009. **69**(8): p. 3405-14.
17. Pratt, M.A. and M.Y. Niu, *Bcl-2 controls caspase activation following a p53-dependent cyclin D1-induced death signal.* J Biol Chem, 2003. **278**(16): p. 14219-29.
18. Miao, F., et al., *In vivo chromatin remodeling events leading to inflammatory gene transcription under diabetic conditions.* J Biol Chem, 2004. **279**(17): p. 18091-7.
19. Aoki, M., et al., *Inhibition of the p53 tumor suppressor gene results in growth of human aortic vascular smooth muscle cells. Potential role of p53 in*

regulation of vascular smooth muscle cell growth. Hypertension, 1999. **34**(2): p. 192-200.

20. Alfano, D., et al., *Modulation of cellular migration and survival by c-Myc through the downregulation of urokinase (uPA) and uPA receptor.* Mol Cell Biol, 2010. **30**(7): p. 1838-51.
21. Grandori, C., et al., *c-Myc binds to human ribosomal DNA and stimulates transcription of rRNA genes by RNA polymerase I.* Nat Cell Biol, 2005. **7**(3): p. 311-8.
22. Jin, Z., et al., *Tobacco-specific nitrosamine 4-(methylnitrosamino)-1-(3-pyridyl)-1-butanone promotes functional cooperation of Bcl2 and c-Myc through phosphorylation in regulating cell survival and proliferation.* J Biol Chem, 2004. **279**(38): p. 40209-19.
23. Kirmizis, A., S.M. Bartley, and P.J. Farnham, *Identification of the polycomb group protein SU(Z)12 as a potential molecular target for human cancer therapy.* Mol Cancer Ther, 2003. **2**(1): p. 113-21.
24. Saxena, N.K., et al., *leptin-induced growth stimulation of breast cancer cells involves recruitment of histone acetyltransferases and mediator complex to CYCLIN D1 promoter via activation of Stat3.* J Biol Chem, 2007. **282**(18): p. 13316-25.
25. Heinrich, P.C., et al., *Principles of interleukin (IL)-6-type cytokine signalling and its regulation.* Biochem J, 2003. **374**(Pt 1): p. 1-20.
26. Jung, H., H.A. Seong, and H. Ha, *NM23-H1 tumor suppressor and its interacting partner STRAP activate p53 function.* J Biol Chem, 2007. **282**(48): p. 35293-307.
27. Yoon, K. and R.C. Smart, *C/EBPalpha is a DNA damage-inducible p53-regulated mediator of the G1 checkpoint in keratinocytes.* Mol Cell Biol, 2004. **24**(24): p. 10650-60.
28. Liu, T., et al., *Hypoxia induces p53-dependent transactivation and Fas/CD95-dependent apoptosis.* Cell Death Differ, 2007. **14**(3): p. 411-21.
29. Yoon, H., et al., *Gene expression profiling of isogenic cells with different TP53 gene dosage reveals numerous genes that are affected by TP53 dosage and identifies CSPG2 as a direct target of p53.* Proc Natl Acad Sci U S A, 2002. **99**(24): p. 15632-7.
30. Sampath, J., et al., *Mutant p53 cooperates with ETS and selectively up-regulates human MDR1 not MRP1.* J Biol Chem, 2001. **276**(42): p. 39359-67.
31. Santhanam, U., A. Ray, and P.B. Sehgal, *Repression of the interleukin 6 gene promoter by p53 and the retinoblastoma susceptibility gene product.* Proc Natl Acad Sci U S A, 1991. **88**(17): p. 7605-9.
32. Hellin, A.C., et al., *Nuclear factor - kappaB-dependent regulation of p53 gene expression induced by daunomycin genotoxic drug.* Oncogene, 1998. **16**(9): p. 1187-95.
33. Wells, J., et al., *Identification of novel pRb binding sites using CpG microarrays suggests that E2F recruits pRb to specific genomic sites during S phase.* Oncogene, 2003. **22**(10): p. 1445-60.
34. Tsai, E.Y., et al., *A lipopolysaccharide-specific enhancer complex involving Ets, Elk-1, Sp1, and CREB binding protein and p300 is recruited to the tumor necrosis factor alpha promoter in vivo.* Mol Cell Biol, 2000. **20**(16): p. 6084-94.
35. Lemberger, T., et al., *CREB has a context-dependent role in activity-regulated transcription and maintains neuronal cholesterol homeostasis.* FASEB J, 2008. **22**(8): p. 2872-9.
36. Krishnaraju, K., B. Hoffman, and D.A. Liebermann, *The zinc finger transcription factor Egr-1 activates macrophage differentiation in M1 myeloblastic leukemia cells.* Blood, 1998. **92**(6): p. 1957-66.
37. Krones-Herzig, A., E. Adamson, and D. Mercola, *Early growth response 1 protein, an upstream gatekeeper of the p53 tumor suppressor, controls replicative senescence.* Proc Natl Acad Sci U S A, 2003. **100**(6): p. 3233-8.
38. Srivastava, S., et al., *Estrogen decreases TNF gene expression by blocking JNK activity and the resulting production of c-Jun and JunD.* J Clin Invest, 1999. **104**(4): p. 503-13.
39. Mathiassen, S., et al., *Tumor-associated antigens identified by mRNA expression profiling induce protective anti-tumor immunity.* Eur J Immunol, 2001. **31**(4): p. 1239-46.
40. Sebastian, T., et al., *C/EBPbeta cooperates with RB:E2F to implement Ras(V12)-induced cellular senescence.* EMBO J, 2005. **24**(18): p. 3301-12.
41. Baudino, T.A., et al., *c-Myc is essential for vasculogenesis and angiogenesis during development and tumor progression.* Genes Dev, 2002. **16**(19): p. 2530-43.
42. Qin, H., et al., *Molecular mechanism of lipopolysaccharide-induced SOCS-3 gene expression in macrophages and microglia.* J Immunol, 2007. **179**(9): p. 5966-76.

43. Inoue, N., et al., *Cyclin-dependent kinase inhibitor, p21WAF1/CIP1, is involved in adipocyte differentiation and hypertrophy, linking to obesity, and insulin resistance.* J Biol Chem, 2008. **283**(30): p. 21220-9.

44. Calvisi, D.F., et al., *Activation of beta-catenin during hepatocarcinogenesis in transgenic mouse models: relationship to phenotype and tumor grade.* Cancer Res, 2001. **61**(5): p. 2085-91.

45. Reya, T., et al., *Wnt signaling regulates B lymphocyte proliferation through a LEF-1 dependent mechanism.* Immunity, 2000. **13**(1): p. 15-24.

46. Noronha, E.J., K.H. Sterling, and K.L. Calame, *Increased expression of Bcl-xL and c-Myc is associated with transformation by Abelson murine leukemia virus.* J Biol Chem, 2003. **278**(51): p. 50915-22.
